# Genetic diversity of *Entamoeba*: Novel ribosomal lineages from cockroaches

**DOI:** 10.1371/journal.pone.0185233

**Published:** 2017-09-21

**Authors:** Tetsuro Kawano, Mihoko Imada, Pennapa Chamavit, Seiki Kobayashi, Tetsuo Hashimoto, Tomoyoshi Nozaki

**Affiliations:** 1 Graduate School of Life and Environmental Sciences, University of Tsukuba, Tsukuba, Ibaraki, Japan; 2 Department of Parasitology, National Institute of Infectious Diseases, Shinjuku, Tokyo, Japan; 3 Graduate School of Medicine, The University of Tokyo, Bunkyo, Tokyo, Japan; 4 Department of Infectious Diseases, Keio University School of Medicine, Shinjuku, Tokyo, Japan; 5 Faculty of Medical Technology, Hauchiew University, Bangplee, Samutprakarn, Thailand; 6 Center for Computational Sciences, University of Tsukuba, Tsukuba, Ibaraki, Japan; National Cheng Kung University, TAIWAN

## Abstract

Our current taxonomic perspective on *Entamoeba* is largely based on small-subunit ribosomal RNA genes (SSU rDNA) from *Entamoeba* species identified in vertebrate hosts with minor exceptions such as *E*. *moshkovskii* from sewage water and *E*. *marina* from marine sediment. Other *Entamoeba* species have also been morphologically identified and described from non-vertebrate species such as insects; however, their genetic diversity remains unknown. In order to further disclose the diversity of the genus, we investigated *Entamoeba* spp. in the intestines of three cockroach species: *Periplaneta americana*, *Blaptica dubia*, and *Gromphadorhina oblongonota*. We obtained 134 *Entamoeba* SSU rDNA sequences from 186 cockroaches by direct nested PCR using the DNA extracts of intestines from cockroaches, followed by scrutinized BLASTn screening and phylogenetic analyses. All the sequences identified in this study were distinct from those reported from known *Entamoeba* species, and considered as novel *Entamoeba* ribosomal lineages. Furthermore, they were positioned at the base of the clade of known *Entamoeba* species and displayed remarkable degree of genetic diversity comprising nine major groups in the three cockroach species. This is the first report of the diversity of SSU rDNA sequences from *Entamoeba* in non-vertebrate host species, and should help to understand the genetic diversity of the genus *Entamoeba*.

## Introduction

The genus *Entamoeba* is an important taxonomic group consisting of parasitic species that reside in a variety of vertebrate and invertebrate hosts, and potentially free living species that are isolated from the environment. *E*. *histolytica* is one of the major causes of diarrheal diseases in tropical regions, which ranks fifth of DALY in 2015 [1]. Since other *Entamoeba* species generally lack virulence in humans, comparative biology, biochemistry, and genetics have been applied to the *Entamoeba* genus mainly to attempt to discover the virulence-related genes and to understand the evolution of *Entamoeba* pathogenicity in humans.

Genetic diversity of *E*. *histoltyica* from humans has been well investigated due to its medical importance. Clark and colleagues proposed to use “ribosomal lineages”, the nomenclature for newly discovered SSU rDNA sequences close enough to those from other *Entamoeba* species, but not convincingly considered to be from independent *Entamoeba* species [2–9]. In contrast, although quite a few *Entamoeba* species were identified at the molecular level from primates (*e*.*g*. *E*. *nuttalli*, and *E*. *gingivalis*), reptiles (*E*. *invadens*, *E*. *insolita*, and *E*. *terrapinae*), and environments (*E*. *moshkovskii*, *E*. *ecuadoriensis*, and *E*. *marina* [10]), the genetic diversity of the entire genus *Entamoeba* remains poorly understood. Other *Entamoeba* species have also been described, but only morphologically identified, from non-vertebrate hosts such as insects (*E*. *apis* [11], *E*. *philippinensis* [12] and *E*. *polypodia* [13]), leeches (*E*. *aulastomi* [14]), and protozoon (*E*. *paulista* [15]).

In order to better understand the genetic diversity of *Entamoeba* inhabiting invertebrate organisms, we investigated *Entamoeba* from cockroaches. Here we report SSU rDNA-based genetic diversity of *Entamoeba* from three cockroach species: one common house cockroach, *Periplaneta americana*, and two forest cockroaches, *Blaptica dubia* (orange-spotted cockroach, Guyana spotted cockroach, or Argentinian wood cockroach) and *Gromphadorhina oblongonota* (Madagascar forest hissing cockroach).

## Materials and methods

### Cockroach collection and isolation of intestinal contents

Three cockroach species were used in this study: *Periplaneta americana* (American cockroach), *Blaptica dubia* (Argentinian forest cockroach, Dubia cockroach) and *Gromphadorhina oblongonota* (Madagascar hissing cockroach). *P*. *americana* were collected from an apartment in Bangplee, located in an urban area of Samutprakarn, Thailand (13° 36' 0" N, 100° 36' 0" E) in April 21, 2016 and July 28, 2016 by manual capture (No specific permissions were required for field studies. The field studies did not involve endangered or protected species.). Individual bugs were identified as *P*. *americana* by their yellowish circular marking on the prothorax and were collected in two sampling periods. *B*. *dubia* and *G*. *oblongonota* (3–5 cm in size) were purchased from a pet shop in Tokushima, Japan (34° 4' 0" N, 134° 34' 0" E) where they were domestically bred. The cockroaches were dissected in order to isolate and excise their intestines. For the first batch of *P*. *americana* collected (Pa_01 to Pa_30), intestines isolated from 4 individual cockroaches were combined, and then ground in a sterile mortar and pestle in 2 ml of sterile normal saline; that is, sample Pa_01 contained the intestines of 4 cockroaches. For *P*. *americana* collected in the second period, *B*. *dubia* and *G*. *oblongonota* (Pa_31 to Pa_80, Bd_01 to Bd_22 and Go_01 to Go_14 respectively), the intestines were not combined and were ground separately.

### DNA extraction and amplification of SSU rDNA derived from *Entamoeba*

DNA was extracted from approximately 500 μL of the ground intestine(s) using DNeasy Blood and Tissue kit (QIAGEN, Tokyo, Japan). A fragment corresponding to *Entamoeba* SSU rDNA was amplified by nested PCR using DNA extracted from the isolated cockroach intestine(s). In the first round of PCR, an approximately 1,950 bp long fragment corresponding to SSU rDNA was amplified using eukaryotic universal oligonucleotide primers specific for SSU rDNA (EukA: 5'-AACCTGGTTGATCCTGCCAGT-3' and EukB: 5'-TGATCCTTCTGCAGGTTCACCTAC-3'; [16]) by Tks Gflex DNA Polymerase (TaKaRa, Shiga, Japan). PCR conditions consisted of 30 cycles of denaturation at 94°C for 22 seconds, annealing at 42°C for 1 minute and extension at 72°C for 1 minute. One μL of PCR products were used as templates of the second round PCR. In the second round of PCR, an approximately 1,900 bp fragment of *Entamoeba* SSU rDNA was selectively amplified using oligonucleotide primers specific for *Entamoeba* SSU rDNA (01F: 5’-GCCAGTATTATATGCTGA-3’ and 01R: 5’-CCTTGTTACGACTTCTCCTT-3’). PCR conditions consisted of 30 cycles of denaturation at 94°C for 22 seconds, annealing at 52°C for 1 minute and extension at 72°C for 1 minute.

### Sequencing and screening of SSU rDNA of *Entamoeba* from cockroaches

The amplicons obtained from the second round PCR were cloned into pCRTM-Blunt II-TOPO (Thermo Fisher Scientific, Waltham, Massachusetts, USA) and the plasmids were transfected into competent *Escherichia coli* DH5α cells. Five to twenty colonies were examined by PCR using the universal oligonucleotide primers M13F/R (5'-GTAAAACGACGGCCAGTG-3' and 5'-CAGGAAACAGCTATGACCATG-3') to confirm if an insert is present in the plasmids from the bacterial colonies. After purification of plasmids, an insert of each plasmid was fully sequenced in both directions with M13F, M13R, M13Mid1 (5’-TACTTTGAATAAATACGAGTGTT-3’), and M13Mid2 (5’-TCCCGTGTTGAGTCAAATTAA-3’) primers. The latter two primers correspond to 18S rRNA gene. The sequences were examined by BLASTn [17] search against non-redundant (nr) nucleotide database of NCBI with default parameters to verify whether they only show highest similarity with *Entamoeba*. When needed, phylogenetic analysis (described below) was also used. Sequence reads were assembled using CLC Genomics Workbench Version 8.5.1 (Qiagen Aahus A/S, Aahus C, Denmark).

### Molecular phylogenetic analysis

Molecular phylogenetic analysis was performed to determine the relationship of cockroach-derived *Entamoeba* SSU rDNA with other eukaryotic organisms including other known *Entamoeba* species and Archamoebae. Analyses were performed as follows: 1) Sequences were aligned by MAFFT v7.187 [18], 2) aligned nucleotide sites were selected by Gblocks [19] and manual inspection using SeaView 4.6 [20], 3) Maximum-likelihood (ML) tree was inferred by RAxML 8.1.5 [21] with General Time-Reversible (GTR) + gamma substitution model. Statistical confidence of ML trees was evaluated with bootstrap proportions of the trees from 100 or 1,000 replicates for screening and detailed analyses, respectively. In the screening, when a sequence analyzed showed monophyly with other known *Entamoeba* species, it was considered to be included in the *Entamoeba* genus.

## Results and discussion

### A total of 134 *Entamoeba* SSU rDNA sequences were obtained from 186 cockroaches

**Fig 1:**
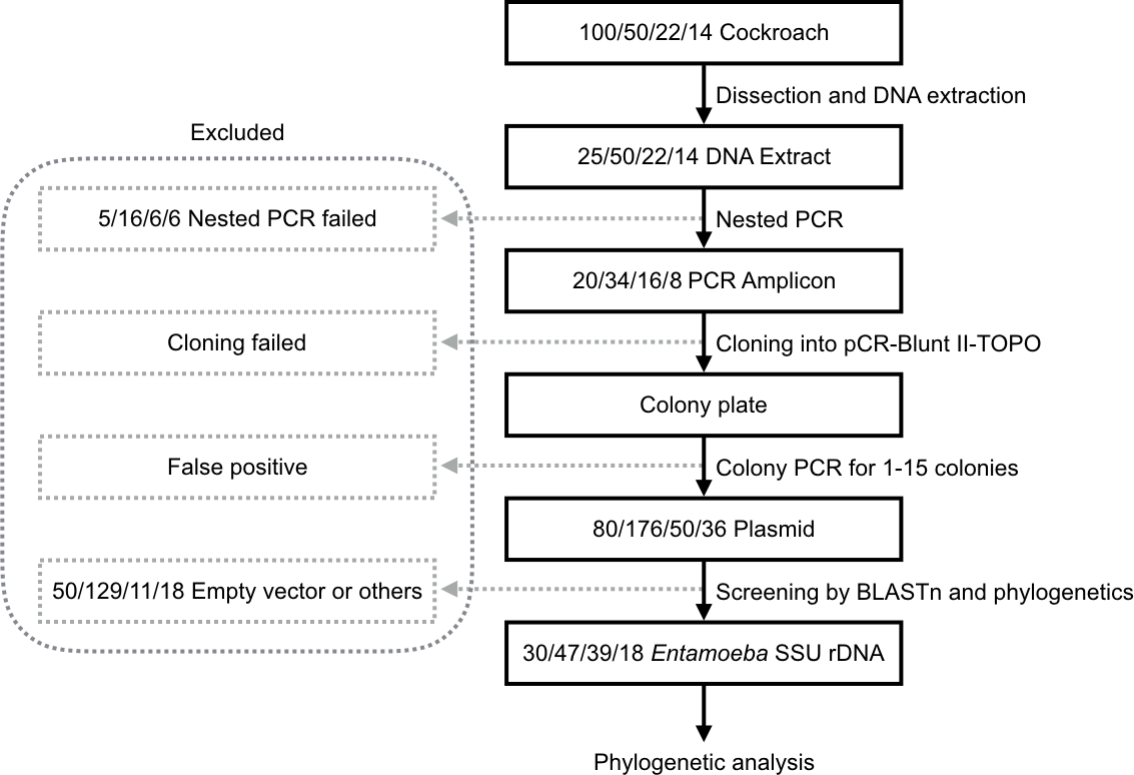
Flow diagram depicting experimental procedures and the number of analyzed samples. The numbers in rectangles indicate those of samples from *P*. *americana* (first sampling), *P*. *americana* (second sampling), *B*. *dubia* and *G*. *oblongonota*, respectively. For samples from the first sampling of *P*. *americana*, the intestines from 4 cockroaches were pooled.

The workflow of acquisition and screening of *Entamoeba* SSU rDNA genes from cockroaches is summarized in Fig 1. In brief, we isolated and purified DNA from the intestines of 186 cockroaches (150 *P*. *americana*, 22 *B*. *dubia*, and 14 *G*. *oblongonota*), and SSU rDNA was amplified by nested PCR. Nested PCR was successful for 54, 16, and 8 samples, respectively. The plasmids that contained nested PCR products (256, 50 and, 36 from each cockroach group) were obtained and sequenced. Subsequently, BLASTn search and phylogenetic analyses were performed to exclude non-*Entamoeba* SSU rDNA sequences. Finally, 77, 39, and 18 *Entamoeba* SSU rDNA sequences were subjected to further analyses (Table 1).

**Table 1:** The list of the sequences used in this study.

#	Sequence ID	Source	Cockroach ID	Colony ID	Accession No
1	Bd_06–2	*B*. *dubia*	6	2	LC259314
2	Bd_06–10	*B*. *dubia*	6	10	LC259315
3	Bd_08–1	*B*. *dubia*	8	1	LC259316
4	Bd_08–7	*B*. *dubia*	8	7	LC259317
5	Bd_09–1	*B*. *dubia*	9	1	LC259318
6	Bd_09–2	*B*. *dubia*	9	2	LC259319
7	Bd_09–3	*B*. *dubia*	9	3	LC259320
8	Bd_10–1	*B*. *dubia*	10	1	LC259321
9	Bd_10–2	*B*. *dubia*	10	2	LC259322
10	Bd_10-2b	*B*. *dubia*	10	2b	LC259323
11	Bd_11–1	*B*. *dubia*	11	1	LC259324
12	Bd_11–2	*B*. *dubia*	11	2	LC259325
13	Bd_11–6	*B*. *dubia*	11	6	LC259326
14	Bd_12–2	*B*. *dubia*	12	2	LC259327
15	Bd_13–1	*B*. *dubia*	13	1	LC259328
16	Bd_13–4	*B*. *dubia*	13	4	LC259329
17	Bd_13–5	*B*. *dubia*	13	5	LC259330
18	Bd_14–1	*B*. *dubia*	14	1	LC259331
19	Bd_14–2	*B*. *dubia*	14	2	LC259332
20	Bd_15–2	*B*. *dubia*	15	2	LC259333
21	Bd_15–3	*B*. *dubia*	15	3	LC259334
22	Bd_15–4	*B*. *dubia*	15	4	LC259335
23	Bd_16–1	*B*. *dubia*	16	1	LC259336
24	Bd_16–2	*B*. *dubia*	16	2	LC259337
25	Bd_16–3	*B*. *dubia*	16	3	LC259338
26	Bd_17–2	*B*. *dubia*	17	2	LC259339
27	Bd_17–3	*B*. *dubia*	17	3	LC259340
28	Bd_18–6	*B*. *dubia*	18	6	LC259341
29	Bd_18–7	*B*. *dubia*	18	7	LC259342
30	Bd_18–8	*B*. *dubia*	18	8	LC259343
31	Bd_19–5	*B*. *dubia*	19	5	LC259344
32	Bd_19–6	*B*. *dubia*	19	6	LC259345
33	Bd_20–1	*B*. *dubia*	20	1	LC259346
34	Bd_20–2	*B*. *dubia*	20	2	LC259347
35	Bd_21–2	*B*. *dubia*	21	2	LC259348
36	Bd_21–3	*B*. *dubia*	21	3	LC259349
37	Bd_22–1	*B*. *dubia*	22	1	LC259350
38	Bd_22–2	*B*. *dubia*	22	2	LC259351
39	Bd_22–3	*B*. *dubia*	22	3	LC259352
40	Go_06–1	*G*. *oblongonota*	6	1	LC259353
41	Go_06–9	*G*. *oblongonota*	6	9	LC259354
42	Go_07–1	*G*. *oblongonota*	7	1	LC259355
43	Go_07–5	*G*. *oblongonota*	7	5	LC259356
44	Go_07–6	*G*. *oblongonota*	7	6	LC259357
45	Go_07–8	*G*. *oblongonota*	7	8	LC259358
46	Go_08–1	*G*. *oblongonota*	8	1	LC259359
47	Go_09–2	*G*. *oblongonota*	9	2	LC259360
48	Go_09–3	*G*. *oblongonota*	9	3	LC259361
49	Go_09–4	*G*. *oblongonota*	9	4	LC259362
50	Go_10–1	*G*. *oblongonota*	10	1	LC259363
51	Go_10–3	*G*. *oblongonota*	10	3	LC259364
52	Go_11–3	*G*. *oblongonota*	11	3	LC259365
53	Go_11–5	*G*. *oblongonota*	11	5	LC259366
54	Go_13–5	*G*. *oblongonota*	13	5	LC259367
55	Go_14–2	*G*. *oblongonota*	14	2	LC259368
56	Go_14–3	*G*. *oblongonota*	14	3	LC259369
57	Go_14–4	*G*. *oblongonota*	14	4	LC259370
58	Pa_02–2	*P*. *americana*	2	2	LC259371
59	Pa_02–3	*P*. *americana*	2	3	LC259372
60	Pa_02–4	*P*. *americana*	2	4	LC259373
61	Pa_03–1	*P*. *americana*	3	1	LC259374
62	Pa_03–3	*P*. *americana*	3	3	LC259375
63	Pa_03–4	*P*. *americana*	3	4	LC259376
64	Pa_04–1	*P*. *americana*	4	1	LC259377
65	Pa_06–2	*P*. *americana*	6	2	LC259378
66	Pa_07–2	*P*. *americana*	7	2	LC259379
67	Pa_08–1	*P*. *americana*	8	1	LC259380
68	Pa_08–2	*P*. *americana*	8	2	LC259381
69	Pa_08–3	*P*. *americana*	8	3	LC259382
70	Pa_08–4	*P*. *americana*	8	4	LC259383
71	Pa_10–4	*P*. *americana*	10	4	LC259384
72	Pa_14–4	*P*. *americana*	14	4	LC259385
73	Pa_14–6	*P*. *americana*	14	6	LC259386
74	Pa_16–1	*P*. *americana*	16	1	LC259387
75	Pa_17–1	*P*. *americana*	17	1	LC259388
76	Pa_19–1	*P*. *americana*	19	1	LC259389
77	Pa_19–2	*P*. *americana*	19	2	LC259390
78	Pa_19–3	*P*. *americana*	19	3	LC259391
79	Pa_21–2	*P*. *americana*	21	2	LC259392
80	Pa_22–3	*P*. *americana*	22	3	LC259393
81	Pa_22–4	*P*. *americana*	22	4	LC259394
82	Pa_24–1	*P*. *americana*	24	1	LC259395
83	Pa_24–2	*P*. *americana*	24	2	LC259396
84	Pa_24–3	*P*. *americana*	24	3	LC259397
85	Pa_26–3	*P*. *americana*	26	3	LC259398
86	Pa_27–2	*P*. *americana*	27	2	LC259399
87	Pa_27–4	*P*. *americana*	27	4	LC259400
88	Pa_33–1	*P*. *americana*	33	1	LC259401
89	Pa_33–3	*P*. *americana*	33	3	LC259402
90	Pa_33–4	*P*. *americana*	33	4	LC259403
91	Pa_39–1	*P*. *americana*	39	1	LC259404
92	Pa_39–5	*P*. *americana*	39	5	LC259405
93	Pa_47–1	*P*. *americana*	47	1	LC259406
94	Pa_47–2	*P*. *americana*	47	2	LC259407
95	Pa_47–3	*P*. *americana*	47	3	LC259408
96	Pa_47–4	*P*. *americana*	47	4	LC259409
97	Pa_49–3	*P*. *americana*	49	3	LC259410
98	Pa_49–4	*P*. *americana*	49	4	LC259411
99	Pa_49–13	*P*. *americana*	49	13	LC259412
100	Pa_49–14	*P*. *americana*	49	14	LC259413
101	Pa_49–15	*P*. *americana*	49	15	LC259414
102	Pa_49–16	*P*. *americana*	49	16	LC259415
103	Pa_49–17	*P*. *americana*	49	17	LC259416
104	Pa_49–18	*P*. *americana*	49	18	LC259417
105	Pa_49–19	*P*. *americana*	49	19	LC259418
106	Pa_50–2	*P*. *americana*	50	2	LC259419
107	Pa_50–4	*P*. *americana*	50	4	LC259420
108	Pa_50–11	*P*. *americana*	50	11	LC259421
109	Pa_50–12	*P*. *americana*	50	12	LC259422
110	Pa_50–19	*P*. *americana*	50	19	LC259423
111	Pa_57–2	*P*. *americana*	57	2	LC259424
112	Pa_57–3	*P*. *americana*	57	3	LC259425
113	Pa_57–5	*P*. *americana*	57	5	LC259426
114	Pa_61–2	*P*. *americana*	61	2	LC259427
115	Pa_61–4	*P*. *americana*	61	4	LC259428
116	Pa_62–1	*P*. *americana*	62	1	LC259429
117	Pa_62–3	*P*. *americana*	62	3	LC259430
118	Pa_62–11	*P*. *americana*	62	11	LC259431
119	Pa_62–14	*P*. *americana*	62	14	LC259432
120	Pa_62–15	*P*. *americana*	62	15	LC259433
121	Pa_62–17	*P*. *americana*	62	17	LC259434
122	Pa_62–19	*P*. *americana*	62	19	LC259435
123	Pa_63–2	*P*. *americana*	63	2	LC259436
124	Pa_63–3	*P*. *americana*	63	3	LC259437
125	Pa_63–4	*P*. *americana*	63	4	LC259438
126	Pa_64–1	*P*. *americana*	64	1	LC259439
127	Pa_64–2	*P*. *americana*	64	2	LC259440
128	Pa_64–3	*P*. *americana*	64	3	LC259441
129	Pa_64–4	*P*. *americana*	64	4	LC259442
130	Pa_79–4	*P*. *americana*	79	4	LC259443
131	Pa_80–1	*P*. *americana*	80	1	LC259444
132	Pa_80–2	*P*. *americana*	80	2	LC259445
133	Pa_80–3	*P*. *americana*	80	3	LC259446
134	Pa_80–4	*P*. *americana*	80	4	LC259447

### *Entamoeba* SSU rDNA sequences from cockroaches are extremely heterogeneous, divergent from the reported sequences of known *Entamoeba* species, and composed of nine major groups

**Fig 2:**
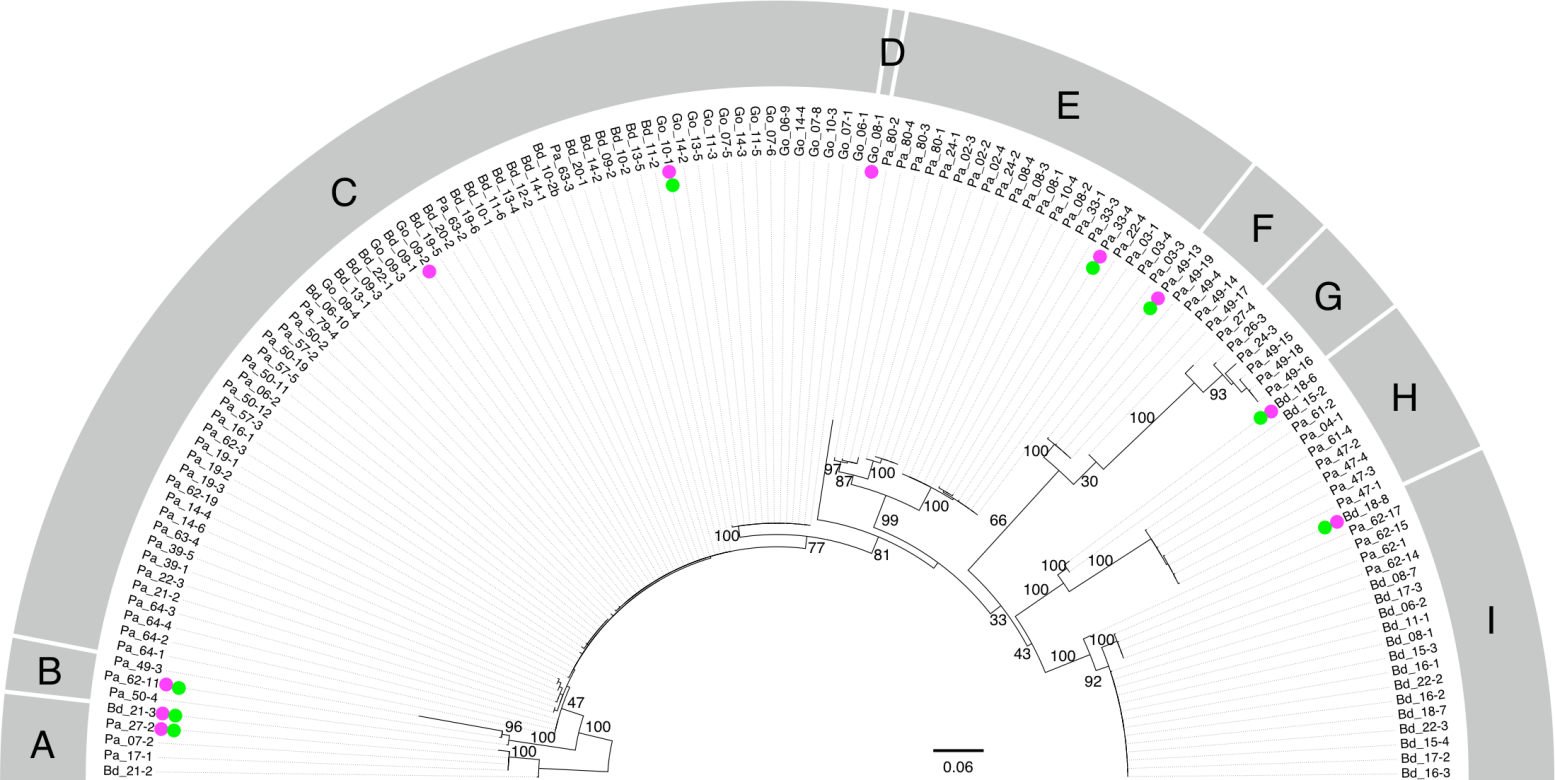
SSU rDNA-based phylogenetic tree of 134 *Entamoeba* sequences from cockroaches. SSU rDNA sequences were aligned using MAFFT v7.187. Unambiguously aligned sequences composed of 1,023 nucleotides were selected by Gblocks and manual inspection. Maximum-likelihood (ML) tree was inferred by RAxML 8.1.17 using GTRGAMMA model. The number of bootstrap pseudoreplicate trees was 1,000. ML tree was visualized using FigTree 1.4.0 and Keynote 6.6.2. Bootstrap values for major nodes are shown on each node. Nine groups (A to I) were shown to be monophyletic with high bootstrap support values. Representative sequences of each group used in Fig 3 or Fig 4 are indicated by green circles or magenta circles, respectively.

All *Entamoeba* SSU rDNA sequences from cockroaches are divergent from the reported sequences from known *Entamoeba* species. An unrooted phylogenetic tree was inferred by Maximum-likelihood (ML) method using 134 cockroach-derived *Entamoeba* SSU rDNA sequences (Fig 2). The 134 sequences were segregated into 9 groups (A-I), each of which was supported by good bootstrap values (> 70%), with exceptions for branching at A-B/C-I (47%), F/G (66%), H/I (43%) and F-G/H-I (33%).

### Phylogenetic position of *Entamoeba* SSU rDNA sequences from cockroaches in eukaryotes

**Fig 3:**
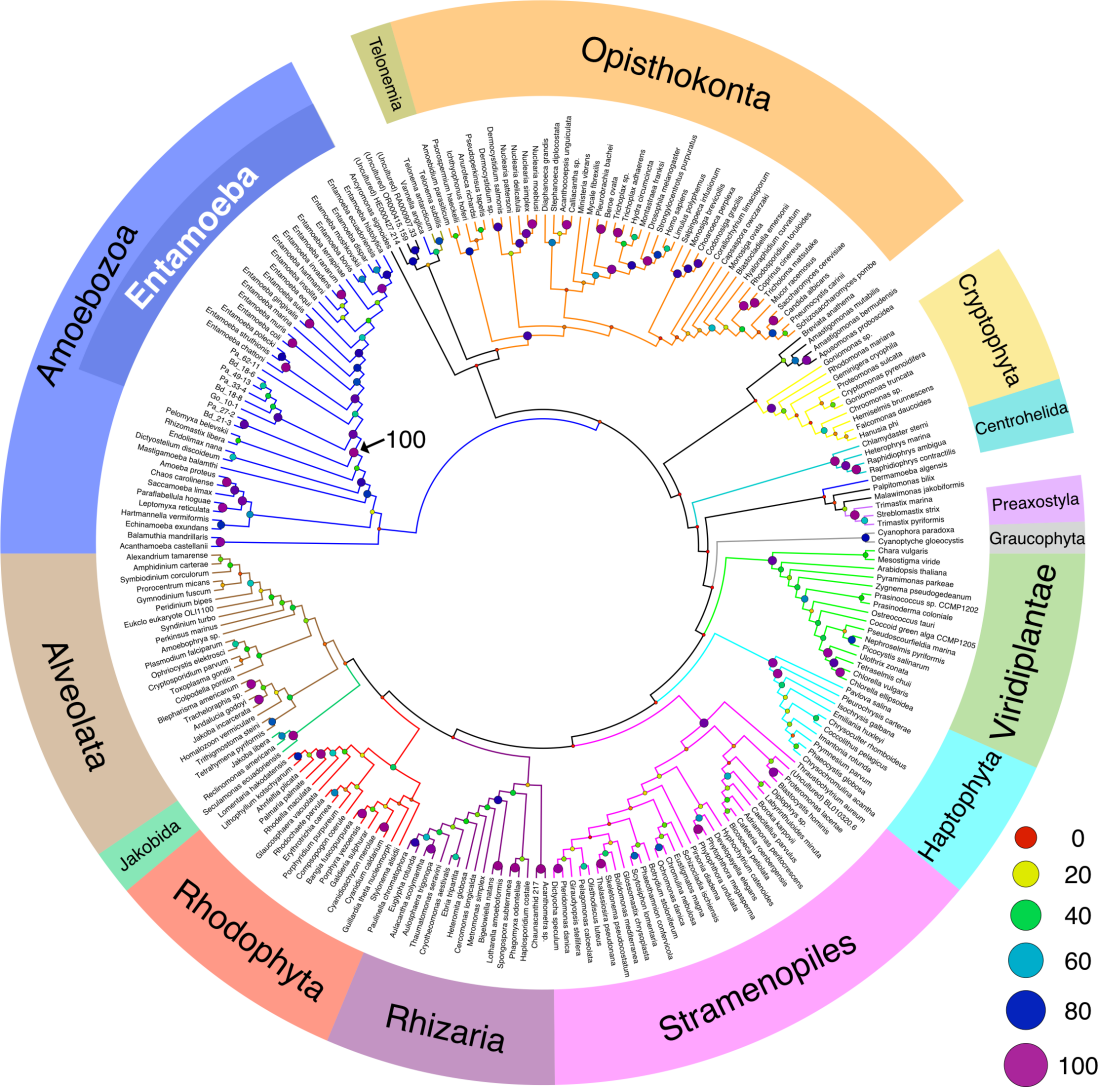
SSU rDNA-based cladogram of major eukaryotic supergroups including representative cockroach-derived *Entamoeba*. SSU rDNA sequences were aligned using MAFFT v7.187. Unambiguously aligned sequences composed of 914 nucleotides were selected by Gblocks and manual inspection. Maximum-likelihood (ML) tree was inferred by RAxML 8.1.17 using GTRGAMMA model. The number of bootstrap pseudoreplicate trees was 1,000. ML tree was visualized as a cladogram using FigTree 1.4.0 and Keynote 6.6.2. Note that all representative sequences of cockroach-derived *Entamoeba* are new *Entamoeba* ribosomal lineages, and their monophyly was supported by the high bootstrap value (100%; black arrow). The size and colors of circles at the nodes indicate the approximate bootstrap value.

To examine the phylogenetic position of these cockroach-derived *Entamoeba* sequences, the cladogram was reconstructed using SSU rDNA dataset composing of major eukaryotic supergroups and eight representative sequences from Group A to I from cockroach-derived *Entamoeba* (Fig 3; marked with green circles in Fig 2; group D and G were omitted because of their high evolutionary rates). The monophyly of the clade comprising cockroach-derived *Entamoeba* (Pa_61–11, Bd_18–6, Pa_49–13, Pa_33–4, Bd_18–8, Go_10–1, Pa_27–2, and Bd_21–3) and other *Entamoeba* species were strongly supported (Fig 3; black arrow). This clade is nested within the node that contains other Archamoebae (*Pelomyxa belevskii*, *Rhizomastix libera*, *Mastigamoeba balamuthi* and *Endolimax nana*) and *Dictyostelium discoideum*, with high bootstrap support (Fig 3; black arrow). Although the monophyly of Amoebozoa was not supported by the bootstrap value, these data are consistent with the premise that the newly identified *Entamoeba* sequences are from novel *Entamoeba* ribosomal lineages.

### Polymorphism of *Entamoeba* SSU rDNA sequences from cockroaches

As shown above, cockroach-derived *Entamoeba* SSU rDNA sequences were categorized into 9 groups (Fig 2). Groups A, B, D, E, H, and I were independent and well separated clades with almost maximum statistical support (bootstrap proportion: > 99%). Groups A, H and I were composed of sequences of the amoebae from both *P*. *americana* (11 of 77 *P*. *americana*-derived *Entamoeba* sequences) and *B*. *dubia* (4/20), whereas groups B and E were exclusively from *P*. *americana* (24/77), and group D was only from *G*. *oblongonota* (1/18).

**Fig 4:**
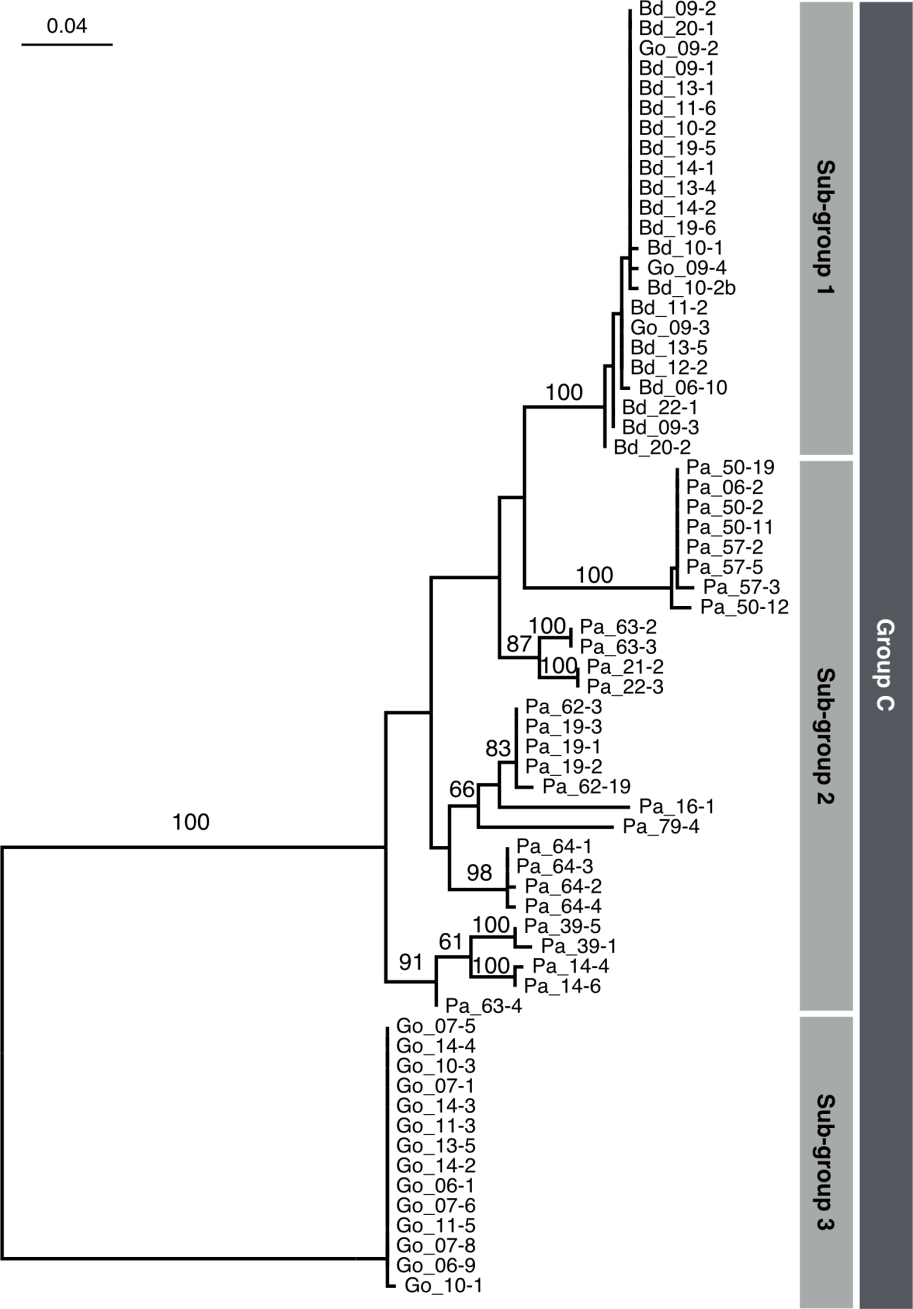
Phylogenetic tree of SSU rDNA of Group C sequences of cockroach-derived *Entamoeba*. SSU rDNA sequences were aligned using MAFFT v7.187. Unambiguously aligned sequences composed of 1,224 nucleotides were selected by Gblocks and manual inspection. Maximum-likelihood (ML) tree was inferred by RAxML 8.1.17 using GTRGAMMA model. The number of bootstrap pseudoreplicate trees was 1,000. ML tree was visualized using FigTree 1.4.0 and Keynote 6.6.2. Bootstrap values for major nodes are shown on each node.

Group C represents the largest group of cockroach-derived *Entamoeba* and consists of 65 sequences (49% of all cockroach-derived *Entamoeba* sequences) from *P*. *americana* (28/77), *B*. *dubia* (20/24) and *G*. *oblongonota* (17/18). This group can be divided into three sub-groups; sub-group 1 consists of 20 sequences from *B*. *dubia* and three sequences from *G*. *oblongonota*, sub-group 2 consists of 28 sequences derived only from *P*. *americana*, and sub-group 3 consists of 14 sequences derived only from *G*. *oblongonota* (Fig 4). Note that monophyly of sub-groups 1 and 3 is well supported by the highest bootstrap proportion, while sub-group 2 does not form monophyly and may consist of multiple divergent sub-groups.

Groups F and G were defined by a separate analysis using amoebae only from *P*. *americana*. In the tree excluding amoebae from *G*. *oblongonota* and *B*. *dubia*, each of the groups F and G formed an independent clade with high statistical support value (S1 Fig). Whereas in the tree including amoebae from *G*. *oblongonota* and *B*. *dubia*, monophyly of group F was not reconstructed, but instead amoebae of groups F and G were shown to be monophyletic with weak statistical support value (66%). Since branch lengths leading to the amoebae of groups F and G are long, it is possible that these amoebae were attracted in the tree in Fig 2 by a long branch attraction artifact.

### The genetic diversity of cockroach-derived *Entamoeba* among all *Entamoeba* and Archamoebae

**Fig 5:**
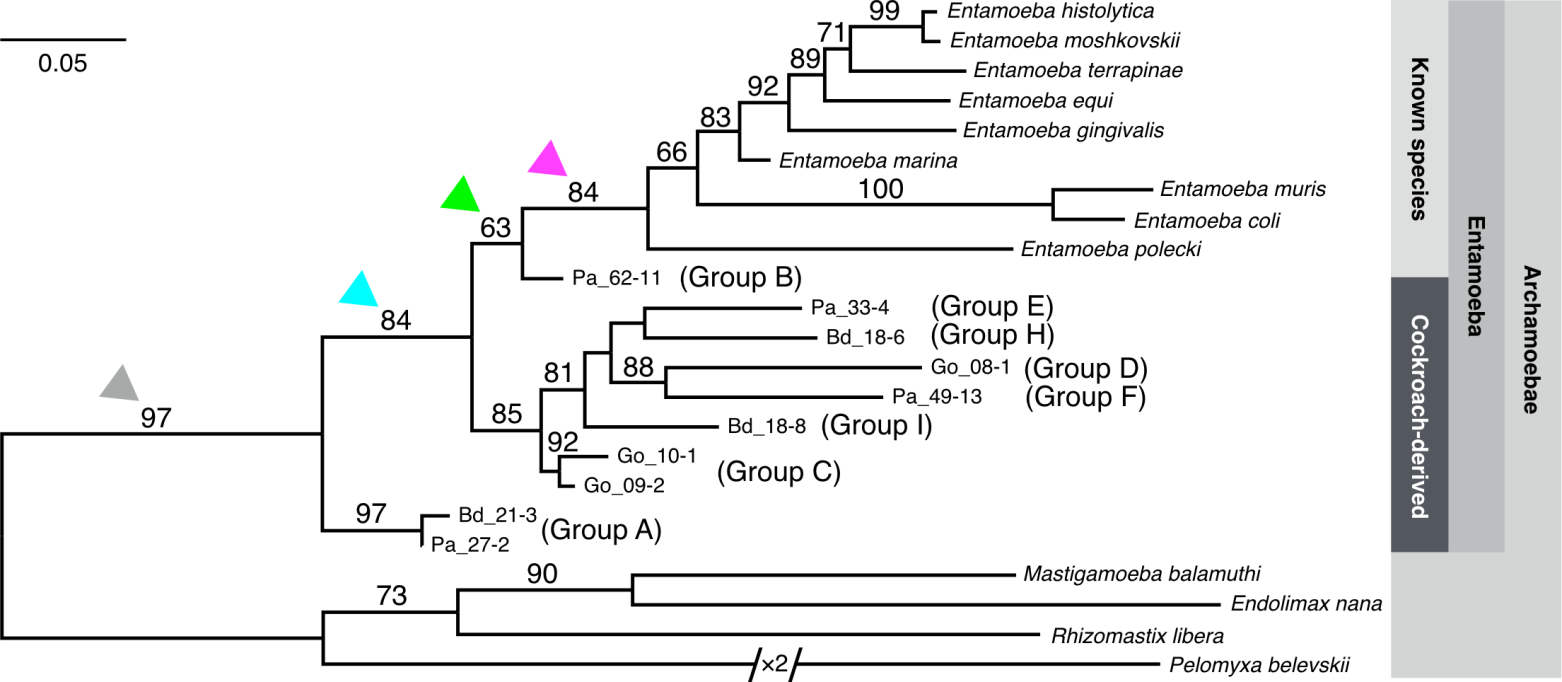
Phylogenetic tree of SSU rDNA of representative cockroach-derived *Entamoeba* ribosomal lineages and other Archamoebae species. SSU rDNA sequences were aligned using MAFFT v7.187. Well-aligned 1,224 nucleotide positions were selected by Gblocks and manual operation. Maximum-likelihood (ML) tree was inferred by RAxML 8.1.17 using GTRGAMMA model. The number of bootstrap pseudoreplicate trees was 1,000. ML tree was visualized using FigTree 1.4.0 and Keynote 6.6.2. Bootstrap values (over 60%) are shown on each branch. Monophyly of *Entamoeba* is strongly supported with high bootstrap value (97%; gray arrow head). Commencing with Pa_27–2 and Bd_21–3, all cockroach-derived *Entamoeba* are positioned at the base of *Entamoeba* clade.

To obtain better resolution of all *Entamoeba* including cockroach-derived amoebae and Archamoebae species, the ML tree of the representative taxa was inferred (Fig 5). In the resulting tree, the monophyly of *Entamoeba* comprising representative cockroach-derived *Entamoeba* and 9 known *Entamoeba* species (*E*. *histolytica*, *E*. *moshkovskii*, *E*. *terrapinae*, *E*. *equi*, *E*. *gingivalis*, *E*. *marina*, *E*. *muris*, *E*. *coli*, and *E*. *polecki*) are strongly supported with bootstrap value (97%; gray arrow head). The monophyly of known *Entamoeba* is well supported (84%; magenta arrow head) and their inter-specific relationships are also unequivocally reconstructed (66% to 100% bootstrap values). The cockroach-derived *Entamoeba* forms three major independent clades: Group A, Group B, and the rest, Group C to I. All three clades are positioned basal to known *Entamoeba* species. Group A consists of the most basal ribosomal lineages of cockroach-derived *Entamoeba*, and the levels of observed divergence among them were relatively lower than those of other groups. On the other hand, group B comprises of members isolated exclusively from *P*. *americana*, is a sister group to known vertebrate-derived *Entamoeba*, although its statistical support was weak (63%; green arrow head). Group C to I forms a single largest statistically supported clade and is sister to the clade comprised of group B and known *Entamoeba* (84; cyan arrow head).

### Polymorphism of *Entamoeba* identified in a single cockroach and presence of cockroach species-specific and common *Entamoeba* groups

For all the samples except for the first set of *P*. *americana* specimens (*i*.*e*., Pa_02 to Pa_27), single cockroaches were analyzed without cockroaches being pooled. Multiple groups were identified occasionally in a single *P*. *americana* (Pa_33 to Pa_80) sample (Table 2). The highest number of *Entamoeba* groups found in a single cockroach was 3 (Pa_49 and Pa_62), while 79% (22 of 28) of *P*. *americana* were found to harbor only a single *Entamoeba* group. *B*. *dubia* (31%; 5 of 16 cockroaches) had two *Entamoeba* groups (Table 3). In contrast, no *G*. *oblongonota* harboring multiple groups was found, although the sample size was small (8 cockroaches and 18 sequences; Table 4).

**Table 2:** The number of *Entamoeba* groups found in each *P*. *americana*.

Source		A	B	C	D	E	F	G	H	I
Pa	02					3				
Pa	03					3				
Pa	04								1	
Pa	06			1						
Pa	07	1								
Pa	08					4				
Pa	10					1				
Pa	14			2						
Pa	16			1						
Pa	17	1								
Pa	19			3						
Pa	21			1						
Pa	22			1		1				
Pa	24					2		1		
Pa	26							1		
Pa	27	1						1		
Pa	33					3				
Pa	39			2						
Pa	47								4	
Pa	49		1				5	3		
Pa	50		1	4						
Pa	57			3						
Pa	61								2	
Pa	62		1	2						4
Pa	63			3						
Pa	64			4						
Pa	79			1						
Pa	80					4				
Total		3	3	28	0	21	5	6	7	4

**Table 3:** The number of *Entamoeba* groups found in each *G*. *oblongonota*.

Source		A	B	C	D	E	F	G	H	I
Go	06			2						
Go	07			4						
Go	08				1					
Go	09			3						
Go	10			2						
Go	11			2						
Go	13			1						
Go	14			3						
Total		0	0	17	1	0	0	0	0	0

**Table 4:** The number of *Entamoeba* groups found in each *B*. *dubia*.

Source		A	B	C	D	E	F	G	H	I
Bd	06			1						1
Bd	08									2
Bd	09			3						
Bd	10			3						
Bd	11			2						1
Bd	12			1						
Bd	13			3						
Bd	14			2						
Bd	15								1	2
Bd	16									3
Bd	17									2
Bd	18								1	2
Bd	19			2						
Bd	20			2						
Bd	21	2								
Bd	22			1						2
Total		2	0	20	0	0	0	0	2	15

Group C was the most common and highly shared group discovered from three cockroach species. The 23 sequences consisting the sub-group 1 of group C were mutually very similar (> 99.5% mutual positional identity; Table 5). In other words, almost identical *Entamoeba* sequences that belong to group C sub-group 1 were discovered from both the forest cockroaches (*B*. *dubia* and *G*.*oblongonota*), suggestive of conservation of genetic traits of this sub-group despite distinct host species and geographic origins.

**Table 5:** Sequence percentage identities among representative members of the clades in group C.

	Go_09–2	Bd_20–2	Pa_50–19	Pa_63–4	Go_06–9
**Go_09–2**	100	99.5	89.9	89.6	86.8
**Bd_20–2**		100	90.2	90.1	87.3
**Pa_50–19**			100	88.1	84.4
**Pa_63–4**				100	88.0
**Go_06–9**					100

Identities were calculated by EMBOSS Needle (http://www.ebi.ac.uk/Tools/psa/emboss_needle/).

### Discovery of novel *Entamoeba* ribosomal lineages in cockroaches expands our understanding of genetic diversity of *Entamoeba*

We have demonstrated that the genetic diversity of *Entamoeba* derived from three cockroach species overwhelms that of previous reports which described diversity among species found in vertebrates, as well as the potential free living species (*E*. *moshkovskii* and *E*. *marina*). Despite our repeated attempts, we were unable to cultivate cockroach-derived *Entamoeba* and thus to get sufficient amount of genomic DNA or RNA for whole genome and transcriptome analyses. Hence, the genome of cockroach-derived *Entamoeba* remains to be elucidated.

## Supporting information

S1 Fig SSU rDNA-based phylogenetic tree of 77 *Entamoeba* sequences from *P*. *americana*. SSU rDNA sequences were aligned using MAFFT v7.187. Unambiguously aligned sequences composed of 1069 nucleotides were selected by Gblocks and manual inspection. Maximum-likelihood (ML) tree was inferred by RAxML 8.1.17 using GTRGAMMA model. The number of bootstrap pseudoreplicate trees was 100. ML tree was visualized using FigTree 1.4.0 and Keynote 6.6.2. Bootstrap values for major nodes are shown on each node. Nine groups (A-I) were shown to be monophyletic with high bootstrap support values.
(TIF)

S2 Fig SSU rDNA-based phylogenetic tree of 134 *Entamoeba* sequences from cockroaches using different substitution model. In order to ensure consistency of the result shown in Fig 2, the phylogenetic tree was constructed with other model. SSU rDNA sequences were aligned using MAFFT v7.187. Unambiguously aligned sequences composed of 1,023 nucleotides were selected by Gblocks and manual inspection. Maximum-likelihood (ML) tree was inferred by IQ-TREE 1.5.5 using TPM2u+I+G4 model is shown. The number of bootstrap pseudoreplicate trees was 1,000. ML tree was visualized using FigTree 1.4.0 and Keynote 6.6.2. Note that major clades supported in Fig 2 are also supported in this analysis.
(PDF)

S3 Fig SSU rDNA-based cladogram of major eukaryotic supergroups including representative cockroach-derived *Entamoeba* using different substitution model. In order to ensure consistency of the result shown in Fig 3, the phylogenetic tree was constructed with other model.m SSU rDNA sequences were aligned using MAFFT v7.187. Unambiguously aligned sequences composed of 914 nucleotides were selected by Gblocks and manual inspection. Maximum-likelihood (ML) tree was inferred by IQ-TREE 1.5.5 using TIM2+I+G4 model. The number of bootstrap pseudoreplicate trees was 1,000. ML tree was visualized as a cladogram using FigTree 1.4.0 and Keynote 6.6.2. The phylogenetic relationships of *Entamoeba* and cockroach amoebae in resultant tree are consistent with the tree in Fig 3, although some of Amoebozoa species are miss branched (Red rectangle).
(PDF)

S4 Fig Phylogenetic tree of SSU rDNA of Group C sequences of cockroach-derived *Entamoeba* using different substitution model. SSU rDNA sequences were aligned using MAFFT v7.187. Unambiguously aligned sequences composed of 1,224 nucleotides were selected by Gblocks and manual inspection. Maximum-likelihood (ML) tree was inferred by IQ-TREE 1.5.5 using HKY+I+G4 model. The number of bootstrap pseudoreplicate trees was 1,000. ML tree was visualized using FigTree 1.4.0 and Keynote 6.6.2. Bootstrap values for major nodes are shown on each node. Major clades discovered in Fig 4 were successfully reproduced.
(PDF)

S5 Fig Phylogenetic tree of SSU rDNA of representative cockroach-derived *Entamoeba* ribosomal lineages and other Archamoebae species using different substitution model. SSU rDNA sequences were aligned using MAFFT v7.187. Well-aligned 1,224 nucleotide positions were selected by Gblocks and manual operation. Maximum-likelihood (ML) tree was inferred by IQ-TREE 1.5.5 using TIM2+I+G4 model. The number of bootstrap pseudoreplicate trees was 1,000. ML tree was visualized using FigTree 1.4.0 and Keynote 6.6.2. The topology of resultant tree are consistent with the tree in Fig 5.
(PDF)

S6 Fig Multiple alignment using full length sequences of group C. SSU rDNA sequences were aligned using MAFFT v7.187. The whole part of the alignment was visualized by SeaView4. The alignment indicates exact address of well aligned sites and variant sites.
(PDF)
