# Correction: Genetic diversity of *Entamoeba*: Novel ribosomal lineages from cockroaches

**DOI:** 10.1371/journal.pone.0217215

**Published:** 2019-05-30

**Authors:** Tetsuro Kawano, Mihoko Imada, Pennapa Chamavit, Seiki Kobayashi, Tetsuo Hashimoto, Tomoyoshi Nozaki

The authors acknowledge that a closely related study was published in a 2015 Master’s thesis [1], and this work should have been cited and discussed in the article [2].

It was previously reported that *Entamoeba*-like species were identified from cockroaches [1]; this previous work investigated the diversity of *Entamoeba*-like species found in cockroaches and similarly found them to group into nine distinct clades. However, no sequence information was available. Hence, the authors decided to initiate a new investigation to verify the claims in [1] and also to provide nucleotide sequence information.

In [1], it appears that all groups were composed of multiple cockroach species. In contrast, our tree shows several clades are *P*. *americana* specific (B, E, F, and G), while others contain the *Entamoeba* lineages from both cockroach species. Especially, the largest group C abundantly contains *Entamoeba* lineages from all cockroach species in our study, which is in good contrast to the previous study. The previous report [1] showed the monophyly of *Entamoeba*-like sequences identified from *P*. *americana*. Here, we included two additional cockroach species to broaden species coverage.

The following is added to the Acknowledgements section: Although it remained unpublished, the existence of *Entamoeba*-like species in cockroaches was reported at a few conferences (ISOP-PSA Meeting, Seattle, USA, in 2011; ISOP-ISEP Conference, Oslo, Norway, in 2012; and ICOP in Vancouver in 2013). The previous studies, although unpublished, surely inspired the authors to initiate our investigation.

In the “DNA extraction and amplification of SSU rDNA derived from Entamoeba” subsection of the Material and methods, an incorrect primer sequence is provided for the second round of PCR. The correct sequence for 01R is:

5’- AAGGAGAAGTCGTAACAAGG-3’
