# Supplementary figures and images for: Genetic diversity of Entamoeba: Novel ribosomal lineages from cockroaches
Source: PLoS One. 2017 Sep 21;12(9):e0185233. doi: 10.1371/journal.pone.0185233 (PMC5608334; doi:10.1371/journal.pone.0185233)

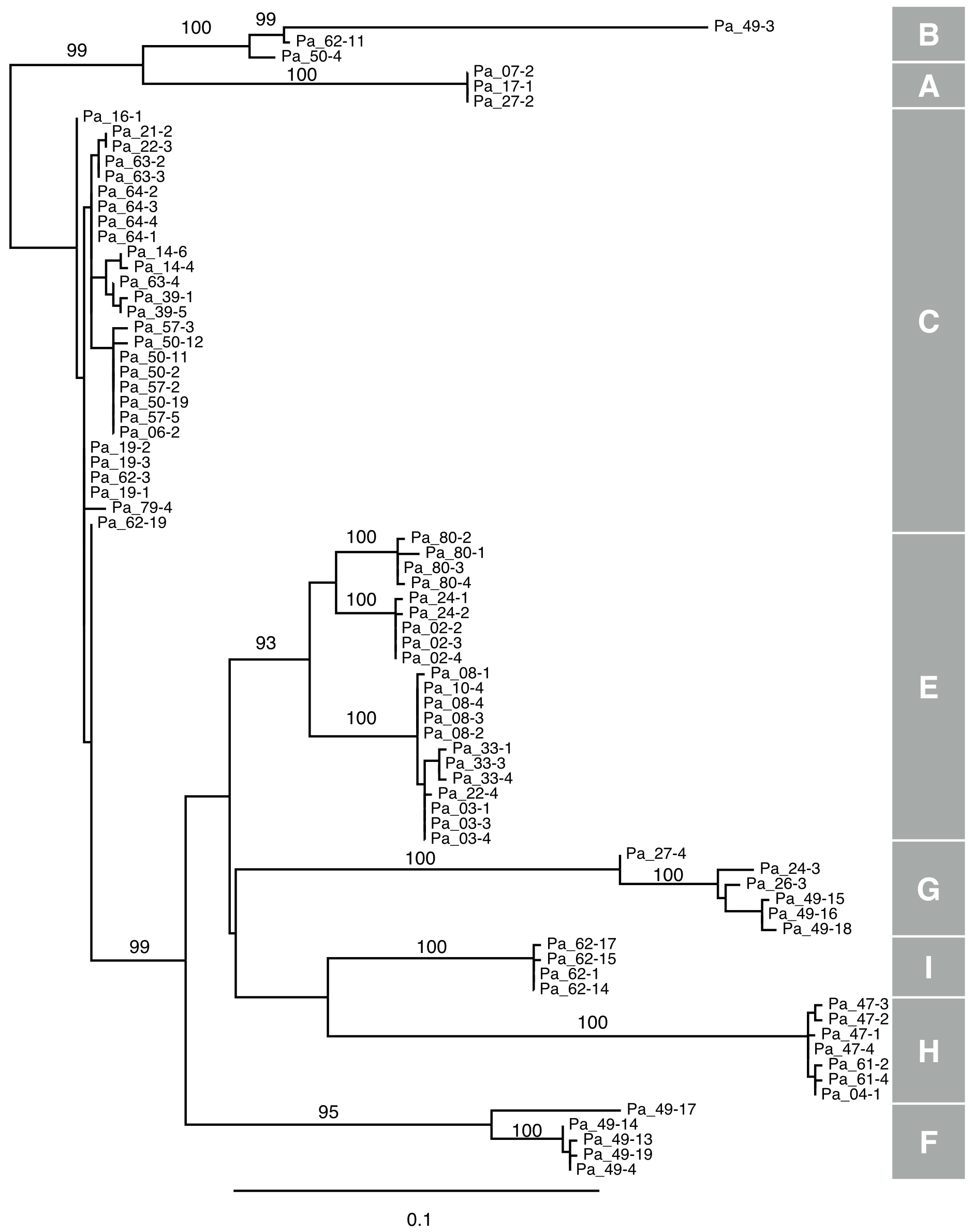

Supplement: S1 Fig — SSU rDNA sequences were aligned using MAFFT v7.187. Unambiguously aligned sequences composed of 1069 nucleotides were selected by Gblocks and manual inspection. Maximum-likelihood (ML) tree was inferred by RAxML 8.1.17 using GTRGAMMA model. The number of bootstrap pseudoreplicate trees was 100. ML tree was visualized using FigTree 1.4.0 and Keynote 6.6.2. Bootstrap values for major nodes are shown on each node. Nine groups (A-I) were shown to be monophyletic with high bootstrap support values. (TIF) [file pone.0185233.s001.tif]

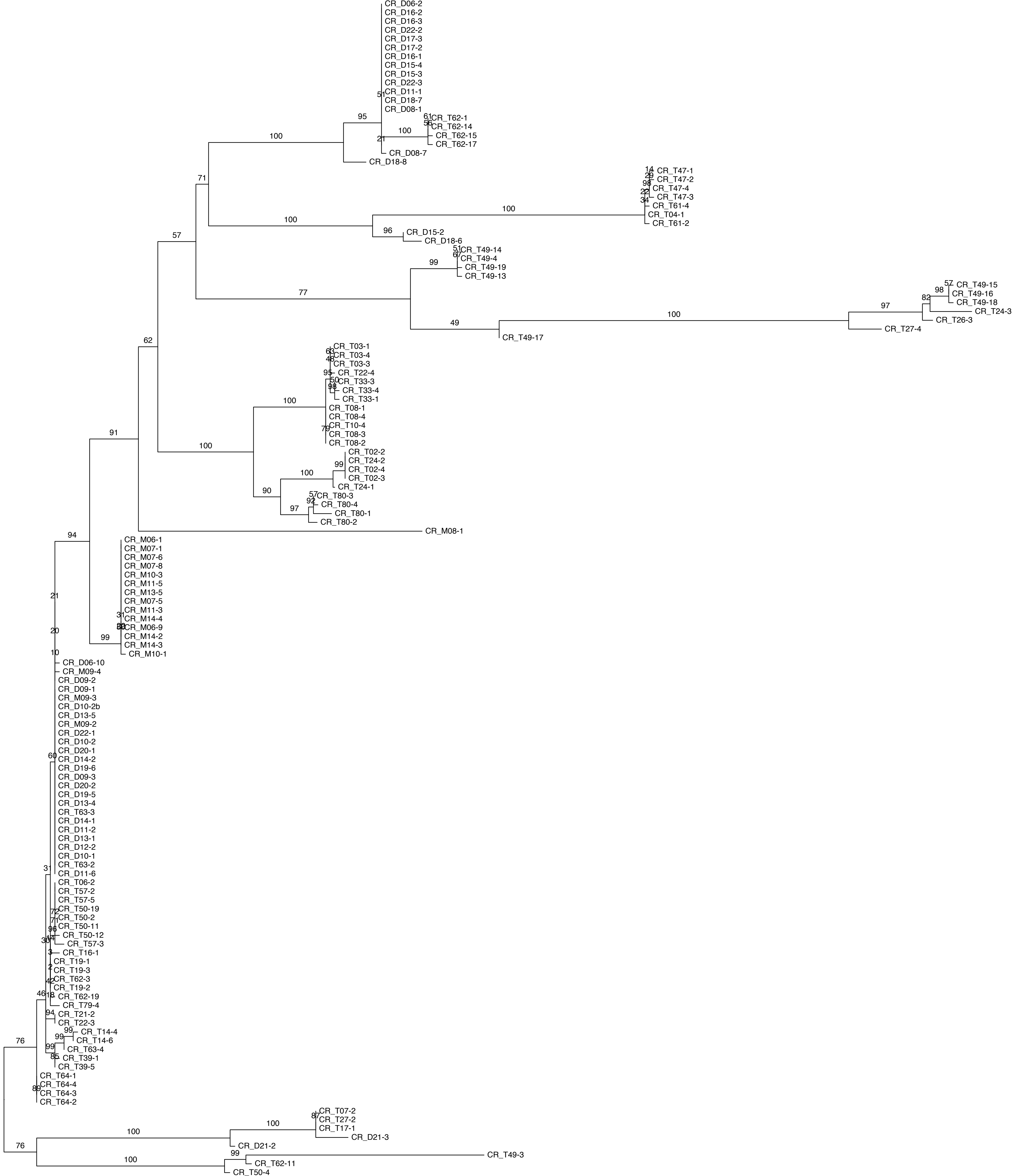

0.03

Supplement: S2 Fig — In order to ensure consistency of the result shown in Fig 2, the phylogenetic tree was constructed with other model. SSU rDNA sequences were aligned using MAFFT v7.187. Unambiguously aligned sequences composed of 1,023 nucleotides were selected by Gblocks and manual inspection. Maximum-likelihood (ML) tree was inferred by IQ-TREE 1.5.5 using TPM2u+I+G4 model is shown. The number of bootstrap pseudoreplicate trees was 1,000. ML tree was visualized using FigTree 1.4.0 and Keynote 6.6.2. Note that major clades supported in Fig 2 are also supported in this analysis. (PDF) [file pone.0185233.s002.pdf]

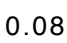

Supplement: S3 Fig — In order to ensure consistency of the result shown in Fig 3, the phylogenetic tree was constructed with other model.m SSU rDNA sequences were aligned using MAFFT v7.187. Unambiguously aligned sequences composed of 914 nucleotides were selected by Gblocks and manual inspection. Maximum-likelihood (ML) tree was inferred by IQ-TREE 1.5.5 using TIM2+I+G4 model. The number of bootstrap pseudoreplicate trees was 1,000. ML tree was visualized as a cladogram using FigTree 1.4.0 and Keynote 6.6.2. The phylogenetic relationships of Entamoeba and cockroach amoebae in resultant tree are consistent with the tree in Fig 3, although some of Amoebozoa species are miss branched (Red rectangle). (PDF) [file pone.0185233.s003.pdf]

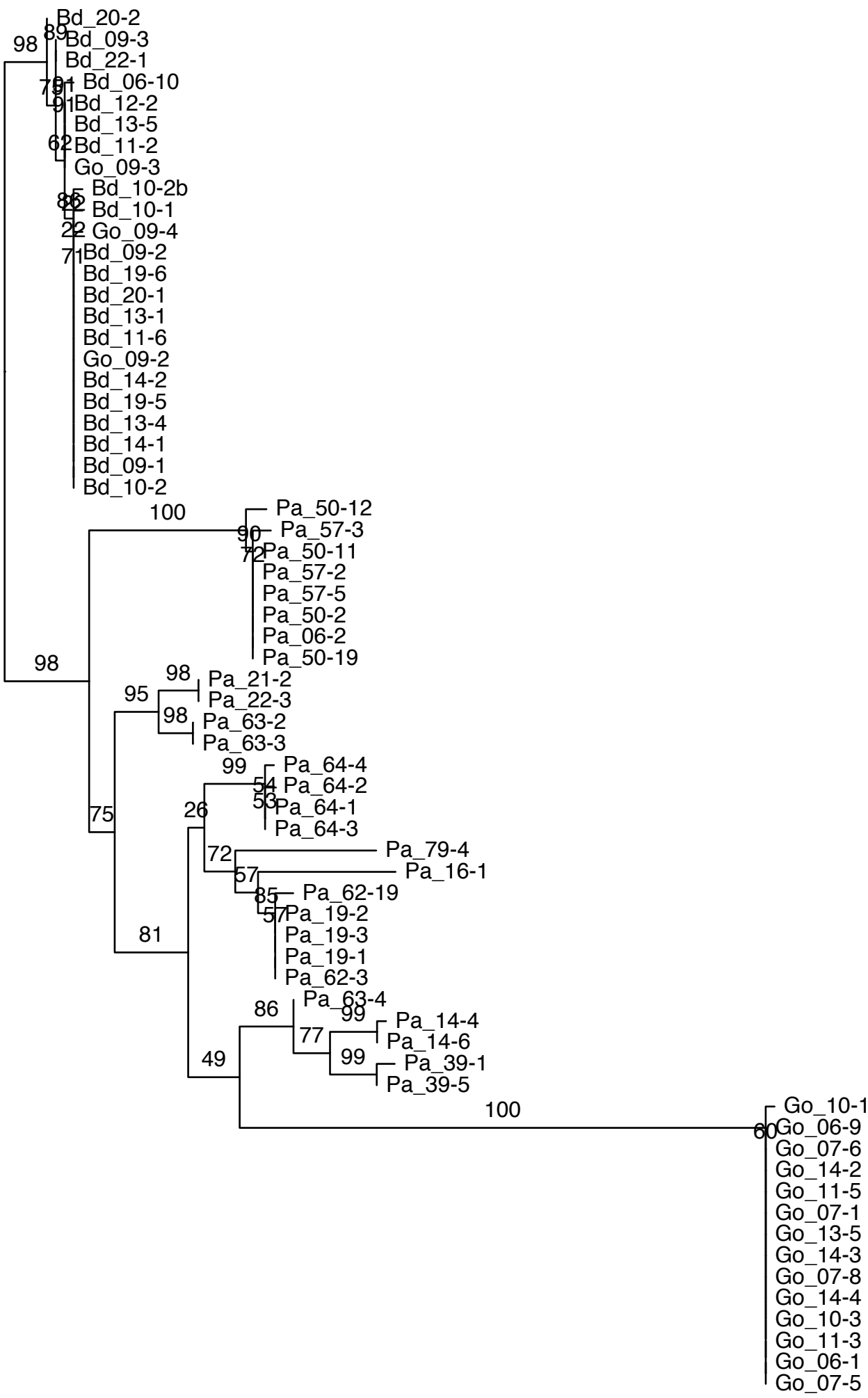

0.0070

Supplement: S4 Fig — SSU rDNA sequences were aligned using MAFFT v7.187. Unambiguously aligned sequences composed of 1,224 nucleotides were selected by Gblocks and manual inspection. Maximum-likelihood (ML) tree was inferred by IQ-TREE 1.5.5 using HKY+I+G4 model. The number of bootstrap pseudoreplicate trees was 1,000. ML tree was visualized using FigTree 1.4.0 and Keynote 6.6.2. Bootstrap values for major nodes are shown on each node. Major clades discovered in Fig 4 were successfully reproduced. (PDF) [file pone.0185233.s004.pdf]

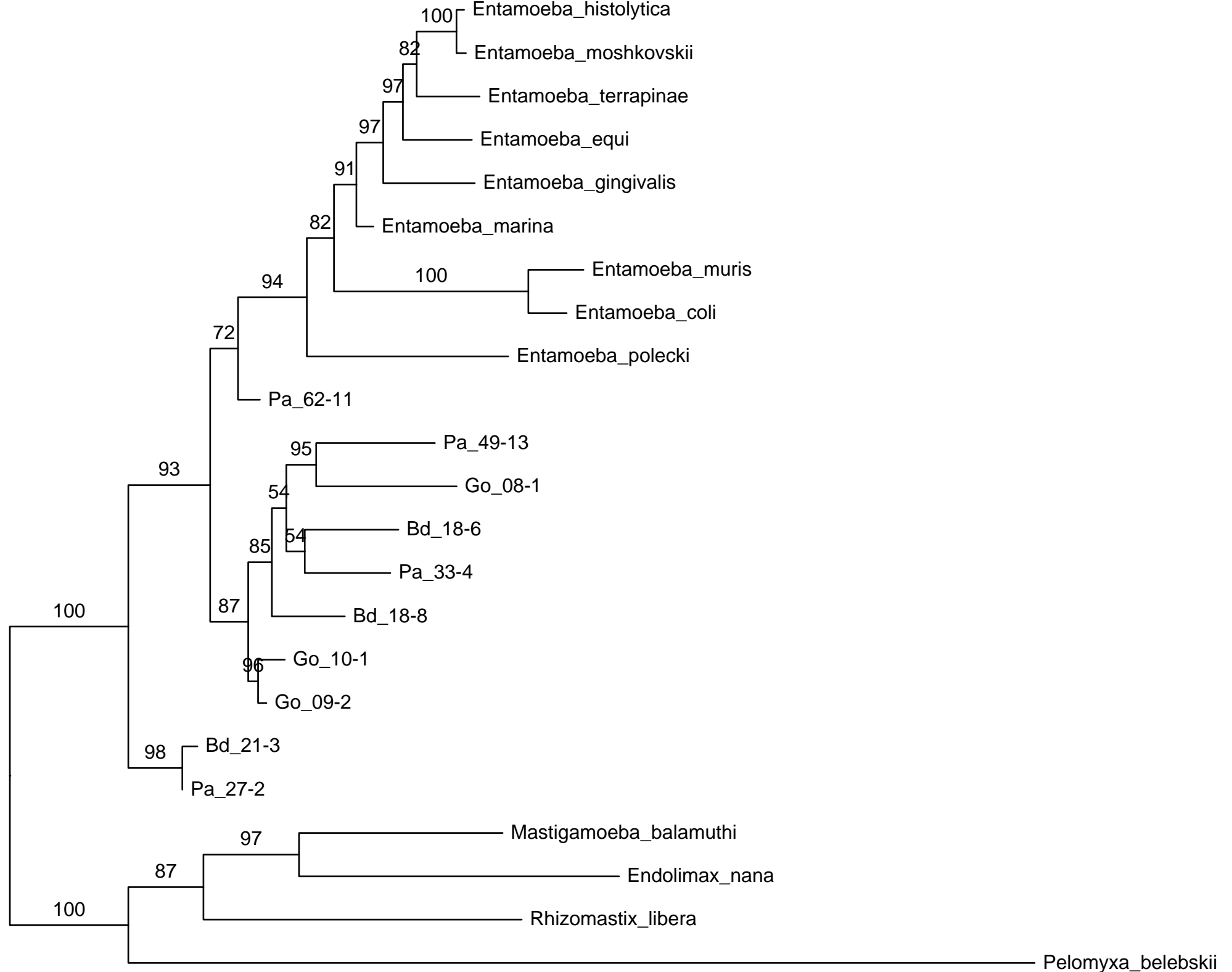

0.05

Supplement: S5 Fig — SSU rDNA sequences were aligned using MAFFT v7.187. Well-aligned 1,224 nucleotide positions were selected by Gblocks and manual operation. Maximum-likelihood (ML) tree was inferred by IQ-TREE 1.5.5 using TIM2+I+G4 model. The number of bootstrap pseudoreplicate trees was 1,000. ML tree was visualized using FigTree 1.4.0 and Keynote 6.6.2. The topology of resultant tree are consistent with the tree in Fig 5. (PDF) [file pone.0185233.s005.pdf]
