# Supplementary material for: Genetic diversity of Entamoeba: Novel ribosomal lineages from cockroaches
Source: PLoS One. 2017 Sep 21;12(9):e0185233. doi: 10.1371/journal.pone.0185233 (PMC5608334; doi:10.1371/journal.pone.0185233)
Supplement: S6 Fig — SSU rDNA sequences were aligned using MAFFT v7.187. The whole part of the alignment was visualized by SeaView4. The alignment indicates exact address of well aligned sites and variant sites. (PDF) [file pone.0185233.s006.pdf]

```
             1
Bd_06-10     TGTTGAAGAT TAAGCCATGC ATGTGTAAGT ATAAAGACCT AGTAGGATGA AACTGCGGAC GGCTCATTAC
Bd_09-1      TGTTGAAGAT TAAGCCATGC ATGTGTAAGT ATAAAGACCT AGTAGGATGA AACTGCGGAC GGCTCATTAC
Bd_09-2      TGTTGAAGAT TAAGCCATGC ATGTGTAAGT ATAAAGACCT AGTAGGATGA AACTGCGGAC GGCTCATTAC
Bd_09-3      TGTTGAAGAT TAAGCCATGC ATGTGTAAGT ATAAAGACCT AGTAGGATGA AACTGCGGAC GGCTCATTAC
Bd_10-1      TGTTGAAGAT TAAGCCATGC ATGTGTAAGT ATAAAGACCT AGTAGGATGA AACTGCGGAC GGCTCATTAC
Bd_10-2      TGTTGAAGAT TAAGCCATGC ATGTGTAAGT ATAAAGACCT AGTAGGATGA AACTGCGGAC GGCTCATTAC
Bd_10-2b     TGTTGAAGAT TAAGCCATGC ATGTGTAAGT ATAAAGACCT AGTAGGATGA AACTGCGGAC GGCTCATTAC
Bd_11-2      TGTTGAAGAT TAAGCCATGC ATGTGTAAGT ATAAAGACCT AGTAGGATGA AACTGCGGAC GGCTCATTAC
Bd_11-6      TGTTGAAGAT TAAGCCATGC ATGTGTAAGT ATAAAGACCT AGTAGGATGA AACTGCGGAC GGCTCATTAC
Bd_12-2      TGTTGAAGAT TAAGCCATGC ATGTGTAAGT ATAAAGACCT AGTAGGATGA AACTGCGGAC GGCTCATTAC
Bd_13-1      TGTTGAAGAT TAAGCCATGC ATGTGTAAGT ATAAAGACCT AGTAGGATGA AACTGCGGAC GGCTCATTAC
Bd_13-4      TGTTGAAGAT TAAGCCATGC ATGTGTAAGT ATAAAGACCT AGTAGGATGA AACTGCGGAC GGCTCATTAC
Bd_13-5      TGTTGAAGAT TAAGCCATGC ATGTGTAAGT ATAAAGACCT AGTAGGATGA AACTGCGGAC GGCTCATTAC
Bd_14-1      TGTTGAAGAT TAAGCCATGC ATGTGTAAGT ATAAAGACCT AGTAGGATGA AACTGCGGAC GGCTCATTAC
Bd_14-2      TGTTGAAGAT TAAGCCATGC ATGTGTAAGT ATAAAGACCT AGTAGGATGA AACTGCGGAC GGCTCATTAC
Bd_19-5      TGTTGAAGAT TAAGCCATGC ATGTGTAAGT ATAAAGACCT AGTAGGATGA AACTGCGGAC GGCTCATTAC
Bd_19-6      TGTTGAAGAT TAAGCCATGC ATGTGTAAGT ATAAAGACCT AGTAGGATGA AACTGCGGAC GGCTCATTAC
Bd_20-1      TGTTGAAGAT TAAGCCATGC ATGTGTAAGT ATAAAGACCT AGTAGGATGA AACTGCGGAC GGCTCATTAC
Bd_20-2      TGTTGAAGAT TAAGCCATGC ATGTGTAAGT ATAAAGACCT AGTAGGATGA AACTGCGGAC GGCTCATTAC
Bd_22-1      TGTTGAAGAT TAAGCCATGC ATGTGTAAGT ATAAAGACCT AGTAGGATGA AACTGCGGAC GGCTCATTAC
Go_06-1      TGTTGAAGAT TAAGCCATGC ATGTGTAAGT ATAAAGACCT AGAAGGATGA AACTGCGGAC GGCTCATTAC
Go_06-9      TGTTGAAGAT TAAGCCATGC ATGTGTAAGT ATAAAGACCT AGAAGGATGA AACTGCGGAC GGCTCATTAC
Go_07-1      TGTTGAAGAT TAAGCCATGC ATGTGTAAGT ATAAAGACCT AGAAGGATGA AACTGCGGAC GGCTCATTAC
Go_07-5      TGTTGAAGAT TAAGCCATGC ATGTGTAAGT ATAAAGACCT AGAAGGATGA AACTGCGGAC GGCTCATTAC
Go_07-6      TGTTGAAGAT TAAGCCATGC ATGTGTAAGT ATAAAGACCT AGAAGGATGA AACTGCGGAC GGCTCATTAC
Go_07-8      TGTTGAAGAT TAAGCCATGC ATGTGTAAGT ATAAAGACCT AGAAGGATGA AACTGCGGAC GGCTCATTAC
Go_09-2      TGTTGAAGAT TAAGCCATGC ATGTGTAAGT ATAAAGACCT AGTAGGATGA AACTGCGGAC GGCTCATTAC
Go_09-3      TGTTGAAGAT TAAGCCATGC ATGTGTAAGT ATAAAGACCT AGTAGGATGA AACTGCGGAC GGCTCATTAC
Go_09-4      TGTTGAAGAT TAAGCCATGC ATGTGTAAGT ATAAAGACCT AGTAGGATGA AACTGCGGAC GGCTCATTAC
Go_10-1      TGTTGAAGAT TAAGCCATGC ATGTGTAAGT ATAAAGACCT AGAAGGATGA AACTGCGGAC GGCTCATTAC
Go_10-3      TGTTGAAGAT TAAGCCATGC ATGTGTAAGT ATAAAGACCT AGAAGGATGA AACTGCGGAC GGCTCATTAC
Go_11-3      TGTTGAAGAT TAAGCCATGC ATGTGTAAGT ATAAAGACCT AGAAGGATGA AACTGCGGAC GGCTCATTAC
Go_11-5      TGTTGAAGAT TAAGCCATGC ATGTGTAAGT ATAAAGACCT AGAAGGATGA AACTGCGGAC GGCTCATTAC
Go_13-5      TGTTGAAGAT TAAGCCATGC ATGTGTAAGT ATAAAGACCT AGAAGGATGA AACTGCGGAC GGCTCATTAC
Go_14-2      TGTTGAAGAT TAAGCCATGC ATGTGTAAGT ATAAAGACCT AGAAGGATGA AACTGCGGAC GGCTCATTAC
Go_14-3      TGTTGAAGAT TAAGCCATGC ATGTGTAAGT ATAAAGACCT AGAAGGATGA AACTGCGGAC GGCTCATTAC
Go_14-4      TGTTGAAGAT TAAGCCATGC ATGTGTAAGT ATAAAGACCT AGAAGGATGA AACTGCGGAC GGCTCATTAC
Pa_06-2      TGTTGAAGAT TAAGCCATGC ATGTGTAAGT ATAAAGACCT AGTAGGATGA AACTGCGGAC GGCTCATTAC
Pa_14-4      TGTTGAAGAT TAAGCCATGC ATGTGTAAGT ATAAAGACCT AGTAGGATGA AACTGCGGAC GGCTCATTAC
Pa_14-6      TGTTGAAGAT TAAGCCATGC ATGTGTAAGT ATAAAGACCT AGTAGGATGA AACTGCGGAC GGCTCATTAC
Pa_16-1      TGTTGAAGAT TAAGCCATGC ATGTGTAAGT ATAAAGACCT AGTAGGATGA AACTGCGGAC GGCTCATTAC
Pa_19-1      TGTTGAAGAT TAAGCCATGC ATGTGTAAGT ATAAAGACCT AGTAGGATGA AACTGCGGAC GGCTCATTAC
Pa_19-2      TGTTGAAGAT TAAGCCATGC ATGTGTAAGT ATAAAGACCT AGTAGGATGA AACTGCGGAC GGCTCATTAC
Pa_19-3      TGTTGAAGAT TAAGCCATGC ATGTGTAAGT ATAAAGACCT AGTAGGATGA AACTGCGGAC GGCTCATTAC
Pa_21-2      TGTTGAAGAT TAAGCCATGC ATGTGTAAGT ATAAAGACCT AGTAGGATGA AACTGCGGAC GGCTCATTAC
Pa_22-3      TGTTGAAGAT TAAGCCATGC ATGTGTAAGT ATAAAGACCT AGTAGGATGA AACTGCGGAC GGCTCATTAC
Pa_39-1      TGTTGAAGAT TAAGCCATGC ATGTGTAAGT ATAAAGACCT AGTAGGATGA AACTGCGGAC GGCTCATTAC
Pa_39-5      TGTTGAAGAT TAAGCCATGC ATGTGTAAGT ATAAAGACCT AGTAGGATGA AACTGCGGAC GGCTCATTAC
Pa_50-11     TGTTGAAGAT TAAGCCATGC ATGTGTAAGT ATAAAGACCT AGTAGGATGA AACTGCGGAC GGCTCATTAC
Pa_50-12     TGTTGAAGAT TAAGCCATGC ATGTGTAAGT ATAAAGACCT AGTAGGATGA AACTGCGGAC GGCTCATTAC
Pa_50-19     TGTTGAAGAT TAAGCCATGC ATGTGTAAGT ATAAAGACCT AGTAGGATGA AACTGCGGAC GGCTCATTAC
Pa_50-2      TGTTGAAGAT TAAGCCATGC ATGTGTAAGT ATAAAGACCT AGTAGGATGA AACTGCGGAC GGCTCATTAC
Pa_57-2      TGTTGAAGAT TAAGCCATGC ATGTGTAAGT ATAAAGACCT AGTAGGATGA AACTGCGGAC GGCTCATTAC
Pa_57-3      TGTTGAAGAT TAAGCCATGC ATGTGTAAGT ATAAAGACCT AGTAGGATGA AACTGCGGAC GGCTCATTAC
Pa_57-5      TGTTGAAGAT TAAGCCATGC ATGTGTAAGT ATAAAGACCT AGTAGGATGA AACTGCGGAC GGCTCATTAC
Pa_62-19     TGTTAAAGAT TAAGCCATGC ATGTGTAAGT ATAAAGACCA AGTAGGATGA AACTGCGGAC GGCTCATTAC
Pa_62-3      TGTTGAAGAT TAAGCCATGC ATGTGTAAGT ATAAAGACCT AGTAGGATGA AACTGCGGAC GGCTCATTAC
Pa_63-2      TGTTGAAGAT TAAGCCATGC ATGTGTAAGT ATAAAGACCT AGTAGGATGA AACTGCGGAC GGCTCATTAC
Pa_63-3      TGTTGAAGAT TAAGCCATGC ATGTGTAAGT ATAAAGACCT AGTAGGATGA AACTGCGGAC GGCTCATTAC
Pa_63-4      TGTTGAAGAT TAAGCCATGC ATGTGTAAGT ATAAAGACCT AGTAGGATGA AACTGCGGAC GGCTCATTAC
Pa_64-1      TGTTGAAGAT TAAGCCATGC ATGTGTAAGT ATAAAGACCT AGTAGGATGA AACTGCGGAC GGCTCATTAC
Pa_64-2      TGTTGAAGAT TAAGCCATGC ATGTGTAAGT ATAAAGACCT AGTAGGATGA AACTGCGGAC GGCTCATTAC
Pa_64-3      TGTTGAAGAT TAAGCCATGC ATGTGTAAGT ATAAAGACCT AGTAGGATGA AACTGCGGAC GGCTCATTAC
Pa_64-4      TGTTGAAGAT TAAGCCATGC ATGTGTAAGT ATAAAGACCT AGTAGGATGA AACTGCGGAC GGCTCATTAC
Pa_79-4      TGTTGAAGAT TAAGCCATGC ATGTGTAAGT ATAAAGACAT AGTAGGATGA AACTGCGGAC GGCTCATTAC
```

```
             71
Bd_06-10     AACAGTTATA GTTTCTTTGG TTAGTATCAA AAGGATAACT CTGTTAATTG TAGAGCTAAT ACTTGAGACA
Bd_09-1      AACAGTTATA GTTTCTTTGG TTAGTATCAA AAGGATAACT CTGTTAATTG TAGAGCTAAT ACTTGAGACA
Bd_09-2      AACAGTTATA GTTTCTTTGG TTAGTATCAA AAGGATAACT CTGTTAATTG TAGAGCTAAT ACTTGAGACA
Bd_09-3      AACAGTTATA GTTTCTTTGG TTAGTATCAA AAGGATAACT CTGTTAATTG TAGAGCTAAT ACTTGAGACA
Bd_10-1      AACAGTTATA GTTTCTTTGG TTAGTATCAA AAGGATAACT CTGTTAATTG TAGAGCTAAT ACTTGAGACA
Bd_10-2      AACAGTTATA GTTTCTTTGG TTAGTATCAA AAGGATAACT CTGTTAATTG TAGAGCTAAT ACTTGAGACA
Bd_10-2b     AACAGTTATA GTTTCTTTGG TTAGTATCAA AAGGATAACT CTGTTAATTG TAGAGCTAAT ACTTGAGACA
Bd_11-2      AACAGTTATA GTTTCTTTGG TTAGTATCAA AAGGATAACT CTGTTAATTG TAGAGCTAAT ACTTGAGACA
Bd_11-6      AACAGTTATA GTTTCTTTGG TTAGTATCAA AAGGATAACT CTGTTAATTG TAGAGCTAAT ACTTGAGACA
Bd_12-2      AACAGTTATA GTTTCTTTGG TTAGTATCAA AAGGATAACT CTGTTAATTG TAGAGCTAAT ACTTGAGACA
Bd_13-1      AACAGTTATA GTTTCTTTGG TTAGTATCAA AAGGATAACT CTGTTAATTG TAGAGCTAAT ACTTGAGACA
Bd_13-4      AACAGTTATA GTTTCTTTGG TTAGTATCAA AAGGATAACT CTGTTAATTG TAGAGCTAAT ACTTGAGACA
Bd_13-5      AACAGTTATA GTTTCTTTGG TTAGTATCAA AAGGATAACT CTGTTAATTG TAGAGCTAAT ACTTGAGACA
Bd_14-1      AACAGTTATA GTTTCTTTGG TTAGTATCAA AAGGATAACT CTGTTAATTG TAGAGCTAAT ACTTGAGACA
Bd_14-2      AACAGTTATA GTTTCTTTGG TTAGTATCAA AAGGATAACT CTGTTAATTG TAGAGCTAAT ACTTGAGACA
Bd_19-5      AACAGTTATA GTTTCTTTGG TTAGTATCAA AAGGATAACT CTGTTAATTG TAGAGCTAAT ACTTGAGACA
Bd_19-6      AACAGTTATA GTTTCTTTGG TTAGTATCAA AAGGATAACT CTGTTAATTG TAGAGCTAAT ACTTGAGACA
Bd_20-1      AACAGTTATA GTTTCTTTGG TTAGTATCAA AAGGATAACT CTGTTAATTG TAGAGCTAAT ACTTGAGACA
Bd_20-2      AACAGTTATA GTTTCTTTGG TTAGTATCAA AAGGATAACT CTGTTAATTG TAGAGCTAAT ACTTGAGACA
Bd_22-1      AACAGTTATA GTTTCTTTGG TTAGTATCAA AAGGATAACT CTGTTAATTG TAGAGCTAAT ACTTGAGACA
Go_06-1      AACAGTTATA GTTTCTTTGG TTAGTAGTAA AAGGATAACT CTGTTAATTG TAGAGCTAAT ACTTGAGACT
Go_06-9      AACAGTTATA GTTTCTTTGG TTAGTAGTAA AAGGATAACT CTGTTAATTG TAGAGCTAAT ACTTGAGACT
Go_07-1      AACAGTTATA GTTTCTTTGG TTAGTAGTAA AAGGATAACT CTGTTAATTG TAGAGCTAAT ACTTGAGACT
Go_07-5      AACAGTTATA GTTTCTTTGG TTAGTAGTAA AAGGATAACT CTGTTAATTG TAGAGCTAAT ACTTGAGACT
Go_07-6      AACAGTTATA GTTTCTTTGG TTAGTAGTAA AAGGATAACT CTGTTAATTG TAGAGCTAAT ACTTGAGACT
Go_07-8      AACAGTTATA GTTTCTTTGG TTAGTAGTAA AAGGATAACT CTGTTAATTG TAGAGCTAAT ACTTGAGACT
Go_09-2      AACAGTTATA GTTTCTTTGG TTAGTATCAA AAGGATAACT CTGTTAATTG TAGAGCTAAT ACTTGAGACA
Go_09-3      AACAGTTATA GTTTCTTTGG TTAGTATCAA AAGGATAACT CTGTTAATTG TAGAGCTAAT ACTTGAGACA
Go_09-4      AACAGTTATA GTTTCTTTGG TTAGTATCAA AAGGATAACT CTGTTAATTG TAGAGCTAAT ACTTGAGACA
Go_10-1      AACAGTTATA GTTTCTTTGG TTAGTAGTAA AAGGATAACT CCGTTAATTG TAGAGCTAAT ACTTGAGACT
Go_10-3      AACAGTTATA GTTTCTTTGG TTAGTAGTAA AAGGATAACT CTGTTAATTG TAGAGCTAAT ACTTGAGACT
Go_11-3      AACAGTTATA GTTTCTTTGG TTAGTAGTAA AAGGATAACT CTGTTAATTG TAGAGCTAAT ACTTGAGACT
Go_11-5      AACAGTTATA GTTTCTTTGG TTAGTAGTAA AAGGATAACT CTGTTAATTG TAGAGCTAAT ACTTGAGACT
Go_13-5      AACAGTTATA GTTTCTTTGG TTAGTAGTAA AAGGATAACT CTGTTAATTG TAGAGCTAAT ACTTGAGACT
Go_14-2      AACAGTTATA GTTTCTTTGG TTAGTAGTAA AAGGATAACT CTGTTAATTG TAGAGCTAAT ACTTGAGACT
Go_14-3      AACAGTTATA GTTTCTTTGG TTAGTAGTAA AAGGATAACT CTGTTAATTG TAGAGCTAAT ACTTGAGACT
Go_14-4      AACAGTTATA GTTTCTTTGG TTAGTAGTAA AAGGATAACT CTGTTAATTG TAGAGCTAAT ACTTGAGACT
Pa_06-2      AACAGTTATA GTTTCTTTGG TTAGTACAAA AAGGATAACT CTGTTAATTG TAGAGCTAAT ACTTGAGACT
Pa_14-4      AACAGTTATA GTTTCTTTGG TTAGTAATAA AATGATAACT CTGTTAATTG TAGAGCTAAT ACTTGAGACT
Pa_14-6      AACAGTTATA GTTTCTTTGG TTAGTAATAA AATGATAACT CTGTTAATTG TAGAGCTAAT ACTTGAGACT
Pa_16-1      AACAGTTATA GTTTCTTTGG TTAGTACGAA AAGGATAACT CTGTTAATTG TAGAGCTAAT ACTTGAGACA
Pa_19-1      AACAGTTATA GTTTCTTTGG TTAGTACGAA AAGGATAACT CTGTTAATTG TAGAGCTAAT ACTTGAGACA
Pa_19-2      AACAGTTATA GTTTCTTTGG TTAGTACGAA AAGGATAACT CTGTTAATTG TAGAGCTAAT ACTTGAGACA
Pa_19-3      AACAGTTATA GTTTCTTTGG TTAGTACGAA AAGGATAACT CTGTTAATTG TAGAGCTAAT ACTTGAGACA
Pa_21-2      AACAGTTATA GTTTCTTTGG TTAGTAACAA AAGGATAACT CTGTTAATTG TAGAGCTAAT ACTTGAGACT
Pa_22-3      AACAGTTATA GTTTCTTTGG TTAGTAACAA AAGGATAACT CTGTTAATTG TAGAGCTAAT ACTTGAGACT
Pa_39-1      AACAGTTATA GTTTCTTTGG TTAGTAGTAA AAGGATAACT CTGTTAATTG TAGAGCTAAT ACTTGAGACA
Pa_39-5      AACAGTTATA GTTTCTTTGG TTAGTAGTAA AAGGATAACT CTGTTAATTG TAGAGCTAAT ACTTGAGACA
Pa_50-11     AACAGTTATA GTTTCTTTGG TTAGTACAAA AAGGATAACT CTGTTAATTG TAGAGCTAAT ACTTGAGACT
Pa_50-12     AACAGTTATA GTTTCTTTGG TTAGTACAAA AAGGATAACT CTGTTAATTG TAGAGCTAAT ACTTGAGACT
Pa_50-19     AACAGTTATA GTTTCTTTGG TTAGTACAAA AAGGATAACT CTGTTAATTG TAGAGCTAAT ACTTGAGACT
Pa_50-2      AACAGTTATA GTTTCTTTGG TTAGTACAAA AAGGATAACT CTGTTAATTG TAGAGCTAAT ACTTGAGACT
Pa_57-2      AACAGTTATA GTTTCTTTGG TTAGTACAAA AAGGATAACT CTGTTAATTG TAGAGCTAAT ACTTGAGACT
Pa_57-3      AACAGTTATA GTTTCTTTGG TTAGTACAAA AAGGATAACT CTGTTAATTG TAGAGCTAAT ACTTGAGACT
Pa_57-5      AACAGTTATA GTTTCTTTGG TTAGTACAAA AAGGATAACT CTGTTAATTG TAGAGCTAAT ACTTGAGACT
Pa_62-19     AACAGTTATA GTTTCTTTGG TTAGTACGAA AAGGATAACT CTGTTAATTG TAGAGCTAAT ACTTGAGACA
Pa_62-3      AACAGTTATA GTTTCTTTGG TTAGTACGAA AAGGATAACT CTGTTAATTG TAGAGCTAAT ACTTGAGACA
Pa_63-2      AACAGTTATA GTTTCTTTGG TTAGTAATAA AAGGATAACT CTGTTAATTG TAGAGCTAAT ACTTGAGACT
Pa_63-3      AACAGTTATA GTTTCTTTGG TTAGTAATAA AAGGATAACT CTGTTAATTG TAGAGCTAAT ACTTGAGACT
Pa_63-4      AACAGTTATA GTTTCTTTGG TTAGTAATAA AAGGATAACT CTGTTAATTG TAGAGCTAAT ACTTGAGACA
Pa_64-1      AACAGTTATA GTTTCTTTGG TTAGTACGAA AAGGATAACT CTGTTAATTG TAGAGCTAAT ACTTGAGACG
Pa_64-2      AACAGTTATA GTTTCTTTGG TTAGTACGAA AAGGATAACT CTGTTAATTG TAGAGCTAAT ACTTGAGACG
Pa_64-3      AACAGTTATA GTTTCTTTGG TTAGTACGAA AAGGATAACT CTGTTAATTG TAGAGCTAAT ACTTGAGACG
Pa_64-4      AACAGTTATA GTTTCTTTGG TTAGTACGAA AAGGATAACT CTGTTAATTG TAGAGCTAAT ACTTGAGACG
Pa_79-4      AACAGTTATA GTTTCTTTGG TTAGTAGGAA AAGGATAACT CTGTTAATTG TAGAGCTAAT ACTTGAGACA
```

```
             141
Bd_06-10     TTCCACTTTC T-TCCGAGAC TGTGGCGGCC GTACCCCGGT GCGGTTATGA ATTATTCCAT TCTGAATGCT
Bd_09-1      TTCCACTTTC T-TTCGAGAC TGTGGCGGCC GTACCCCGGT GCGGTTATGA ATTATTCCAT TCTGAATGCT
Bd_09-2      TTCCACTTTC T-TCCGAGAC TGTGGCGGCC GTACCCCGGT GCGGTTATGA ATTATTCCAT TCTGAATGCT
Bd_09-3      TTCCACTTTC T-TTCGAGAC TGTGGCGGCC GTACCCCGGT GCGGTTATGA ATTATTCCAT TCTGAATGCT
Bd_10-1      TTCCACTTTC T-TTCGAGAC TGTGGCGGCC GTACCCCGGT GCGGTTATGA ATTATTCCAT TCTGAATGCT
Bd_10-2      TTCCACTTTC T-TCCGAGAC TGTGGCGGCC GTACCCAGGT GCGGTTATGA ATTATTCCAT TCTGAATGCT
Bd_10-2b     TTCCACTTTC T-TTCGAGAC TGTGGCGGCC GTACCCCGGT GCGGTTATGA ATTATTCCAT TCTGAATGCT
Bd_11-2      TTCCACTTTC T-TTCGAGAC TGTGGCGGCC GTACCCCGGT GCGGTTATGA ATTATTCCAT TCTGAATGCT
Bd_11-6      TTCCACTTTC T-TTCGAGAC TGTGGCGGCC GTACCCCGGT GCGGTTATGA ATTATTCCAT TCTGAATGCT
Bd_12-2      TTCCACTTTC T-TCCGAGAC TGTGGCGGCC GTACCCCGGT GCGGTTATGA ATTATTCCAT TCTGAATGCT
Bd_13-1      TTCCACTTTC T-TCCGAGAC TGTGGCGGCC GTACCCAGGT GCGGTTATGA ATTATTCCAT TCTGAATGCT
Bd_13-4      TTCCACTTTC T-TCCGAGAC TGTGGCGGCC GTACCCAGGT GCGGTTATGA ATTATTCCAT TCTGAATGCT
Bd_13-5      TTCCACTTTC T-TTCGAGAC TGTGGCGGCC GTACCCCGGT GCGGTTATGA ATTATTCCAT TCTGAATGCT
Bd_14-1      TTCCACTTTC T-TCCGAGAC TGTGGCGGCC GTACCCAGGT GCGGTTATGA ATTATTCCAT TCTGAATGCT
Bd_14-2      TTCCACTTTC T-TTCGAGAC TGTGGCGGCC GTACCCCGGT GCGGTTATGA ATTATTCCAT TCTGAATGCT
Bd_19-5      TTCCACTTTC T-TTCGAGAC TGTGGCGGCC GTACCCCGGT GCGGTTATGA ATTATTCCAT TCTGAATGCT
Bd_19-6      TTCCACTTTC T-TTCGAGAC TGTGGCGGCC GTACCCAGGT GCGGTTATGA ATTATTCCAT TCTGAATGCT
Bd_20-1      TTCCACTTTC T-TCCGAGAC TGTGGCGGCC GTACCCAGGT GCGGTTATGA ATTATTCCAT TCTGAATGCT
Bd_20-2      TTCCACTTTC T-TTCGAGAC TGTGGCGGCC GTACCCCGGT GCGGTTATGA ATTATTCCAT TCTGAATGCT
Bd_22-1      TTCCACTTTC T-TCCGAGAC TGTGGCGGCC GTACCCAGGT GCGGTTATGA ATTATTCCAT TCTGAATGCT
Go_06-1      ATCCCCTGTT C-GCCAGGGC AGGGGCGACT GCACTT--GT GCAGTTATGA ATTATTCCAT GTTGAATGCT
Go_06-9      ATCCCCCGTT C-GACAGGGC AGGGGCGACT GCACTT--GT GCAGTTATGA ATTATTCCAT GTTGAATGCT
Go_07-1      ATCCCCTGTT C-GCCAGGGC AGGGGCGACT GCACTT--GT GCAGTTATGA ATTATTCCAT GTTGAATGCT
Go_07-5      ATCCCCTGTT C-GCCAGGGC AGGGGCGACT GCACTT--GT GCAGTTATGA ATTATTCCAT GTTGAATGCT
Go_07-6      ATCCCCTGTT C-GCCAGGGC AGGGGCGACT GCACTT--GT GCAGTTATGA ATTATTCCAT GTTGAATGCT
Go_07-8      ATCCCCTGTT C-GCCAGGGC AGGGGCGACT GCACTT--GT GCAGTTATGA ATTATTCCAT GTTGAATGCT
Go_09-2      TTCCACTTTC T-TTCGAGAC TGTGGCGGCC GTACCCCGGT GCGGTTATGA ATTATTCCAT TCTGAATGCT
Go_09-3      TTCCACTTTC T-TCCGAGAC TGTGGCGGCC GTACCCCGGT GCGGTTATGA ATTATTCCAT TCTGAATGCT
Go_09-4      TTCCACTTTC T-TCCGAGAC TGTGGCGGCC GTACCCAGGT GCGGTTATGA ATTATTCCAT TCTGAATGCT
Go_10-1      ATCCCCTGTT C-GACAGGGC AGGGGCGACT GCACTT--GT GCAGTTATGA ATTATTCCAT GTTGAATGCT
Go_10-3      ATCCCCTGTT C-GCCAGGGC AGGGGCGACT GCACTT--GT GCAGTTATGA ATTATTCCAT GTTGAATGCT
Go_11-3      ATCCCCTGTT C-GCCAGGGC AGGGGCGACT GCACTT--GT GCAGTTATGA ATTATTCCAT GTTGAATGCT
Go_11-5      ATCCCCTGTT C-GCCAGGGC AGGGGCGACT GCACTT--GT GCAGTTATGA ATTATTCCAT GTTGAATGCT
Go_13-5      ATCCCCTGTT C-GCCAGGGC AGGGGCGACT GCACTT--GT GCAGTTATGA ATTATTCCAT GTTGAATGCT
Go_14-2      ATCCCCTGTT C-GCCAGGGC AGGGGCGACT GCACTT--GT GCAGTTATGA ATTATTCCAT GTTGAATGCT
Go_14-3      ATCCCCTGTT C-GCCAGGGC AGGGGCGACT GCACTT--GT GCAGTTATGA ATTATTCCAT GTTGAATGCT
Go_14-4      ATCCCCTGTT C-GCCAGGGC AGGGGCGACT GCACTT--GT GCAGTTATGA ATTATTCCAT GTTGAATGCT
Pa_06-2      TTCCACTCTC TTTCCTAGAT TGTGGCGACT AGATTTCGAT CTAGTTATCA ATTATTCCAT TCTGAATGCT
Pa_14-4      CTCTGCTTTT C-TTCGGGAC AGCAGCGACC ACACTCCGGT GCGGTTAATA ATTATTCCAT GCTGAATGCT
Pa_14-6      CTCTGCTTTT C-TTCGGGAC AGCAGCGACC ACACTCCGGT GCGGTTAATA ATTATTCCAT GCTGAATGCT
Pa_16-1      CTCCACTGTC A-ATCTGG-T AGTGGCGACC ACACCTCGGT GCGGTTTCGA ACTATTCCAT ACTGAAAGCT
Pa_19-1      CTCCACCTTC C-CTCGGGAT TGTGGCGACC ACACTTCGGT GCGGTTAATA ATTATTCCAT TCTGAATGCT
Pa_19-2      CTCCACCTTC C-CTCGGGAT TGTGGCGACC ACACTTCGGT GCGGTTAATA ATTATTCCAT TCTGAATGCT
Pa_19-3      CTCCACCTTC C-CTCGGGAT TGTGGCGACC ACACTTCGGT GCGGTTAATA ATTATTCCAT TCTGAATGCT
Pa_21-2      TTCCACTTTC T-TTCGAGAC TGTGGCGACC ACATCACGAT GTGGTTTAGA ATTATTCCAT TCTGAATGCT
Pa_22-3      TTCCACTTTC T-TTCGAGAC TGTGGCGACC ACATCACGAT GTGGTTTAGA ATTATTCCAT TCTGAATGCT
Pa_39-1      CTCTGCTGTT CATATTGGAC AGTAGCGACC AGACTCCGGT TTGGTTAATA ATTATTCCAT GCTGAATGCT
Pa_39-5      CTCTGCTGTT CATATTGGAC AGTAGCGACC AGACTCCGGT TTGGTTAATA ATTATTCCAT GCTGAATGCT
Pa_50-11     TTCCACTCTC TTTCCAAGAT TGTGGCGACT AGATTTCGAT CTAGTTATCA ATTATTCCAT TCTGAATGCT
Pa_50-12     TTCCACTCTC TTTCCAAGAT TGTGGCGACT AGATTTCGAT CTAGTTATCA ATTATTCCAT TCTGAATGCT
Pa_50-19     TTCCACTCTC TTTCCAAGAT TGTGGCGACT AGATTTCGAT CTAGTTATCA ATTATTCCAT TCTGAATGCT
Pa_50-2      TTCCACTCTC TTTCCAAGAT TGTGGCGACT AGATTTCGAT CTAGTTATCA ATTATTCCAT TCTGAATGCT
Pa_57-2      TTCCACTCTC TTTCCTAGAT TGTGGCGACT AGATTTCGAT CTAGTTATCA ATTATTCCAT TCTGAATGCT
Pa_57-3      TTCCACTCTC TTTCCAAGAT TGTGGCGACT AGATTTCGAT CTAGTTATCA ATTATTCCAT TCTGAATGCT
Pa_57-5      TTCCACTCTC TTTCCTAGAT TGTGGCGACT AGATTTCGAT CTAGTTATCA ATTATTCCAT TCTGAATGCT
Pa_62-19     CTCCACTTTC C-TTCGGGAT TGTGGCGACC ACACCTCGGT GCGGTTAAGA ATTATTCCAT TCTGAATGCT
Pa_62-3      CTCCACTTTC C-TTCGGGAT TGTGGCGACC ACACCTCGGT GCGGTTAAGA ATTATTCCAT TCTGAATGCT
Pa_63-2      TTCCACTTTC T-TTGGAGAC TGTGGCGACC ACATCACGAT GTGGTTTAGA ATTATTCCAT TCTGAATGCT
Pa_63-3      TTCCACTTTC T-TTGGAGAC TGTGGCGACC ACATCACGAT GTGGTTTAGA ATTATTCCAT TCTGAATGCT
Pa_63-4      CTCCACTGTT C-TTCGGGAC AGTGGCGACC AGGCTCCGGT CTGGTTAATA ATTATTCCAT GCTGAATGCT
Pa_64-1      CTCCTCTTTC C-CTCGGGAC TGAGGCGACC ACACTTCTGT GCGGTTAGTA ATTATTCCAT TCTGAATGCT
Pa_64-2      CTCCTCTTTC C-CTCGGGAC TGAGGCGACC ACACTTCTGT GCGGTTAGTA ATTATTCCAT TCTGAATGCT
Pa_64-3      CTCCTCTTTC C-CTCGGGAC TGAGGCGACC ACACTTCTGT GCGGTTAGTA ATTATTCCAT TCTGAATGCT
Pa_64-4      CTCCTCTTTC C-CTCGGGAC TGAGGCGACC ACACTTCTGT GCGGTTAGTA ATTATTCCAT TCTGAATGCT
Pa_79-4      CTCCACATCT C-TTCTGGGA TGTGGCGGCC AGGCTTCGGC TTGGCTATGA ATTATTCCAT TCTGAATGCT
```

```
             211
Bd_06-10     GGAATAAGGA CAATTGTAGA ATCCCGAGGA TTTT-----T CAAGTAACCA ATGAGAATTT CTGACTTATC
Bd_09-1      GGAATAAGGA CAATTGTAGA ATCCCGAGGA TTTT-----T CAAGTAACCA ATGAGAATTT CTGACTTATC
Bd_09-2      GGAATAAGGA CAATTGTAGA ATCCCGAGGA TTTT-----T CAAGTAACCA ATGAGAATTT CTGACTTATC
Bd_09-3      GGAATAAGGA CAATTGTAGA ATCCCGAGGA TTTT-----T CAAGTAACCA ATGAGAATTT CTGACTTATC
Bd_10-1      GGAATAAGGA CAATTGTAGA ATCCCGAGGA TTTT-----T CAAGTAACCA ATGAGAATTT CTGACTTATC
Bd_10-2      GGAATAAGGA CAATTGTAGA ATCCCGAGGA TTTT-----T CAAGTAACCA ATGAGAATTT CTGACTTATC
Bd_10-2b     GGAATAAGGA CAATTGTAGA ATCCCGAGGA TTTT-----T CAAGTAACCA ATGAGAATTT CTGACTTATC
Bd_11-2      GGAATAAGGA CAATTGTAGA ATCCCGAGGA TTTT-----T CAAGTAACCA ATGAGAATTT CTGACTTATC
Bd_11-6      GGAATAAGGA CAATTGTAGA ATCCCGAGGA TTTT-----T CAAGTAACCA ATGAGAATTT CTGACTTATC
Bd_12-2      GGAATAAGGA CAATTGTAGA ATCCCGAGGA TTTT-----T CAAGTAACCA ATGAGAATTT CTGACTTATC
Bd_13-1      GGAATAAGGA CAATTGTAGA ATCCCGAGGA TTTT-----T CAAGTAACCA ATGAGAATTT CTGACTTATC
Bd_13-4      GGAATAAGGA CAATTGTAGA ATCCCGAGGA TTTT-----T CAAGTAACCA ATGAGAATTT CTGACTTATC
Bd_13-5      GGAATAAGGA CAATTGTAGA ATCCCGAGGA TTTT-----T CAAGTAACCA ATGAGAATTT CTGACTTATC
Bd_14-1      GGAATAAGGA CAATTGTAGA ATCCCGAGGA TTTT-----T CAAGTAACCA ATGAGAATTT CTGACTTATC
Bd_14-2      GGAATAAGGA CAATTGTAGA ATCCCGAGGA TTTT-----T CAAGTAACCA ATGAGAATTT CTGACTTATC
Bd_19-5      GGAATAAGGA CAATTGTAGA ATCCCGAGGA -TTT-----T CAAGTAACCA ATGAGAATTT CTGACTTATC
Bd_19-6      GGAATAAGGA CAATTGTAGA ATCCCGAGGA TTTT-----T CAAGTAACCA ATGAGAATTT CTGACTTATC
Bd_20-1      GGAATAAGGA CAATTGTAGA ATCCCGAGGA TTTT-----T CAAGTAACCA ATGAGAATTT CTGACTTATC
Bd_20-2      GGAATAAGGA CAATTGTAGA ATCCCGAGGA TTTT-----T CAAGTAACCA ATGAGAATTT CTGACTTATC
Bd_22-1      GGAATAAGGA CAATTGTAGA ATCCCGAGGA TTTT-----T CAAGTAACCA ATGAGAATTT CTGACTTATC
Go_06-1      GGAATAAGGA CAATTGTAGA ATCCTGAGGA TT----ACAA CAAGTAACCA ATGAGAATAT CTGACCTATC
Go_06-9      GGAATAAGGA CAATTGTAGA ATCCTGAGGA TT----ACAA CAAGTAACCA ATGAGAATAT CTGACCTATC
Go_07-1      GGAATAAGGA CAATTGTAGA ATCCTGAGGA TT----ACAA CAAGTAACCA ATGAGAATAT CTGACCTATC
Go_07-5      GGAATAAGGA CAATTGTAGA ATCCTGAGGA TT----ACAA CAAGTAACCA ATGAGAATAT CTGACCTATC
Go_07-6      GGAATAAGGA CAATTGTAGA ATCCTGAGGA TT----ACAA CAAGTAACCA ATGAGAATAT CTGACCTATC
Go_07-8      GGAATAAGGA CAATTGTAGA ATCCTGAGGA TT----ACAA CAAGTAACCA ATGAGAATAT CTGACCTATC
Go_09-2      GGAATAAGGA CAATTGTAGA ATCCCGAGGA TTTT-----T CAAGTAACCA ATGAGAATTT CTGACTTATC
Go_09-3      GGAATAAGGA CAATTGTAGA ATCCCGAGGA TTTT-----T CAAGTAACCA ATGAGAATTT CTGACTTATC
Go_09-4      GGAATAAGGA CAATTGTAGA ATCCCGAGGA TTTT-----T CAAGTAACCA ATGAGAATTT CTGACTTATC
Go_10-1      GGAATAAGGA CAATTGTAGA ATCCTGAGGA TT----ACAA CAAGTAACCA ATGAGAATAT CTGACCTATC
Go_10-3      GGAATAAGGA CAATTGTAGA ATCCTGAGGA TT----ACAA CAAGTAACCA ATGAGAATAT CTGACCTATC
Go_11-3      GGAATAAGGA CAATTGTAGA ATCCTGAGGA TT----ACAA CAAGTAACCA ATGAGAATAT CTGACCTATC
Go_11-5      GGAATAAGGA CAATTGTAGA ATCCTGAGGA TT----ACAA CAAGTAACCA ATGAGAATAT CTGACCTATC
Go_13-5      GGAATAAGGA CAATTGTAGA ATCCTGAGGA TT----ACAA CAAGTAACCA ATGAGAATAT CTGACCTATC
Go_14-2      GGAATAAGGA CAATTGTAGA ATCCTGAGGA TT----ACAA CAAGTAACCA ATGAGAATAT CTGACCTATC
Go_14-3      GGAATAAGGA CAATTGTAGA ATCCTGAGGA TT----ACAA CAAGTAACCA ATGAGAATAT CTGACCTATC
Go_14-4      GGAATAAGGA CAATTGTAGA ATCCTGAGGA TT----ACAA CAAGTAACCA ATGAGAATAT CTGACCTATC
Pa_06-2      GGAATAAGGA CAATTGTAGA ATCCATCGGA TTTT-----C CAAGTAACCA ATGAGAATTT CTGACTTATC
Pa_14-4      GGAATAAGGA CAATTGTAGA ATCCTAAGGA TTTTAACTAA CAAGTAACCA ATGAGAATTT CTGACTTATC
Pa_14-6      GGAATAAGGA CAATTGTAGA ATCCTAAGGA TTTTAACTAA CAAGTAACCA ATGAGAATTT CTGACTTATC
Pa_16-1      GGAATAGGGA CAATTGTAGA ATCCAACGGA TTTTAACTAA CAAGTAACCA ATGAGAATTT CTGACTTATC
Pa_19-1      GGAATAAGGA CAATTGTAGA ATCCTACGGA TTTTAACTGA CAAGTAACCA ATGAGAATTT CTGACTTATC
Pa_19-2      GGAATAAGGA CAATTGTAGA ATCCTACGGA TTTTAACTGA CAAGTAACCA ATGAGAATTT CTGACTTATC
Pa_19-3      GGAATAAGGA CAATTGTAGA ATCCTACGGA TTTTAACTGA CAAGTAACCA ATGAGAATTT CTGACTTATC
Pa_21-2      GGAATAAGGA CAATTGTAGA ATCCTGGGGA TTTT-----T CAAGTAACCA ATGAGAATTT CTGACTTATC
Pa_22-3      GGAATAAGGA CAATTGTAGA ATCCTGGGGA TTTT-----T CAAGTAACCA ATGAGAATTT CTGACTTATC
Pa_39-1      GGAATGAGGA CAATTGTAGA ATCCTTCGGG TTTTAACTAA CAAGTAACCA ATGAGAATTT CTGACTTATC
Pa_39-5      GGAATGAGGA CAATTGTAGA ATCCTTCGGA TTTTAACTAA CAAGTAACCA ATGAGAATTT CTGACTTATC
Pa_50-11     GGAATAAGGA CAATTGTAGA ATCCATCGGA TTTT-----C CAAGTAACCA ATGAGAATTT CTGACTTATC
Pa_50-12     GGAATAAGGA CAATTGTAGA ATCCATCGGA TTTT-----C CAAGTAACCA ATGAGAATTT CTGACTTATC
Pa_50-19     GGAATAAGGA CAATTGTAGA ATCCATCGGA TTTT-----C CAAGTAACCA ATGAGAATTT CTGACTTATC
Pa_50-2      GGAATAAGGA CAATTGTAGA ATCCATCGGA TTTT-----C CAAGTAACCA ATGAGAATTT CTGACTTATC
Pa_57-2      GGAATAAGGA CAATTGTAGA ATCCATCGGA TTTT-----C CAAGTAACCA ATGAGAATTT CTGACTTATC
Pa_57-3      GGAATAAGGC CAATTGTAGA ATCCATCGGA TTTT-----C CAAGTAACCA ATGGGAATTT CTGACTTATC
Pa_57-5      GGAATAAGGA CAATTGTAGA ATCCATCGGA TTTT-----C CAAGTAACCA ATGAGAATTT CTGACTTATC
Pa_62-19     GGAATAAGGA CAATTGTAGA ATCCTACGGA TTTTAACTAA CAAGTAACCA ATGAGAATTT CTGACTTATC
Pa_62-3      GGAATAAGGA CAATTGTAGA ATCCTACGGA TTTTAACTAA CAAGTAACCA ATGAGAATTT CTGACTTATC
Pa_63-2      GGAATAAGGA CAATTGTAGA ATCCGGGGGA TTTT-----T CAAGTAACCA ATGAGAATTT CTGACTTATC
Pa_63-3      GGAATAAGGA CAATTGTAGA ATCCGGGGGA TTTT-----T CAAGTAACCA ATGAGAATTT CTGACTTATC
Pa_63-4      GGAATAAGGA CAATTGTAGA ATCCTGGGGA TTTTAACTGA CAAGTAACCA ATGAGAATTT CTGACTTATC
Pa_64-1      GGAATAAGGA CAATTGTAGA ATCCTGTGGA TTTTAACTAA CAAGTAACCA ATGAGAATTT CTGACTTATC
Pa_64-2      GGAATAAGGA CAATTGTAGA ATCCTGTGGA TTTTAACTAA CAAGTAACCA ATGAGAATTT CTGACTTATC
Pa_64-3      GGAATAAGGA CAATTGTAGA ATCCTGTGGA TTTTAACTAA CAAGTAACCA ATGAGAATTT CTGACTTATC
Pa_64-4      GGAATAAGGA CAATTGTAGA ATCCTGTGGA TTTTAACTAA CAAGTAACCA ATGAGAATTT CTGACTTATC
Pa_79-4      GGAATAAGGA CAATTGTAGA ATCCAGGGGA TTTTAACTAA CAAGTAACCA ATGAGAATTT CGGACTTATC
```

```
             281
Bd_06-10     AACCTGTAGG TAAGGTAGTG GCTTACCTTG GTGATCACGA GTAACGAGAA ATAAGGGTTT GACATCGGAG
Bd_09-1      AACCTGTAGG TAAGGTAGTG GCTTACCTTG GTGATCACGA GTAACGAGAA ATAAGGGTTT GACATCGGAG
Bd_09-2      AACCTGTAGG TAAGGTAGTG GCTTACCTTG GTGATCACGA GTAACGAGAA ATAAGGGTTT GACATCGGAG
Bd_09-3      AACCTGTAGG TAAGGTAGTG GCTTACCTTG GTGATCACGA GTAACGAGAA ATAAGGGTTT GACATCGGAG
Bd_10-1      AACCTGTAGG TAAGGTAGTG GCTTACCTTG GTGATCACGA GTAACGAGAA ATAAGGGTTT GACATCGGAG
Bd_10-2      AACCTGTAGG TAAGGTAGTG GCTTACCTTG GTGATCACGA GTAACGAGAA ATAAGGGTTT GACATCGGAG
Bd_10-2b     AACCTGTAGG TAAGGTAGTG GCTTACCTTG GTGATCACGA GTAACGAGAA ATAAGGGTTT GACATCGGAG
Bd_11-2      AACCTGTAGG TAAGGTAGTG GCTTACCTTG GTGATCACGA GTAACGAGAA ATAAGGGTTT GACATCGGAG
Bd_11-6      AACCTGTAGG TAAGGTAGTG GCTTACCTTG GTGATCACGA GTAACGAGAA ATAAGGGTTT GACATCGGAG
Bd_12-2      AACCTGTAGG TAAGGTAGTG GCTTACCTTG GTGATCACGA GTAACGAGAA ATAAGGGTTT GACATCGGAG
Bd_13-1      AACCTGTAGG TAAGGTAGTG GCTTACCTTG GTGATCACGA GTAACGAGAA ATAAGGGTTT GACATCGGAG
Bd_13-4      AACCTGTAGG TAAGGTAGTG GCTTACCTTG GTGATCACGA GTAACGAGAA ATAAGGGTTT GACATCGGAG
Bd_13-5      AACCTGTAGG TAAGGTAGTG GCTTACCTTG GTGATCACGA GTAACGAGAA ATAAGGGTTT GACATCGGAG
Bd_14-1      AACCTGTAGG TAAGGTAGTG GCTTACCTTG GTGATCACGA GTAACGAGAA ATAAGGGTTT GACATCGGAG
Bd_14-2      AACCTGTAGG TAAGGTAGTG GCTTACCTTG GTGATCACGA GTAACGAGAA ATAAGGGTTT GACATCGGAG
Bd_19-5      AACCTGTAGG TAAGGTAGTG GCTTACCTTG GTGATCACGA GTAACGAGAA ATAAGGGTTT GACATCGGAG
Bd_19-6      AACCTGTAGG TAAGGTAGTG GCTTACCTTG GTGATCACGA GTAACGAGAA ATAAGGGTTT GACATCGGAG
Bd_20-1      AACCTGTAGG TAAGGTAGTG GCTTACCTTG GTGATCACGA GTAACGAGAA ATAAGGGTTT GACATCGGAG
Bd_20-2      AACCTGTAGG TAAGGTAGTG GCTTACCTTG GTGATCACGA GTAACGAGAA ATAAGGGTTT GACATCGGAG
Bd_22-1      AACCTGTAGG TAAGGTAGTG GCTTACCTTG GTGATCACGA GTAACGAGAA ATAAGGGTTT GACATCGGAG
Go_06-1      AACCAGAAGG TAAGGTAGTG GCTTACCTTG GTTATCACGG GTAACGAGAA ATAAGGGTTT GACATCGGAG
Go_06-9      AACCAGAAGG TAAGGTAGTG GCTTACCTTG GTTATCACGG GTAACGAGAA ATAAGGGTTT GACATCGGAG
Go_07-1      AACCAGAAGG TAAGGTAGTG GCTTACCTTG GTTATCACGG GTAACGAGAA ATAAGGGTTT GACATCGGAG
Go_07-5      AACCAGAAGG TAAGGTAGTG GCTTACCTTG GTTATCACGG GTAACGAGAA ATAAGGGTTT GACATCGGAG
Go_07-6      AACCAGAAGG TAAGGTAGTG GCTTACCTTG GTTATCACGG GTAACGAGAA ATAAGGGTTT GACATCGGAG
Go_07-8      AACCAGAAGG TAAGGTAGTG GCTTACCTTG GTTATCACGG GTAACGAGAA ATAAGGGTTT GACATCGGAG
Go_09-2      AACCTGTAGG TAAGGTAGTG GCTTACCTTG GTGATCACGA GTAACGAGAA ATAAGGGTTT GACATCGGAG
Go_09-3      AACCTGTAGG TAAGGTAGTG GCTTACCTTG GTGATCACGA GTAACGAGAA ATAAGGGTTT GACATCGGAG
Go_09-4      AACCTGTAGG TAAGGTAGTG GCTTACCTTG GTGATCACGA GTAACGAGAA ATAAGGGTTT GACATCGGAG
Go_10-1      AACCAGAAGG TAAGGTAGTG GCTTACCTTG GTTATCACGG GTAACGAGAA ATAAGGGTTT GACATCGGAG
Go_10-3      AACCAGAAGG TAAGGTAGTG GCTTACCTTG GTTATCACGG GTAACGAGAA ATAAGGGTTT GACATCGGAG
Go_11-3      AACCAGAAGG TAAGGTAGTG GCTTACCTTG GTTATCACGG GTAACGAGAA ATAAGGGTTT GACATCGGAG
Go_11-5      AACCAGAAGG TAAGGTAGTG GCTTACCTTG GTTATCACGG GTAACGAGAA ATAAGGGTTT GACATCGGAG
Go_13-5      AACCAGAAGG TAAGGTAGTG GCTTACCTTG GTTATCACGG GTAACGAGAA ATAAGGGTTT GACATCGGAG
Go_14-2      AACCAGAAGG TAAGGTAGTG GCTTACCTTG GTTATCACGG GTAACGAGAA ATAAGGGTTT GACATCGGAG
Go_14-3      AACCAGAAGG TAAGGTAGTG GCTTACCTTG GTTATCACGG GTAACGAGAA ATAAGGGTTT GACATCGGAG
Go_14-4      AACCAGAAGG TAAGGTAGTG GCTTACCTTG GTTATCACGG GTAACGAGAA ATAAGGGTTT GACATCGGAG
Pa_06-2      AACCTGTAGG TAAGGTAGTG GCTTACCTTG GTGATCACGA GTAACGAGAA ATAAGGGTTT GACATCGGAG
Pa_14-4      AACCTGTAGG TAAGGTAGTG GCTTACCTTG GTTATCACGA GTAACGAGAA ATAAGGGTTT GACATCGGAG
Pa_14-6      AACCTGTAGG TAAGGTAGTG GCTTACCTTG GTTATCACGA GTAACGAGAA ATAAGGGTTT GACATCGGAG
Pa_16-1      AACCAGTAGG TAAGGTAGTG GCTTACCTTG GTGATCACGA GTAACGAGAA ATAAGGGTTT GACATCGGAG
Pa_19-1      AACCAGTAGG TAAGGTAGTG GCTTACCTTG GTGATCACGA GTAACGAGAA ATAAGGGTTT GACATCGGAG
Pa_19-2      AACCAGTAGG TAAGGTAGTG GCTTACCTTG GTGATCACGA GTAACGAGAA ATAAGGGTTT GACATCGGAG
Pa_19-3      AACCAGTAGG TAAGGTAGTG GCTTACCTTG GTGATCACGA GTAACGAGAA ATAAGGGTTT GACATCGGAG
Pa_21-2      AACCTGTAGG TAAGGTAGTG GCTTACCTTG GTGATCACGA GTAACGAGAA ATAAGGGTTT GACATCGGAG
Pa_22-3      AACCTGTAGG TAAGGTAGTG GCTTACCTTG GTGATCACGA GTAACGAGAA ATAAGGGTTT GACATCGGAG
Pa_39-1      AACCTGTAGG TAAGGTAGTG GCTTACCTTG GTAATCACGA GTAACGAGAA ATAAGGGTTT GACATCGGAG
Pa_39-5      AACCTGTAGG TAAGGTAGTG GCTTACCTTG GTAATCACGA GTAACGAGAA ATAAGGGTTT GACATCGGAG
Pa_50-11     AACCTGTAGG TAAGGTAGTG GCTTACCTTG GTGATCACGA GTAACGAGAA ATAAGGGTTT GACATCGGAG
Pa_50-12     AACCTGTAGG TAAGGTAGTG GCTTACCTTG GTGATCAAGA GTAACGAGAA ATAAGGGTTT GACATCGGAG
Pa_50-19     AACCTGTAGG TAAGGTAGTG GCTTACCTTG GTGATCACGA GTAACGAGAA ATAAGGGTTT GACATCGGAG
Pa_50-2      AACCTGTAGG TAAGGTAGTG GCTTACCTTG GTGATCACGA GTAACGAGAA ATAAGGGTTT GACATCGGAG
Pa_57-2      AACCTGTAGG TAAGGTAGTG GCTTACCTTG GTGATCACGA GTAACGAGAA ATAAGGGTTT GACATCGGAG
Pa_57-3      AACCTGTAGG TAAGGTAGTG GCTTACCTTG GTGATCACGA GTAACGAGAA ATAAGGGTTT GACATCGGAG
Pa_57-5      AACCTGTAGG TAAGGTAGTG GCTTACCTTG GTGATCACGA GTAACGAGAA ATAAGGGTTT GACATCGGAG
Pa_62-19     AACCAGTAGG TAAGGTAGTG GCTTACCTTG GTGATCACGA GTAACGAGAA ATAAGGGTTT GACATCGGAG
Pa_62-3      AACCAGTAGG TAAGGTAGTG GCTTACCTTG GTGATCACGA GTAACGAGAA ATAAGGGTTT GACATCGGAG
Pa_63-2      AACCTGTAGG TAAGGTAGTG GCTTACCTTG GTGATCACGA GTAACGAGAA ATAAGGGTTT GACATCGGAG
Pa_63-3      AACCTGTAGG TAAGGTAGTG GCTTACCTTG GTGATCACGA GTAACGAGAA ATAAGGGTTT GACATCGGAG
Pa_63-4      AACCTGTAGG TAAGGTAGTG GCTTACCTTG GTTATCACGA GTAACGAGAA ATAAGGGTTT GACATCGGAG
Pa_64-1      AACCTGTAGG TAAGGTAGTG GCTTACCTTG GTGATCACGA GTAACGAGAA ATAAGGGTTT GACATCGGAG
Pa_64-2      AACCTGTAGG TAAGGTAGTG GCTTACCTTG GTGATCACGA GTAACGAGAA ATAAGGGTTT GACATCGGAG
Pa_64-3      AACCTGTAGG TAAGGTAGTG GCTTACCTTG GTGATCACGA GTAACGAGAA ATAAGGGTTT GACATCGGAG
Pa_64-4      AACCTGTAGG TAAGGTAGTG GCTTACCTTG GTGATCACGA GTAACGAGAA ATAAGGGTTT GACATCGGAG
Pa_79-4      AACCTGTAGG TAAGGTAGTG GCTTACCTTG GTGATCACGA GTAACGAGAA ATAAGGGTTT GACATCGGAG
```

```
            351
Bd_06-10    AGGGAGCCTT CAAAATGGCT ACCACTTCCA AGGAAGGCAG CAGGCGCGAA AATTACCCAC TTGGGACTCC
Bd_09-1     AGGGAGCCTT CAAAATGGCT ACCACTTCCA AGGAAGGCAG CAGGCGCGAA AATTACCCAC TTGGGACTCC
Bd_09-2     AGGGAGCCTT CAAAATGGCT ACCACTTCCA AGGAAGGCAG CAGGCGCGAA AATTACCCAC TTGGGACTCC
Bd_09-3     AGGGAGCCTT CAAAATGGCT ACCACTTCCA AGGAAGGCAG CAGGCGCGAA AATTACCCAC TTGGGACTCC
Bd_10-1     AGGGAGCCTT CAAAATGGCT ACCACTTCCA AGGAAGGCAG CAGGCGCGAA AATTACCCAC TTGGGACTCC
Bd_10-2     AGGGAGCCTT CAAAATGGCT ACCACTTCCA AGGAAGGCAG CAGGCGCGAA AATTACCCAC TTGGGACTCC
Bd_10-2b    AGGGAGCCTT CAAAATGGCT ACCACTTCCA AGGAAGGCAG CAGGCGCGAA AATTACCCAC TTGGGACTCC
Bd_11-2     AGGGAGCCTT CAAAATGGCT ACCACTTCCA AGGAAGGCAG CAGGCGCGAA AATTACCCAC TTGGGACTCC
Bd_11-6     AGGGAGCCTT CAAAATGGCT ACCACTTCCA AGGAAGGCAG CAGGCGCGAA AATTACCCAC TTGGGACTCC
Bd_12-2     AGGGAGCCTT CAAAATGGCT ACCACTTCCA AGGAAGGCAG CAGGCGCGAA AATTACCCAC TTGGGACTCC
Bd_13-1     AGGGAGCCTT CAAAATGGCT ACCACTTCCA AGGAAGGCAG CAGGCGCGAA AATTACCCAC TTGGGACTCC
Bd_13-4     AGGGAGCCTT CAAAATGGCT ACCACTTCCA AGGAAGGCAG CAGGCGCGAA AATTACCCAC TTGGGACTCC
Bd_13-5     AGGGAGCCTT CAAAATGGCT ACCACTTCCA AGGAAGGCAG CAGGCGCGAA AATTACCCAC TTGGGACTCC
Bd_14-1     AGGGAGCCTT CAAAATGGCT ACCACTTCCA AGGAAGGCAG CAGGCGCGAA AATTACCCAC TTGGGACTCC
Bd_14-2     AGGGAGCCTT CAAAATGGCT ACCACTTCCA AGGAAGGCAG CAGGCGCGAA AATTACCCAC TTGGGACTCC
Bd_19-5     AGGGAGCCTT CAAAATGGCT ACCACTTCCA AGGAAGGCAG CAGGCGCGAA AATTACCCAC TTGGGACTCC
Bd_19-6     AGGGAGCCTT CAAAATGGCT ACCACTTCCA AGGAAGGCAG CAGGCGCGAA AATTACCCAC TTGGGACTCC
Bd_20-1     AGGGAGCCTT CAAAATGGCT ACCACTTCCA AGGAAGGCAG CAGGCGCGAA AATTACCCAC TTGGGACTCC
Bd_20-2     AGGGAGCCTT CAAAATGGCT ACCACTTCCA AGGAAGGCAG CAGGCGCGAA AATTACCCAC TTGGGACTCC
Bd_22-1     AGGGAGCCTT CAAAATGGCT ACCACTTCCA AGGAAGGCAG CAGGCGCGAA AATTACCCAC TTGGGACTCC
Go_06-1     AGGGAGCCTT CAAAATGGCT ACCACTTCCA AGGAAGGCAG CAGGCGCGAA AATTACCCAC TTGGGACACC
Go_06-9     AGGGAGCCTT CAAAATGGCT ACCACTTCCA AGGAAGGCAG CAGGCGCGAA AATTACCCAC TTGGGACACC
Go_07-1     AGGGAGCCTT CAAAATGGCT ACCACTTCCA AGGAAGGCAG CAGGCGCGAA AATTACCCAC TTGGGACACC
Go_07-5     AGGGAGCCTT CAAAATGGCT ACCACTTCCA AGGAAGGCAG CAGGCGCGAA AATTACCCAC TTGGGACACC
Go_07-6     AGGGAGCCTT CAAAATGGCT ACCACTTCCA AGGAAGGCAG CAGGCGCGAA AATTACCCAC TTGGGACACC
Go_07-8     AGGGAGCCTT CAAAATGGCT ACCACTTCCA AGGAAGGCAG CAGGCGCGAA AATTACCCAC TTGGGACACC
Go_09-2     AGGGAGCCTT CAAAATGGCT ACCACTTCCA AGGAAGGCAG CAGGCGCGAA AATTACCCAC TTGGGACTCC
Go_09-3     AGGGAGCCTT CAAAATGGCT ACCACTTCCA AGGAAGGCAG CAGGCGCGAA AATTACCCAC TTGGGACTCC
Go_09-4     AGGGAGCCTT CAAAATGGCT ACCACTTCCA AGGAAGGCAG CAGGCGCGAA AATTACCCAC TTGGGACTCC
Go_10-1     AGGGAGCCTT CAAAATGGCT ACCACTTCCA AGGAAGGCAG CAGGCGCGAA AATTACCCAC TTGGGACACC
Go_10-3     AGGGAGCCTT CAAAATGGCT ACCACTTCCA AGGAAGGCAG CAGGCGCGAA AATTACCCAC TTGGGACACC
Go_11-3     AGGGAGCCTT CAAAATGGCT ACCACTTCCA AGGAAGGCAG CAGGCGCGAA AATTACCCAC TTGGGACACC
Go_11-5     AGGGAGCCTT CAAAATGGCT ACCACTTCCA AGGAAGGCAG CAGGCGCGAA AATTACCCAC TTGGGACACC
Go_13-5     AGGGAGCCTT CAAAATGGCT ACCACTTCCA AGGAAGGCAG CAGGCGCGAA AATTACCCAC TTGGGACACC
Go_14-2     AGGGAGCCTT CAAAATGGCT ACCACTTCCA AGGAAGGCAG CAGGCGCGAA AATTACCCAC TTGGGACACC
Go_14-3     AGGGAGCCTT CAAAATGGCT ACCACTTCCA AGGAAGGCAG CAGGCGCGAA AATTACCCAC TTGGGACACC
Go_14-4     AGGGAGCCTT CAAAATGGCT ACCACTTCCA AGGAAGGCAG CAGGCGCGAA AATTACCCAC TTGGGACACC
Pa_06-2     AGGGAGCCTT AAAAATGGCT ACCACTTCCA AGGAAGGCAG CAGGCGCGAA AATTACCCAC TTGGGACTCC
Pa_14-4     AGGGAGCCTT AAAAATGGCT ACCACTTCCA AGGAAGGCAG CAGGCGCGAA AATTACCCAC TTGGGACGCC
Pa_14-6     AGGGAGCCTT AAAAATGGCT ACCACTTCCA AGGAAGGCAG CAGGCGCGAA AATTACCCAC TTGGGACGCC
Pa_16-1     AGGGAGCCTT AAAAATGGCT ACCACTTCCA AGGAAGGCAG CAGGCGCGAA AATTACCCAC TTGGGACACC
Pa_19-1     AGGGAGCCTT AAAAATGGCT ACCACTTCCA AGGAAGGCAG CAGGCGCGAA AATTACCCAC TTGGGACACC
Pa_19-2     AGGGAGCCTT AAAAATGGCT ACCACTTCCA AGGAAGGCAG CAGGCGCGAA AATTACCCAC TTGGGACACC
Pa_19-3     AGGGAGCCTT AAAAATGGCT ACCACTTCCA AGGAAGGCAG CAGGCGCGAA AATTACCCAC TTGGGACACC
Pa_21-2     AGGGAGCCTT CAAAATGGCT ACCACTTCCA AGGAAGGCAG CAGGCGCGAA AATTACCCAC TTGGGACTCC
Pa_22-3     AGGGAGCCTT CAAAATGGCT ACCACTTCCA AGGAAGGCAG CAGGCGCGAA AATTACCCAC TTGGGACTCC
Pa_39-1     AGGGAGCCTT AAAAATGGCT ACCACTTCCA AGGAAGGCAG CCGGCGCGAA AATTACCCAC TTGGGACGCC
Pa_39-5     AGGGAGCCTT AAAAATGGCT ACCACTTCCA AGGAAGGCAG CAGGCGCGAA AATTACCCAC TTGGGACGCC
Pa_50-11    AGGGAGCCTT AAAAATGGCT ACCACTTCCA AGGAAGGCAG CAGGCGCGAA AATTACCCAC TTGGGACTCC
Pa_50-12    AGGGAGCCTT AAAAATGGCT ACCACTTCCA AGGAAGGCAG CAGGCGCGAA AATTACCCAC TTGGGACTCC
Pa_50-19    AGGGAGCCTT AAAAATGGCT ACCACTTCCA AGGAAGGCAG CAGGCGCGAA AATTACCCAC TTGGGACTCC
Pa_50-2     AGGGAGCCTT AAAAATGGCT ACCACTTCCA AGGAAGGCAG CAGGCGCGAA AATTACCCAC TTGGGACTCC
Pa_57-2     AGGGAGCCTT AAAAATGGCT ACCACTTCCA AGGAAGGCAG CAGGCGCGAA AATTACCCAC TTGGGACTCC
Pa_57-3     AGGGAGCCTT AAAAATGGCT ACCACTTCCA AGGAAGGCAG CAGGCGCGAA AATTACCCAC TTGGGACTCC
Pa_57-5     AGGGAGCCTT AAAAATGGCT ACCACTTCCA AGGAAGGCAG CAGGCGCGAA AATTACCCAC TTGGGACTCC
Pa_62-19    AGGGAGCCTT AAAAATGGCT ACCACTTCCA AGGAAGGCAG CAGGCGCGAA AATTACCCAC TTGGGACACC
Pa_62-3     AGGGAGCCTT AAAAATGGCT ACCACTTCCA AGGAAGGCAG CAGGCGCGAA AATTACCCAC TTGGGACACC
Pa_63-2     AGGGAGCCTT CAAAATGGCT ACCACTTCCA AGGAAGGCAG CAGGCGCGAA AATTACCCAC TTGGGACTCC
Pa_63-3     AGGGAGCCTT CAAAATGGCT ACCACTTCCA AGGAAGGCAG CAGGCGCGAA AATTACCCAC TTGGGACTCC
Pa_63-4     AGGGAGCCTT AAAAATGGCT ACCACTTCCA AGGAAGGCAG CAGGCGCGAA AATTACCCAC TTGGGACGCC
Pa_64-1     AGGGAGCCTT AAAAATGGCT ACCACTTCCA AGGAAGGCAG CAGGCGCGAA AATTACCCAC TTGGGACACC
Pa_64-2     AGGGAGCCTT AAAAATGGCT ACCACTTCCA AGGAAGGCAG CAGGCGCGAA AATTACCCAC TTGGGACACC
Pa_64-3     AGGGAGCCTT AAAAATGGCT ACCACTTCCA AGGAAGGCAG CAGGCGCGAA AATTACCCAC TTGGGACACC
Pa_64-4     AGGGAGCCTT AAAAATGGCT ACCACTTCCA AGGAAGGCAG CAGGCGCGAA AATTACCCAC TTGGGACACC
Pa_79-4     AGGGAGCCTT AAAAATGGCT ACCACTTCCA AGGAAGGCAG CAAGCGCGAA AATTACCCAC TTGGGACACC
```

```
            421
Bd_06-10    CAGAGGTAGT GAAGACAAAT AACTAAACTC TGTCCTATGG ATAGAGTTTA AGGGATGAGC TAGACGTTAA
Bd_09-1     CAGAGGTAGT GAAGACAAAT AACTAAACTC TGTCCTATGG ATAGAGTTTA AGGGATGAGC TAGACGTTAA
Bd_09-2     CAGAGGTAGT GAAGACAAAT AACTAAACTC TGTCCTATGG ATAGAGTTTA AGGGATGAGC TAGACGTTAA
Bd_09-3     CAGAGGTAGT GAAGACAAAT AACTAAACTC TGTCCTATGG ATAGAGTTTA AGGGATGAGC TAGACGTTAA
Bd_10-1     CAGAGGTAGT GAAGACAAAT AACTAAACTC TGTCCTATGG ATAGAGTTTA AGGGATGAGC TAGACGTTAA
Bd_10-2     CAGAGGTAGT GAAGACAAAT AACTAAACTC TGTCCTATGG ATAGAGTTTA AGGGATGAGC TAGACGTTAA
Bd_10-2b    CAGAGGTAGT GAAGACAAAT AACTAAACTC TGTCCTATGG ATAGAGTTTA AGGGATGAGC TAGACGTTAA
Bd_11-2     CAGAGGTAGT GAAGACAAAT AACTAAACTC TGTCCTATGG ATAGAGTTTA AGGGATGAGC TAGACGTTAA
Bd_11-6     CAGAGGTAGT GAAGACAAAT AACTAAACTC TGTCCTATGG ATAGAGTTTA AGGGATGAGC TAGACGTTAA
Bd_12-2     CAGAGGTAGT GAAGACAAAT AACTAAACTC TGTCCTATGG ATAGAGTTTA AGGGATGAGC TAGACGTTAA
Bd_13-1     CAGAGGTAGT GAAGACAAAT AACTAAACTC TGTCCTATGG ATAGAGTTTA AGGGATGAGC TAGACGTTAA
Bd_13-4     CAGAGGTAGT GAAGACAAAT AACTAAACTC TGTCCTATGG ATAGAGTTTA AGGGATGAGC TAGACGTTAA
Bd_13-5     CAGAGGTAGT GAAGACAAAT AACTAAACTC TGTCCTATGG ATAGAGTTTA AGGGATGAGC TAGACGTTAA
Bd_14-1     CAGAGGTAGT GAAGACAAAT AACTAAACTC TGTCCTATGG ATAGAGTTTA AGGGATGAGC TAGACGTTAA
Bd_14-2     CAGAGGTAGT GAAGACAAAT AACTAAACTC TGTCCTATGG ATAGAGTTTA AGGGATGAGC TAGACGTTAA
Bd_19-5     CAGAGGTAGT GAAGACAAAT AACTAAACTC TGTCCTATGG ATAGAGTTTA AGGGATGAGC TAGACGTTAA
Bd_19-6     CAGAGGTAGT GAAGACAAAT AACTAAACTC TGTCCTATGG ATAGAGTTTA AGGGATGAGC TAGACGTTAA
Bd_20-1     CAGAGGTAGT GAAGACAAAT AACTAAACTC TGTCCTATGG ATAGAGTTTA AGGGATGAGC TAGACGTTAA
Bd_20-2     CAGAGGTAGT GAAGACAAAT AACTAAACTC TGTCCTATGG ATAGAGTTTA AGGGATGAGC TAGACGTTAA
Bd_22-1     CAGAGGTAGT GAAGACAAAT AACTAAACTC TGTCCTATGG ATAGAGTTTA AGGGATGAGC TAGACGTTAA
Go_06-1     CAGAGGTAGT GAAGACAAAT AACTAAAAGG ACCCCTTTGG GGTTCTTTTA AGGGATGAGC TAGACGTTAA
Go_06-9     CAGAGGTAGT GAAGACAAAT AACTAAAAGG ACCCCTTTGG GGTTCTTTTA AGGGATGAGC TAGACGTTAA
Go_07-1     CAGAGGTAGT GAAGACAAAT AACTAAAAGG ACCCCTTTGG GGTTCTTTTA AGGGATGAGC TAGACGTTAA
Go_07-5     CAGAGGTAGT GAAGACAAAT AACTAAAAGG ACCCCTTTGG GGTTCTTTTA AGGGATGAGC TAGACGTTAA
Go_07-6     CAGAGGTAGT GAAGACAAAT AACTAAAAGG ACCCCTTTGG GGTTCTTTTA AGGGATGAGC TAGACGTTAA
Go_07-8     CAGAGGTAGT GAAGACAAAT AACTAAAAGG ACCCCTTTGG GGTTCTTTTA AGGGATGAGC TAGACGTTAA
Go_09-2     CAGAGGTAGT GAAGACAAAT AACTAAACTC TGTCCTATGG ATAGAGTTTA AGGGATGAGC TAGACGTTAA
Go_09-3     CAGAGGTAGT GAAGACAAAT AACTAAACTC TGTCCTATGG ATAGAGTTTA AGGGATGAGC TAGACGTTAA
Go_09-4     CAGAGGTAGT GAAGACAAAT AACTAAACTC TGTCCTATGG ATAGAGTTTA AGGGATGAGC TAGACGTTAA
Go_10-1     CAGAGGTAGT GAAGACAAAT AACTAAAAGG ACCCCTTTGG GGTTCTTTTA AGGGATGAGC TAGACGTTAA
Go_10-3     CAGAGGTAGT GAAGACAAAT AACTAAAAGG ACCCCTTTGG GGTTCTTTTA AGGGATGAGC TAGACGTTAA
Go_11-3     CAGAGGTAGT GAAGACAAAT AACTAAAAGG ACCCCTTTGG GGTTCTTTTA AGGGATGAGC TAGACGTTAA
Go_11-5     CAGAGGTAGT GAAGACAAAT AACTAAAAGG ACCCCTTTGG GGTTCTTTTA AGGGATGAGC TAGACGTTAA
Go_13-5     CAGAGGTAGT GAAGACAAAT AACTAAAAGG ACCCCTTTGG GGTTCTTTTA AGGGATGAGC TAGACGTTAA
Go_14-2     CAGAGGTAGT GAAGACAAAT AACTAAAAGG ACCCCTTTGG GGTTCTTTTA AGGGATGAGC TAGACGTTAA
Go_14-3     CAGAGGTAGT GAAGACAAAT AACTAAAAGG ACCCCTTTGG GGTTCTTTTA AGGGATGAGC TAGACGTTAA
Go_14-4     CAGAGGTAGT GAAGACAAAT AACTAAAAGG ACCCCTTTGG GGTTCTTTTA AGGGATGAGC TAGACGTTAA
Pa_06-2     CAGAGGTAGT GAAGACAAAT AACTAAATTC TGTCCTTTGG ATAGGATTTA AGGGATGAGC TAGACGTTAA
Pa_14-4     CAGAGGTAGT GAAGACAAAT AACTAAACCC TGTCCACTGG ATAGAGTTTA AGGGATGAGC TAGACGTTAA
Pa_14-6     CAGAGGTAGT GAAGACAAAT AACTAAACCC TGTCCACTGG ATAGAGTTTA AGGGATGAGC TAGACGTTAA
Pa_16-1     CAGAGGTAGT GAAGACAAAT AACTAAATGC TGTCCAATGG ATAGCGTTTA AGGGATGAGC TAGACGTTAA
Pa_19-1     CAGAGGTAGT GAAGACAAAT AACTAAATGC TGTCCACTGG ATAGCGTTTA AGGGATGAGC TAGACGTTAA
Pa_19-2     CAGAGGTAGT GAAGACAAAT AACTAAATGC TGTCCACTGG ATAGCGTTTA AGGGATGAGC TAGACGTTAA
Pa_19-3     CAGAGGTAGT GAAGACAAAT AACTAAATGC TGTCCACTGG ATAGCGTTTA AGGGATGAGC TAGACGTTAA
Pa_21-2     CAGAGGTAGT GAAGACAAAT AACTAAATTC TGTCCACTGG ATAGAGTTTA AGGGATGAGC TAGACGTTAA
Pa_22-3     CAGAGGTAGT GAAGACAAAT AACTAAATTC TGTCCACTGG ATAGAGTTTA AGGGATGAGC TAGACGTTAA
Pa_39-1     CAGAGGTAGT GAAGACAAAT AACTAAACGC TATCCACTGG ATAGTGTTTA AGGGATGAGC TAGACGTTAA
Pa_39-5     CAGAGGTAGT GAAGACAAAT AACTAAACGC TATCCACTGG ATAGTGTTTA AGGGATGAGC TAGACGTTAA
Pa_50-11    CAGAGGTAGT GAAGACAAAT AACTAAATTC TGTCCTTTGG ATAGGATTTA AGGGATGAGC TAGACGTTAA
Pa_50-12    CAGAGGTAGT GAAGACAAAT AATTAAATTC TGTCCTTTGG ATAGGATTTA AGGGATGAGC TAGACGTTAA
Pa_50-19    CAGAGGTAGT GAAGACAAAT AACTAAATTC TGTCCTTTGG ATAGGATTTA AGGGATGAGC TAGACGTTAA
Pa_50-2     CAGAGGTAGT GAAGACAAAT AACTAAATTC TGTCCTTTGG ATAGGATTTA AGGGATGAGC TAGACGTTAA
Pa_57-2     CAGAGGTAGT GAAGACAAAT AACTAAATTC TGTCCTTTGG ATAGGATTTA AGGGATGAGC TAGACGTTAA
Pa_57-3     CAGAGGTAGT GAAGACAAAT AACTAAATTC TGTCCTTTGG ATAGGATTTA AGGGATGAGC TAGACGTTAA
Pa_57-5     CAGAGGTAGT GAAGACAAAT AACTAAATTC TGTCCTTTGG ATAGGATTTA AGGGATGAGC TAGACGTTAA
Pa_62-19    CAGAGGTAGT GAAGACAAAT AACTAAATGC TGTCCACTGG ATAGCGTTTA AGGGATGAGC TAGACGTTAA
Pa_62-3     CAGAGGTAGT GAAGACAAAT AACTAAATGC TGTCCACTGG ATAGCGTTTA AGGGATGAGC TAGACGTTAA
Pa_63-2     CAGAGGTAGT GAAGACAAAT AACTAAACTC TGTCCACTGG ATAGAGTTTA AGGGATGAGC TAGACGTTAA
Pa_63-3     CAGAGGTAGT GAAGACAAAT AACTAAACTC TGTCCACTGG ATAGAGTTTA AGGGATGAGC TAGACGTTAA
Pa_63-4     CAGAGGTAGT GAAGACAAAT AACTAAACGC TGTCCACTGG ATAGTGTTTA AGGGATGAGC TAGACGTTAA
Pa_64-1     CAGAGGTAGT GAAGACAAAT AACTAAACTG TGTCCAATGG ACAGAGTTTA AGGGATGAGC TAGACGTTAA
Pa_64-2     CAGAGGTAGT GAAGACAAAT AACTAAACTG TGTCCAATGG ACAGAGTTTA AGGGATGAGC TAGACGTTAA
Pa_64-3     CAGAGGTAGT GAAGACAAAT AACTAAACTG TGTCCAATGG ACAGAGTTTA AGGGATGAGC TAGACGTTAA
Pa_64-4     CAGAGGTAGT GAAGACAAAT AACTAAACTG TGTCCAATGG ACAGAGTTTA AGGGATGAGC TAGACGTTAA
Pa_79-4     CAGAGGTAGT GAAGACAAAT AACTAAATGC TGTCCACTGG ATAGCGTTTA AGGGATGAGC TAGACGTTAA
```

```
             491
Bd_06-10     CACTCTAGCG AAAGCGATTG GAGGGCAAGT CTGGTGCCAG CAGCCGCGGT AATTCCAGCT CCAATAGTGT
Bd_09-1      CACTCTAGCG AAAGCGATTG GAGGGCAAGT CTGGTGCCAG CAGCCGCGGT AATTCCAGCT CCAATAGTGT
Bd_09-2      CACTCTAGCG AAAGCGATTG GAGGGCAAGT CTGGTGCCAG CAGCCGCGGT AATTCCAGCT CCAATAGTGT
Bd_09-3      CACTCTAGCG AAAGCGATTG GAGGGCAAGT CTGGTGCCAG CAGCCGCGGT AATTCCAGCT CCAATAGTGT
Bd_10-1      CACTCTAGCG AAAGCGATTG GAGGGCAAGT CTGGTGCCAG CAGCCGCGGT AATTCCAGCT CCAATAGTGT
Bd_10-2      CACTCTAGCG AAAGCGATTG GAGGGCAAGT CTGGTGCCAG CAGCCGCGGT AATTCCAGCT CCAATAGTGT
Bd_10-2b     CACTCTAGCG AAAGCGATTG GAGGGCAAGT CTGGTGCCAG CAGCCGCGGT AATTCCAGCT CCAATAGTGT
Bd_11-2      CACTCTAGCG AAAGCGATTG GAGGGCAAGT CTGGTGCCAG CAGCCGCGGT AATTCCAGCT CCAATAGTGT
Bd_11-6      CACTCTAGCG AAAGCGATTG GAGGGCAAGT CTGGTGCCAG CAGCCGCGGT AATTCCAGCT CCAATAGTGT
Bd_12-2      CACTCTAGCG AAAGCGATTG GAGGGCAAGT CTGGTGCCAG CAGCCGCGGT AATTCCAGCT CCAATAGTGT
Bd_13-1      CACTCTAGCG AAAGCGATTG GAGGGCAAGT CTGGTGCCAG CAGCCGCGGT AATTCCAGCT CCAATAGTGT
Bd_13-4      CACTCTAGCG AAAGCGATTG GAGGGCAAGT CTGGTGCCAG CAGCCGCGGT AATTCCAGCT CCAATAGTGT
Bd_13-5      CACTCTAGCG AAAGCGATTG GAGGGCAAGT CTGGTGCCAG CAGCCGCGGT AATTCCAGCT CCAATAGTGT
Bd_14-1      CACTCTAGCG AAAGCGATTG GAGGGCAAGT CTGGTGCCAG CAGCCGCGGT AATTCCAGCT CCAATAGTGT
Bd_14-2      CACTCTAGCG AAAGCGATTG GAGGGCAAGT CTGGTGCCAG CAGCCGCGGT AATTCCAGCT CCAATAGTGT
Bd_19-5      CACTCTAGCG AAAGCGATTG GAGGGCAAGT CTGGTGCCAG CAGCCGCGGT AATTCCAGCT CCAATAGTGT
Bd_19-6      CACTCTAGCG AAAGCGATTG GAGGGCAAGT CTGGTGCCAG CAGCCGCGGT AATTCCAGCT CCAATAGTGT
Bd_20-1      CACTCTAGCG AAAGCGATTG GAGGGCAAGT CTGGTGCCAG CAGCCGCGGT AATTCCAGCT CCAATAGTGT
Bd_20-2      CACTCTAGCG AAAGCGATTG GAGGGCAAGT CTGGTGCCAG CAGCCGCGGT AATTCCAGCT CCAATAGTGT
Bd_22-1      CACTCTAGCG AAAGCGATTG GAGGGCAAGT CTGGTGCCAG CAGCCGCGGT AATTCCAGCT CCAATAGTGT
Go_06-1      CACTCTAGCG AAAGCGATTG GAGGGCAAGT CTGGTGCCAG CAGCCGCGGT AATTCCAGCT CCAATAGTGT
Go_06-9      CACTCTAGCG AAAGCGATTG GAGGGCAAGT CTGGTGCCAG CAGCCGCGGT AATTCCAGCT CCAATAGTGT
Go_07-1      CACTCTAGCG AAAGCGATTG GAGGGCAAGT CTGGTGCCAG CAGCCGCGGT AATTCCAGCT CCAATAGTGT
Go_07-5      CACTCTAGCG AAAGCGATTG GAGGGCAAGT CTGGTGCCAG CAGCCGCGGT AATTCCAGCT CCAATAGTGT
Go_07-6      CACTCTAGCG AAAGCGATTG GAGGGCAAGT CTGGTGCCAG CAGCCGCGGT AATTCCAGCT CCAATAGTGT
Go_07-8      CACTCTAGCG AAAGCGATTG GAGGGCAAGT CTGGTGCCAG CAGCCGCGGT AATTCCAGCT CCAATAGTGT
Go_09-2      CACTCTAGCG AAAGCGATTG GAGGGCAAGT CTGGTGCCAG CAGCCGCGGT AATTCCAGCT CCAATAGTGT
Go_09-3      CACTCTAGCG AAAGCGATTG GAGGGCAAGT CTGGTGCCAG CAGCCGCGGT AATTCCAGCT CCAATAGTGT
Go_09-4      CACTCTAGCG AAAGCGATTG GAGGGCAAGT CTGGTGCCAG CAGCCGCGGT AATTCCAGCT CCAATAGTGT
Go_10-1      CACTCTAGCG AAAGCGATTG GAGGGCAAGT CTGGTGCCAG CAGCCGCGGT AATTCCAGCT CCAATAGTGT
Go_10-3      CACTCTAGCG AAAGCGATTG GAGGGCAAGT CTGGTGCCAG CAGCCGCGGT AATTCCAGCT CCAATAGTGT
Go_11-3      CACTCTAGCG AAAGCGATTG GAGGGCAAGT CTGGTGCCAG CAGCCGCGGT AATTCCAGCT CCAATAGTGT
Go_11-5      CACTCTAGCG AAAGCGATTG GAGGGCAAGT CTGGTGCCAG CAGCCGCGGT AATTCCAGCT CCAATAGTGT
Go_13-5      CACTCTAGCG AAAGCGATTG GAGGGCAAGT CTGGTGCCAG CAGCCGCGGT AATTCCAGCT CCAATAGTGT
Go_14-2      CACTCTAGCG AAAGCGATTG GAGGGCAAGT CTGGTGCCAG CAGCCGCGGT AATTCCAGCT CCAATAGTGT
Go_14-3      CACTCTAGCG AAAGCGATTG GAGGGCAAGT CTGGTGCCAG CAGCCGCGGT AATTCCAGCT CCAATAGTGT
Go_14-4      CACTCTAGCG AAAGCGATTG GAGGGCAAGT CTGGTGCCAG CAGCCGCGGT AATTCCAGCT CCAATAGTGT
Pa_06-2      TACTCTAGTG AAAGCGATTG GAGGGCAAGT CTGGTGCCAG CAGCCGCGGT AATTCCAGCT CCAATAGTGT
Pa_14-4      CACTCTAGCG AAAGCGATTG GAGGGCAAGT CTGGTGCCAG CAGCCGCGGT AATTCCAGCT CCAATAGTGT
Pa_14-6      CACTCTAGCG AAAGCGATTG GAGGGCAAGT CTGGTGCCAG CAGCCGCGGT AATTCCAGCT CCAATAGTGT
Pa_16-1      CACTCTAGCG AAAGCGATTG GAGGGCAAGT CTGGTGCCAG CAGCCGCGGT AATTCCAGCT CCAATAGTGT
Pa_19-1      CACTCTAGCG AAAGCGATTG GAGGGCAAGT CTGGTGCCAG CAGCCGCGGT AATTCCAGCT CCAATAGTGT
Pa_19-2      CACTCTAGCG AAAGCGATTG GAGGGCAAGT CTGGTGCCAG CAGCCGCGGT AATTCCAGCT CCAATAGTGT
Pa_19-3      CACTCTAGCG AAAGCGATTG GAGGGCAAGT CTGGTGCCAG CAGCCGCGGT AATTCCAGCT CCAATAGTGT
Pa_21-2      CACTCTAGCG AAAGCGATTG GAGGGCAAGT CTGGTGCCAG CAGCCGCGGT AATTCCAGCT CCAATAGTGT
Pa_22-3      CACTCTAGCG AAAGCGATTG GAGGGCAAGT CTGGTGCCAG CAGCCGCGGT AATTCCAGCT CCAATAGTGT
Pa_39-1      CACTCTAGCG AAAGCGATTG GAGGGCAAGT CTGGTGCCAG CAGCCGCGGT AATTCCAGCT CCAATAGTGT
Pa_39-5      CACTCTAGCG AAAGCGATTG GAGGGCAAGT CTGGTGCCAG CAGCCGCGGT AATTCCAGCT CCAATAGTGT
Pa_50-11     TACTCTAGTG AAAGCGATTG GAGGGCAAGT CTGGTGCCAG CAGCCGCGGT AATTCCAGCT CCAATAGTGT
Pa_50-12     TACTCTAGTG AAAGCGATTG GAGGGCAAGT CTGGTGCCAG CAGCCGCGGT AATTCCAGCT CCAATAGTGT
Pa_50-19     TACTCTAGTG AAAGCGATTG GAGGGCAAGT CTGGTGCCAG CAGCCGCGGT AATTCCAGCT CCAATAGTGT
Pa_50-2      TACTCTAGTG AAAGCGATTG GAGGGCAAGT CTGGTGCCAG CAGCCGCGGT AATTCCAGCT CCAATAGTGT
Pa_57-2      TACTCTAGTG AAAGCGATTG GAGGGCAAGT CTGGTGCCAG CAGCCGCGGT AATTCCAGCT CCAATAGTGT
Pa_57-3      TACTCTAGTG AAAGCGATTG GAGGGCAAGT CTGGTGCCAG CAGCCGCGGT AATTCCAGCT CCAATAGTGT
Pa_57-5      TACTCTAGTG AAAGCGATTG GAGGGCAAGT CTGGTGCCAG CAGCCGCGGT AATTCCAGCT CCAATAGTGT
Pa_62-19     CACTCTAGCG AAAGCGATTG GAGGGCAAGT CTGGTGCCAG CAGCCGCGGT AATTCCAGCT CCAATAGTGT
Pa_62-3      CACTCTAGCG AAAGCGATTG GAGGGCAAGT CTGGTGCCAG CAGCCGCGGT AATTCCAGCT CCAATAGTGT
Pa_63-2      CACTCTAGCG AAAGCGATTG GAGGGCAAGT CTGGTGCCAG CAGCCGCGGT AATTCCAGCT CCAATAGTGT
Pa_63-3      CACTCTAGCG AAAGCGATTG GAGGGCAAGT CTGGTGCCAG CAGCCGCGGT AATTCCAGCT CCAATAGTGT
Pa_63-4      CACTCTAGCG AAAGCGATTG GAGGGCAAGT CTGGTGCCAG CAGCCGCGGT AATTCCAGCT CCAATAGTGT
Pa_64-1      CACTCTAGCG AAAGCGATTG GAGGGCAAGT CTGGTGCCAG CAGCCGCGGT AATTCCAGCT CCAATAGTGT
Pa_64-2      CACTCTAGCG AAAGCGATTG GAGGGCAAGT CTGGTGCCAG CAGCCGCGGT AATTCCAGCT CCAATAGTGT
Pa_64-3      CACTCTAGCG AAAGCGATTG GAGGGCAAGT CTGGTGCCAG CAGCCGCGGT AATTCCAGCT CCAATAGTGT
Pa_64-4      CACTCTAGCG AAAGCGATTG GAGGGCAAGT CTGGTGCCAG CAGCCGCGGT AATTCCAGCT CCAATAGTGT
Pa_79-4      CACTCTAGCG AAAGCGATTG GAGGGCAAGT CTGGTGCCAG CAGCCGCGGT AATTCCAGCT CCAATAGTGT
```

```
           561
Bd_06-10   ATATTAAAGT TGTTGTGATT AAAAAGCTCG TAGTTGAATT TGACGAGGAC TTA-GGCTCT TCCGCTCTGC
Bd_09-1    ATATTAAAGT TGTTGTGATT AAAAAGCTCG TAGTTGAATT TGACGAGGAC TTA-GGCTCT TCCGCTCTGC
Bd_09-2    ATATTAAAGT TGTTGTGATT AAAAAGCTCG TAGTTGAATT TGACGAGGAC TTA-GGCTCT TCCGCTCTGC
Bd_09-3    ATATTAAAGT TGTTGTGATT AAAAAGCTCG TAGTTGAATT TGACCAGGAC TTA-GGCTCT TCCGCTCTGC
Bd_10-1    ATATTAAAGT TGTTGTGATT AAAAAGCTCG TAGTTGAATT TGACCAGGAC TTA-GGCTCT TCCGCTCTGC
Bd_10-2    ATATTAAAGT TGTTGTGATT AAAAAGCTCG TAGTTGAATT TGACGAGGAC TTA-GGCTCT TCCGCTCTGC
Bd_10-2b   ATATTAAAGT TGTTGTGATT AAAAAGCTCG TAGTTGAATT TGACGAGGAC TTA-GGCTCT TCCGCTCTGC
Bd_11-2    ATATTAAAGT TGTTGTGATT AAAAAGCTCG TAGTTGAATT TGACCAGGAC TTA-GGCTCT TCCGCTCTGC
Bd_11-6    ATATTAAAGT TGTTGTGATT AAAAAGCTCG TAGTTGAATT TGACGAGGAC TTA-GGCTCT TCCGCTCTGC
Bd_12-2    ATATTAAAGT TGTTGTGATT AAAAAGCTCG TAGTTGAATT TGACGAGGAC TTA-GGCTCT TCCGCTCTGC
Bd_13-1    ATATTAAAGT TGTTGTGATT AAAAAGCTCG TAGTTGAATT TGACGAGGAC TTA-GGCTCT TCCGCTCTGC
Bd_13-4    ATATTAAAGT TGTTGTGATT AAAAAGCTCG TAGTTGAATT TGACGAGGAC TTA-GGCTCT TCCGCTCTGC
Bd_13-5    ATATTAAAGT TGTTGTGATT AAAAAGCTCG TAGTTGAATT TGACGAGGAC TTA-GGCTCT TCCGCTCTGC
Bd_14-1    ATATTAAAGT TGTTGTGATT AAAAAGCTCG TAGTTGAATT TGACGAGGAC TTA-GGCTCT TCCGCTCTGC
Bd_14-2    ATATTAAAGT TGTTGTGATT AAAAAGCTCG TAGTTGAATT TGACGAGGAC TTA-GGCTCT TCCGCTCTGC
Bd_19-5    ATATTAAAGT TGTTGTGATT AAAAAGCTCG TAGTTGAATT TGACGAGGAC TTA-GGCTCT TCCGCTCTGC
Bd_19-6    ATATTAAAGT TGTTGTGATT AAAAAGCTCG TAGTTGAATT TGACGAGGAC TTA-GGCTCT TCCGCTCTGC
Bd_20-1    ATATTAAAGT TGTTGTGATT AAAAAGCTCG TAGTTGAATT TGACGAGGAC TTA-GGCTCT TCCGCTCTGC
Bd_20-2    ATATTAAAGT TGTTGTGATT AAAAAGCTCG TAGTTGAATT TGACCAGGAC TTA-GGCTCT TCCGCTCTGC
Bd_22-1    ATATTAAAGT TGTTGTGATT AAAAAGCTCG TAGTTGAATT TGACGAGGAC TTA-GGCTCT TCCGCTCTGC
Go_06-1    ATATTAAAGT TGTTGTGATT AAAAAGCTCG TAGTTGAATT AGACAAGAAC CCCCGGCCTC TGGGGCATGC
Go_06-9    ATATTAAAGT TGTTGTGATT AAAAAGCTCG TAGTTGAATT AGACAAGAAC CCCCGGCCTC TGGGGCATGC
Go_07-1    ATATTAAAGT TGTTGTGATT AAAAAGCTCG TAGTTGAATT AGACAAGAAC CCCCGGCCTC TGGGGCATGC
Go_07-5    ATATTAAAGT TGTTGTGATT AAAAAGCTCG TAGTTGAATT TGACAAGAAC CCCCGGCCTC TGGGGCATGC
Go_07-6    ATATTAAAGT TGTTGTGATT AAAAAGCTCG TAGTTGAATT TGACAAGAAC CCCCGGCCTC TGGGGCATGC
Go_07-8    ATATTAAAGT TGTTGTGATT AAAAAGCTCG TAGTTGAATT AGACAAGAAC CCCCGGCCTC TGGGGCATGC
Go_09-2    ATATTAAAGT TGTTGTGATT AAAAAGCTCG TAGTTGAATT TGACGAGGAC TTA-GGCTCT TCCGCTCTGC
Go_09-3    ATATTAAAGT TGTTGTGATT AAAAAGCTCG TAGTTGAATT TGACCAGGAC TTA-GGCTCT TCCGCTCTGC
Go_09-4    ATATTAAAGT TGTTGTGATT AAAAAGCTCG TAGTTGAATT TGACGAGGAC TTA-GGCTCT TCCGCTCTGC
Go_10-1    ATATTAAAGT TGTTGTGATT AAAAAGCTCG TAGTTGAATT AGACAAGAAC CCCCGGCCTC TGGGGCATGC
Go_10-3    ATATTAAAGT TGTTGTGATT AAAAAGCTCG TAGTTGAATT TGACAAGAAC CCCCGGCCTC TGGGGCATGC
Go_11-3    ATATTAAAGT TGTTGTGATT AAAAAGCTCG TAGTTGAATT AGACAAGAAC CCCCGGCCTC TGGGGCATGC
Go_11-5    ATATTAAAGT TGTTGTGATT AAAAAGCTCG TAGTTGAATT TGACAAGAAC CCCCGGCCTC TGGGGCATGC
Go_13-5    ATATTAAAGT TGTTGTGATT AAAAAGCTCG TAGTTGAATT AGACAAGAAC CCCCGGCCTC TGGGGCATGC
Go_14-2    ATATTAAAGT TGTTGTGATT AAAAAGCTCG TAGTTGAATT AGACAAGAAC CCCCGGCCTC TGGGGCATGC
Go_14-3    ATATTAAAGT TGTTGTGATT AAAAAGCTCG TAGTTGAATT AGACAAGAAC CCCCGGCCTC TGGGGCATGC
Go_14-4    ATATTAAAGT TGTTGTGATT AAAAAGCTCG TAGTTGAATT AGACAAGAAC CCCCGGCCTC TGGGGCATGC
Pa_06-2    ATATTAAAGT TGTTGTGATT AAAAAGCTCG TAGTTGAATT TGACATGGAC TCTTGGATCA TTCTCTTTTA
Pa_14-4    ATATTAAAGT TGTTGTGATT AAAAAGCTCG TAGTTGAATT TGACAAGGGC TCT-GGCCCA TCGGCACTGC
Pa_14-6    ATATTAAAGT TGTTGTGATT AAAAAGCTCG TAGTTGAATT TGACAAGGGC TCT-GGCCCA TCGGCACTGC
Pa_16-1    ATATTAAAGT TGTTGTGATT AAAAAGCTCG TAGTTGAAGC GAACGAGAAC TTT-GGCTTG TTGACTCTGC
Pa_19-1    ATATTAAAGT TGTTGTGATT AAAAAGCTCG TAGTTGAATA TGACAAGAAC TTT-GGCGCT GTTTCTCCCT
Pa_19-2    ATATTAAAGT TGTTGTGATT AAAAAGCTCG TAGTTGAATA TGACAAGAAC TTT-GGCGCT GTTTCTCCCT
Pa_19-3    ATATTAAAGT TGTTGTGATT AAAAAGCTCG TAGTTGAATA TGACAAGAAC TTT-GGCGCT GTTTCTCCCT
Pa_21-2    ATATTAAAGT TGTTGTGATT AAAAAGCTCG TAGTTGAATT TGACGAGAAC TTA-GGATCT TTTGCTCTGC
Pa_22-3    ATATTAAAGT TGTTGTGATT AAAAAGCTCG TAGTTGAATT TGACGAGAAC TTA-GGATCT TTTGCTCTGC
Pa_39-1    ATATTAAAGT TGTTGTGATT AAAAAGCTCG TAGTTGAATT TGACAAGGAC TCT-GGCCC- TCAGCACTGC
Pa_39-5    ATATTAAAGT TGTTGTGATT AAAAAGCTCG TAGTTGAATT TGACAAGGAC TCT-GGCCC- TCAGCACTGC
Pa_50-11   ATATTAAAGT TGTTGTGATT AAAAAGCTCG TAGTTGAATT TGACATGGAC TCTTGGATCA TTCTCTTTTA
Pa_50-12   ATATTAAAGT TGTTGTGATT AAAAAGCTCG TAGTTGAATT TGACATGGAC TCTTGGATCA TTCTCTTTTA
Pa_50-19   ATATTAAAGT TGTTGTGATT AAAAAGCTCG TAGTTGAATT TGACATGGAC TCTTGGATCA TTCTCTTTTA
Pa_50-2    ATATTAAAGT TGTTGTGATT AAAAAGCTCG TAGTTGAATT TGACATGGAC TCTTGGATCA TTCTCTTTTA
Pa_57-2    ATATTAAAGT TGTTGTGATT AAAAAGCTCG TAGTTGAATT TGACATGGAC TCTTGGATCA TTCTCTTTTA
Pa_57-3    ATATTAAAGT TGTTGTGATT AAAAAGCTCG TAGTTGAATT TGACATGGAC TCTTGGATCA TTCTCTTTTA
Pa_57-5    ATATTAAAGT TGTTGTGATT AAAAAGCTCG TAGTTGAATT TGACATGGAC TCTTGGATCA TTCTCTTTTA
Pa_62-19   ATATTAAAGT TGTTGTGATT AAAAAGCTCG TAGTTGAATT CGACAAGAAC TTT-GGCGCT GTTTCTCCCT
Pa_62-3    ATATTAAAGT TGTTGTGATT AAAAAGCTCG TAGTTGAATT CGACAAGAAC TTT-GGCGCT GTTTCTCCCT
Pa_63-2    ATATTAAAGT TGTTGTGATT AAAAAGCTCG TAGTTGAATT TGACGAGAAC TTA-GGATCT TTTGCTCTGC
Pa_63-3    ATATTAAAGT TGTTGTGATT AAAAAGCTCG TAGTTGAATT TGACGAGAAC TTA-GGATCT TTTGCTCTGC
Pa_63-4    ATATTAAAGT TGTTGTGATT AAAAAGCTCG TAGTTGAATT AGACAAGGAC TGT-GGTCC- CCAGCACTGC
Pa_64-1    ATATTAAAGT TGTTGTGATT AAAAAGCTCG TAGTTGAATT AGACGAGGAC TCT-GGCAAT GTGCCTCTGC
Pa_64-2    ATATTAAAGT TGTTGTGATT AAAAAGCTCG TAGTTGAATT AGACGAGGAC TCT-GGCAAT GTGCCTCTGC
Pa_64-3    ATATTAAAGT TGTTGTGATT AAAAAGCTCG TAGTTGAATT AGACGAGGAC TCT-GGCAAT GTGCCTCTGC
Pa_64-4    ATATTAAAGT TGTTGTGATT AAAAAGCTCG TAGTTGAATT AGACGAGGAC TCT-GGCAAT GTGCCTCTGC
Pa_79-4    ATATTAAAGT TGTTGTGATT AAAAAGCTCG TAGTTGAATT CGACAGGACT TTG----TTT CAGCCGCTAC
```

```
           631
Bd_06-10   ATGCAGAGAG GAGGAGGCCT TCACTAGTCC TAAACACGCT GCA--CTTTG CCTCTTCGGA GGCGGGTGGA
Bd_09-1    ATGCAGAGAG GAGGAGGCCT TCACTAGTCC TAAACACGCT GCA--CTTTG CCTCTTCGGA GGCGGGTGGA
Bd_09-2    ATGCAGAGAG GAGGAGGCCT TCACTAGTCC TAAACACGCT GCA--CTTTG CCTCTTCGGA GGCGGGTGGA
Bd_09-3    ACGCAGAGAG GAGGAGGCCT TCACTAGTCC TAAACACGCT GCA--CTTTG CCTCTTCGGA GGCGGGTGGA
Bd_10-1    ACGCAGAGAG GAGGAGGCCT TCACTAGTCC TAAACACGCT GCA--CTTTG CCTCTTCGGA GGCGGGTGGA
Bd_10-2    ATGCAGAGAG GAGGAGGCCT TCACTAGTCC TAAACACGCT GCA--CTTTG CCTCTTCGGA GGCGGGTGGA
Bd_10-2b   ATGCAGAGAG GAGGAGGCCT TCACTAGTCC TAAACACGCT GCA--CTTTG CCTCTTCGGA GGCGGGTGGA
Bd_11-2    ACGCAGAGAG GAGGAGGCCT TCACTAGTCC TAAACACGCT GCA--CTTTG CCTCTTCGGA GGCGGGTGGA
Bd_11-6    ATGCAGAGAG GAGGAGGCCT TCACTAGTCC TAAACACGCT GCA--CTTTG CCTCTTCGGA GGCGGGTGGA
Bd_12-2    ACGCAGAGGG GAGGAGGCCT TCACTAGTCC TAAACACGCT GCA--CTTTG CCTCTTCGGA GGCGGGTGAA
Bd_13-1    ATGCAGAGAG GAGGAGGCCT TCACTAGTCC TAAACACGCT GCA--CTTTG CCTCTTCGGA GGCGGGTGGA
Bd_13-4    ATGCAGAGAG GAGGAGGCCT TCACTAGTCC TAAACACGCT GCA--CTTTG CCTCTTCGGA GGCGGGTGGA
Bd_13-5    ATGCAGAGAG GAGGAGGCCT TCACTAGTCC TAAACACGCT GCA--CTTTG CCTCTTCGGA GGCGGGTGGA
Bd_14-1    ATGCAGAGAG GAGGAGGCCT TCACTAGTCC TAAACACGCT GCA--CTTTG CCTCTTCGGA GGCGGGTGGA
Bd_14-2    ATGCAGAGAG GAGGAGGCCT TCACTAGTCC TAAACACGCT GCA--CTTTG CCTCTTCGGA GGCGGGTGGA
Bd_19-5    ATGCAGAGAG GAGGAGGCCT TCACTAGTCC TAAACACGCT GCA--CTTTG CCTCTTCGGA GGCGGGTGGA
Bd_19-6    ATGCAGAGAG GAGGAGGCCT TCACTAGTCC TAAACACGCT GCA--CTTTG CCTCTTCGGA GGCGGGTGGA
Bd_20-1    ATGCAGAGAG GAGGAGGCCT TCACTAGTCC TAAACACGCT GCA--CTTTG CCTCTTCGGA GGCGGGTGGA
Bd_20-2    ACGCAGAGAG GAGGAGGCCT TCACTAGTCC TAAACACGCT GCA--CTTTG CCTCTTCGGA GGCGGGTGGA
Bd_22-1    ACGCAGAGGG GAGGAGGCCT TCACTAGTCC TAAACACGCT GCA--CTTTG CCTCTTCGGA GGCGGGTGAA
Go_06-1    AAGCATGTCA CAGGGCGCCT TCAATGGTTC TAAACACTCA CTG--CAGCT CCTCTCCGGA GGACTGCTGT
Go_06-9    AAGCATGTCA CAGGGCGCCT TCAATGGTTC TAAACACTCA CTG--TAGCC TCTCTCCGGA GAGCTATCGT
Go_07-1    AAGCATGTCA CAGGGCGCCT TCAATGGTTC TAAACACTCA CTG--CAGCT CCTCTCCGGA GGACTGCTGT
Go_07-5    AAGCATGTCA CAGGGCGCCT TCAATGGTTC TAAACACTCA CTG--CAGCT CCTCTCCGGA GGACTGCTGT
Go_07-6    AAGCATGTCA CAGGGCGCCT TCAATGGTTC TAAACACTCA CTG--TAGCC TCTCTCCGGA GAGCTATCGT
Go_07-8    AAGCATGTCA CAGGGCGCCT TCAATGGTTC TAAACACTCA CTG--CAGCT CCTCTCCGGA GGACTGCTGT
Go_09-2    ACGCAGAGGG GAGGAGGCCT TCACTAGTCC TAAACACGCT GCA--CTTTG CCTCTTCGGA GGCGGGTGAA
Go_09-3    ACGCAGAGAG GAGGAGGCCT TCACTAGTCC TAAACACGCT GCA--CTTTG CCTCTTCGGA GGCGGGTGGA
Go_09-4    ATGCAGAGAG GAGGAGGCCT TCACTAGTCC TAAACACGCT GCA--CTTTG CCTCTTCGGA GGCGGGTGGA
Go_10-1    AAGCATGTCA CAGGGCGCCT TCAATGGTTC TAAACACTCA CTG--CAGCT CCTCTCCGGA GGACTGCTGT
Go_10-3    AAGCATGTCA CAGGGCGCCT TCAATGGTTC TAAACACTCA CTG--CAGCT CCTCTCCGGA GGACTGCTGT
Go_11-3    AAGCATGTCA CAGGGCGCCT TCAATGGTTC TAAACACTCA CTG--CAGTC TCTCTCCGGA GAGCTGCTGT
Go_11-5    AAGCATGTCA CAGGGCGCCT TCAATGGTTC TAAACACTCA CTG--CAGCT CCTCTCCGGA GGACTGCTGT
Go_13-5    AAGCATGTCA CAGGGCGCCT TCAATGGTTC TAAACACTCA CTG--CAGCT CCTCTCCGGA GGACTGCTGT
Go_14-2    AAGCATGTCA CAGGGCGCCT TCAATGGTTC TAAACACTCA CTG--CAGCT CCTCTCCGGA GGACTGCTGT
Go_14-3    AAGCATGTCA CAGGGCGCCT TCAATGGTTC TAAACACTCA CTG--CAGCT CCTCTCCGGA GGACTGCTGT
Go_14-4    AAGCATGTCA CAGGGCGCCT TCAATGGTTC TAAACACTCA CTG--CAGCT CCTCTCCGGA GGACTGCTGT
Pa_06-2    TCCAAAAGAG AGCGATTCCT TCCACAGTCC TCAACACCTT TCATGTTGCT TCTCTCGAGA GGCTCATGGA
Pa_14-4    ACGCAGTGGT GGTGGGACCT TCCGTAGTCC TAAACACCTC AGG--TGCTG CTCTTCGGAG CGGTAACTGG
Pa_14-6    ACGCAGTGGT GGTGGGACCT TCCGTAGTCC TAAACACCTC AGG--TGCTG CTCTTCGGAG CGGTAACTGG
Pa_16-1    AAGCAGAGAC AGCAC-GTCA GTGATAGTTC TAAACACCCC AGA--GCACG CTCTTCGGAG TGTGTTTTGG
Pa_19-1    CGGGAGCGAC AGTG--GTCA AGTATAGTTC TAAACACCTT CAA--TCACT CTTTTCGGAG GGTGGCTGGA
Pa_19-2    CGGGAGCGAC AGCG--GTCA AGTATAGTTC TAAACACCTT CAA--TCACT CTTTTCGGAG GGTGGCTGGA
Pa_19-3    CGGGAGCGAC AGTG--GTCA AGTATAGTTC TAAACACCTT CAA--TCACT CTTTTCGGAG GGTGGCTGGA
Pa_21-2    ACGCAGAGGA GAAGAGGCCT TCAATAGTTC TAAACAAGCT GCA--CTCTG CCTCTTCGGA GACAGGTGAA
Pa_22-3    ACGCAGAGGA GAAGAGGCCT TCAATAGTTC TAAACAAGCT GCA--CTCTG CCTCTTCGGA GACAGGTGAA
Pa_39-1    TTGCAGTTAC TGAGGGACCT TCCGTAGTTC TAAACACCTC ATC--TACTG CTTTTCGGAG CAGTAAGTGA
Pa_39-5    TTGCAGTTAC TGAGGGACCT TCCGTAGTTC TAAACACCTC ATC--TACTG CTTTTCGGAG CAGTAAGTGA
Pa_50-11   TCCAAAAGAG AGCGATTCCT TCCACAGTCC TCAACACCTT TCATGTTGCT TCTCTCGAGA GGCTCATGGA
Pa_50-12   TCCAAAAGAG AGCGATTCCT TCCACAGTCC TCAACACCTT TCATGTTGCT TCTCTCGAGA GGCTCATGGA
Pa_50-19   TCCAAAAGAG AGCGATTCCT TCCACAGTCC TCAACACCTT TCATGTTGCT TCTCTCGAGA GGCTCATGGA
Pa_50-2    TCCAAAAGAG AGCGATTCCT TCCACAGTCC TCAACACCTT TCATGTTGCT TCTCTCGAGA GGCTCATGGA
Pa_57-2    TCCAAAAGAG AGCGATTCCT TCCACAGTCC TCAACACCTT TCATGTTGCT TCTCTCGAGA GGCTCATGGA
Pa_57-3    TCCAAAAGAG AGCGATTCCT TCCACAGTCC TCAACACCTT TCATGTTGCT TCTCTCGAGA GGCTCATGGA
Pa_57-5    TCCAAAAGAG AGCGATTCCT TCCACAGTCC TCAACACCTT TCATGTTGCT TCTCTCGAGA GGCTCATGGA
Pa_62-19   CGGGAGCGAC AGTG--GTCA AGTATAGTTC TAAACACCTT CAG--TCACT CTTTTCGGAG GGTGAGTGGA
Pa_62-3    CGGGAGCGAC AGTG--GTCA AGTATAGTTC TAAACACCTT CAG--TCACT CTTTTCGGAG GGTGAGTGGA
Pa_63-2    ACGCAGAGCG GAAGAGGCCT TCAATAGTTC TAAAGAAGCT GCA--CTCTG CCTCTTCTGA GACAGGTGAA
Pa_63-3    ACGCAGAGCG GAAGAGGCCT TCAATAGTTC TAAAGAAGCT GCA--CTCTG CCTCTTCTGA GACAGGTGAA
Pa_63-4    ACGCAGTGCC TGAGG-ACCT TCAATAGTTC TAAACACCTC TGA--TACTG CTCTTCGGAG CCGTTTCCGA
Pa_64-1    TCGCAGAGAT GCAGC-GTCA AACACGGTCC TAAACACCTG CGC--GCGTT CTCTTCGGAG GGCGCACGTG
Pa_64-2    TCGCAGAGAT GCAGC-GTCA AACACGGTCC TAAACACCTG CGC--GCGTT CTCTTCGGAG GGCGCACGTG
Pa_64-3    TCGCAGAGAT GCAGC-GTCA AACACGGTCC TAAACACCTG CGC--GCGTT CTCTTCGGAG GGCGCACGTG
Pa_64-4    TCGCAGAGAT GCAGC-GTCA AACACGGTCC TAAACACCTG CGC--GCGTT CTCTTCGGAG GGCGCACGTG
Pa_79-4    TTGTAGCGAG TTGAG-AACA AGTACAGCCC TAAACACCTT CAC--ACTGT CTCTTCGGAG ATGGTGTGGA
```

```
            701
Bd_06-10    GATATTTACT TTGAATAAAT ATGAGTGTTT AAAGCAAAGC ATTAGCTAAT TAAATATGAA GCATGGGATA
Bd_09-1     GATATTTACT TTGAATAAAT ATGAGTGTTT AAAGCAAAGC ATTAGCTAAT TAAATATGAA GCATGGGATA
Bd_09-2     GATATTTACT TTGAATAAAT ATGAGTGTTT AAAGCAAAGC ATTAGCTAAT TAAATATGAA GCATGGGATA
Bd_09-3     GATATTTACT TTGAATAAAT ATGAGTGTTT AAAGCAAAGC ATTAGCTAAT TAAATATGAA GCATGGGATA
Bd_10-1     GATATTTACT TTGAATAAAT ATGAGTGTTT AAAGCAAAGC ATTAGCTAAT TAAATATGAA GCATGGGATA
Bd_10-2     GATATTTACT TTGAATAAAT ATGAGTGTTT AAAGCAAAGC ATTAGCTAAT TAAATATGAA GCATGGGATA
Bd_10-2b    GATATTTACT TTGAATAAAT ATGAGTGTTT AAAGCAAAGC ATTAGCTAAT TAAATATGAA GCATGGGATA
Bd_11-2     GATATTTACT TTGAATAAAT ATGAGTGTTT AAAGCAAAGC ATTAGCTAAT TAAATATGAA GCATGGGATA
Bd_11-6     GATATTTACT TTGAATAAAT ATGAGTGTTT AAAGCAAAGC ATTAGCTAAT TAAATATGAA GCATGGGATA
Bd_12-2     GATATTTACT TTGAATAAAT ATGAGTGTTT AAAGCAAAGC ATTAGCTAAT TAAATATGAA GCATGGGATA
Bd_13-1     GATATTTACT TTGAATAAAT ATGAGTGTTT AAAGCAAAGC ATTAGCTAAT TAAATATGAA GCATGGGATA
Bd_13-4     GATATTTACT TTGAATAAAT ATGAGTGTTT AAAGCAAAGC ATTAGCTAAT TAAATATGAA GCATGGGATA
Bd_13-5     GATATTTACT TTGAATAAAT ATGAGTGTTT AAAGCAAAGC ATTAGCTAAT TAAATATGAA GCATGGGATA
Bd_14-1     GATATTTACT TTGAATAAAT ATGAGTGTTT AAAGCAAAGC ATTAGCTAAT TAAATATGAA GCATGGGATA
Bd_14-2     GATATTTACT TTGAATAAAT ATGAGTGTTT AAAGCAAAGC ATTAGCTAAT TAAATATGAA GCATGGGATA
Bd_19-5     GATATTTACT TTGAATAAAT ATGAGTGTTT AAAGCAAAGC ATTAGCTAAT TAAATATGAA GCATGGGATA
Bd_19-6     GATATTTACT TTGAATAAAT ATGAGTGTTT AAAGCAAAGC ATTAGCTAAT TAAATATGAA GCATGGGATA
Bd_20-1     GATATTTACT TTGAATAAAT ATGAGTGTTT AAAGCAAAGC ATTAGCTAAT TAAATATGAA GCATGGGATA
Bd_20-2     GATATTTACT TTGAATAAAT ATGAGTGTTT AAAGCAAAGC ATTAGCTAAT TAAATATGAA GCATGGGATA
Bd_22-1     GATATTTACT TTGAATAAAT ATGAGTGTTT AAAGCAAAGC ATTAGCTAAT TAAATATGAA GCATGGGATA
Go_06-1     GATATTTACT TTGAATAAAT ACGAGTGTTT AAAGCAAAGC AATAGCTAAT GCAGTATCAA GCATGGGATA
Go_06-9     GATATTTACT TTGAATAAAT ACGAGTGTTT AAAGCAAAGC AATAGCTAAT GCAGTATCAA GCATGGGATA
Go_07-1     GATATTTACT TTGAATAAAT ACGAGTGTTT AAAGCAAAGC AATAGCTAAT GCAGTATCAA GCATGGGATA
Go_07-5     GATATTTACT TTGAATAAAT ACGAGTGTTT AAAGCAAAGC AATAGCTAAT GCAGTATCAA GCATGGGATA
Go_07-6     GATATTTACT TTGAATAAAT ACGAGTGTTT AAAGCAAAGC AATAGCTAAT GCAGTATCAA GCATGGGATA
Go_07-8     GATATTTACT TTGAATAAAT ACGAGTGTTT AAAGCAAAGC AATAGCTAAT GCAGTATCAA GCATGGGATA
Go_09-2     GATATTTACT TTGAATAAAT ATGAGTGTTT AAAGCAAAGC ATTAGCTAAT TAAATATGAA GCATGGGATA
Go_09-3     GATATTTACT TTGAATAAAT ATGAGTGTTT AAAGCAAAGC ATTAGCTAAT TAAATATGAA GCATGGGATA
Go_09-4     GATATTTACT TTGAATAAAT ATGAGTGTTT AAAGCAAAGC ATTAGCTAAT TAAATATGAA GCATGGGATA
Go_10-1     GATATTTACT TTGAATAAAT ACGAGTGTTT AAAGCAAAGC AATAGCTAAT GCAGTATCAA GCATGGGATA
Go_10-3     GATATTTACT TTGAATAAAT ACGAGTGTTT AAAGCAAAGC AATAGCTAAT GCAGTATCAA GCATGGGATA
Go_11-3     GATATTTACT TTGAATAAAT ACGAGTGTTT AAAGCAAAGC AATAGCTAAT GCAGTATCAA GCATGGGATA
Go_11-5     GATATTTACT TTGAATAAAT ACGAGTGTTT AAAGCAAAGC AATAGCTAAT GCAGTATCAA GCATGGGATA
Go_13-5     GATATTTACT TTGAATAAAT ACGAGTGTTT AAAGCAAAGC AATAGCTAAT GCAGTATCAA GCATGGGATA
Go_14-2     GATATTTACT TTGAATAAAT ACGAGTGTTT AAAGCAAAGC AATAGCTAAT GCAGTATCAA GCATGGGATA
Go_14-3     GATATTTACT TTGAATAAAT ACGAGTGTTT AAAGCAAAGC AATAGCTAAT GCAGTATCAA GCATGGGATA
Go_14-4     GATATTTACT TTGAATAAAT ACGAGTGTTT AAAGCAAAGC AATAGCTAAT GCAGTATCAA GCATGGGATA
Pa_06-2     GATATTTACT TTGAATAAAT ATGAGTGTTT AAAGCAAAGC ATTTGCTAAT TAAATATGAA GCATGGGATA
Pa_14-4     GATATTTACT TTGAATAAAT ATGAGTGTTT AAAGCAAAGC AATAGCTAAT AGAATATGAA GCATGGGATA
Pa_14-6     GATATTTACT TTGAATAAAT ATGAGTGTTT AAAGCAAAGC AATAGCTAAT AGAATATGAA GCATGGGATA
Pa_16-1     GATATTTACT TTGAATAAAT ATGAGTGTTT AAAGCAAAGC AAAAGCTAAT AGAATATGAA GCATGGGATA
Pa_19-1     GATATTTACT TTGAATAAAT ATGAGTGTTT AAAGCAAAGC AAAAGCTAAT AAAATATGAA GCATGGGATA
Pa_19-2     GATATTTACT TTGAATAAAT ATGAGTGTTT AAAGCAAAGC AAAAGCTAAT AAAATATGAA GCATGGGATA
Pa_19-3     GATATTTACT TTGAATAAAT ATGAGTGTTT AAAGCAAAGC AAAAGCTAAT AAAATATGAA GCATGGGATA
Pa_21-2     GATATTTACT TTGAATAAAT ATGAGTGTTT AAAGCAAAGC ATTAGCTAAT TAAATATGAA GCATGGGATA
Pa_22-3     GATATTTACT TTGAATAAAT ATGAGTGTTT AAAGCAAAGC ATTAGCTAAT TAAATATGAA GCATGGGATA
Pa_39-1     GATATTTACT TTGAATAAAT ATGAGTGTTT AAAGCAAAGC AATAGCTAAC AGAATCTGAA GCATGGGATA
Pa_39-5     GATATTTACT TTGAATAAAT ATGAGTGTTT AAAGCAAAGC AATAGCTAAC AGAATCTGAA GCATGGGATA
Pa_50-11    GATATTTACT TTGAATAAAT ATGAGTGTTT AAAGCAAAGC ATTTGCTAAT TAAATATGAA GCATGGGATA
Pa_50-12    GATATTTACT TTGAATAAAT ATGAGTGTTT AAAGCAAAGC ATTTGCTAAT TAAATATGAA GCATGGGATA
Pa_50-19    GATATTTACT TTGAATAAAT ATGAGTGTTT AAAGCAAAGC ATTTGCTAAT TAAATATGAA GCATGGGATA
Pa_50-2     GATATTTACT TTGAATAAAT ATGAGTGTTT AAAGCAAAGC ATTTGCTAAT TAAATATGAA GCATGGGATA
Pa_57-2     GATATTTACT TTGAATAAAT ATGAGTGTTT AAAGCAAAGC ATTTGCTAAT TAAATATGAA GCATGGGATA
Pa_57-3     GATATTTACT TTGAATAAAT ATGAGTGTTT AAAGCAAAGC ATTTGCTAAT TAAATATGAA GCATGGGATA
Pa_57-5     GATATTTACT TTGAATAAAT ATGAGTGTTT AAAGCAAAGC ATTTGCTAAT TAAATATGAA GCATGGGATA
Pa_62-19    GATATTTACT TTGAATAAAT ATGAGTGTTT AAAGCAAAGC AAAAGCTAAT AAAATATGAA GCATGGGATA
Pa_62-3     GATATTTACT TTGAATAAAT ATGAGTGTTT AAAGCAAAGC AAAAGCTAAT AAAATATGAA GCATGGGATA
Pa_63-2     GATATTTACT TTGAATAAAT ATGAGTGTTT AAAGCAAAGC ATTAGCTAAT TAAATATGAA GCATGGGATA
Pa_63-3     GATATTTACT TTGAATAAAT ATGAGTGTTT AAAGCAAAGC ATTAGCTAAT TAAATATGAA GCATGGGATA
Pa_63-4     GATATTTACT TTGAATAAAT ATGAGTGTTT AAAGCAAAGC AATAGCTAAT AGAATCTGAA GCATGGGATA
Pa_64-1     GATATTTACT TTGAATAAAT ATGAGTGTTT AAAGCAAAGC AATAGCTAAT AGAATATGAA GCATGGGATA
Pa_64-2     GATATTTACT TTGAATAAAT ATGAGTGTTT AAAGCAAAGC AATAGCTAAT AGAATATGAA GCATGGGATA
Pa_64-3     GATATTTACT TTGAATAAAT ATGAGTGTTT AAAGCAAAGC AATAGCTAAT AGAATATGAA GCATGGGATA
Pa_64-4     GATATTTACT TTGAATAAAT ATGAATGTTT AAAGCAAAGC AATAGCTAAT AGAATATGAA GCATGGGATA
Pa_79-4     GATATTTACT TTGAATAAAT ATGAATGTTT AAAGCAAAGC AAAAGCTAAT AGAATATGAA GCATGGGATA
```

```
              771
Bd_06-10  ATAGAGAGGA GATACTTACT T--ACGCACT ---ATGTGTT ACG-GTGGTA TTTCTAGATA AGGATTAATA
Bd_09-1   ATAGAGAGGA GATACTTACT T--ACGCACT ---ATGTGTT ACG-GTGGTA TTTCTAGATA AGGATTAATA
Bd_09-2   ATAGAGAGGA GATACTTACT T--ACGCACT ---ATGTGTT ACG-GTGGTA TTTCTAGATA AGGATTAATA
Bd_09-3   ATAGAGAGGA GATACTTACT TGCGCGCACT ---ATGTGTT ACG-GTGGTA TTTCTAGATA AGGATTAATA
Bd_10-1   ATAGAGAGGA GATACTTACT TGCGCGCACT ---ATGTGTT ACG-GTGGTA TTTCTAGATA AGGATTAATA
Bd_10-2   ATAGAGAGGA GATACTTACT T--ACGCACT ---ATGTGTT ACG-GTGGTA TTTCTAGATA AGGATTAATA
Bd_10-2b  ATAGAGAGGA GATACTTACT T--ACGCACT ---ATGTGTT ACA-GTGGTA TTTCTAGATA AGGATTAATA
Bd_11-2   ATAGAGAGGA GATACTTACT T--ACGCACT ---ATGTGTT ACG-GTGGTA TTTCTAGATA AGGATTAATA
Bd_11-6   ATAGAGAGGA GATACTTACT T--ACGCACT ---ATGTGAT ACG-GTGGTA TTTCTAGATA AGGATTAATA
Bd_12-2   ATAGAGAGGA GATATTTACT T--ACGCACT ---ATGTGTT ACG-GTGGTA TTTCTAGATA AGGATTAATA
Bd_13-1   ATAGAGAGGA GATACTTACT T--ACGCACT ---ATGTGTT ACA-GTGGTA TTTCTAGATA AGGATTAATA
Bd_13-4   ATAGAGAGGA GATACTTACT T--ACGCACT ---ATGTGTT ACG-GTGGTA TTTCTAGATA AGGATTAATA
Bd_13-5   ATAGAGAGGA GATACTTACT T--ACGCACT ---ATGTGAT ACG-GTGGTA TTTCTAGATA AGGATTAATA
Bd_14-1   ATAGAGAGGA GATACTTACT T--ACGCACT ---ATGTGTT ACG-GTGGTA TTTCTAGATA AGGATTAATA
Bd_14-2   ATAGAGAGGA GATACTTACT T--ACGCACT ---ATGTGTT ACG-GTGGTA TTTCTAGATA AGGATTAATA
Bd_19-5   ATAGAGAGGA GATACTTACT T--ACGCACT ---ATGTGAT ACG-GTGGTA TTTCTAGATA AGGATTAATA
Bd_19-6   ATAGAGAGGA GATACTTACT T--ACGCACT ---ATGTGAT ACG-GTGGTA TTTCTAGATA AGGATTAATA
Bd_20-1   ATAGAGAGGA GATACTTACT T--ACGCACT ---ATGTGTT ACG-GTGGTA TTTCTAGATA AGGATTAATA
Bd_20-2   ATAGAGAGGA GATACTTACT TGCGCGCACT ---ATGTGTT ACG-GTGGTA TTTCTAGATA AGGATTAATA
Bd_22-1   ATAGAGAGGA GATACTTACT T--ACGCACT ---ATGTGTT ACG-GTGGTA TTTCTAGATA AGGATTAATA
Go_06-1   AAAACGAGGA GATTGTTGCC T--GCGCACA ---CTGTGCA AGG-GTGCAA TGTCTAGATA AGGATTAATA
Go_06-9   AAAACGAGGA GATTGTTGCC T--GCGCACA ---CTGTGCA AGG-GTGCAA TGTCTAGATA AGGATTAATA
Go_07-1   AAAACGAGGA GATTGTTGCC T--GCGCACA ---CTGTGCA AGG-GCGCAA TGTCTAGATA AGGATTAATA
Go_07-5   AAAACGAGGA GATTGTTGCC T--GCGCACA ---CTGTGCA AGG-GCGCAA TGTCTAGATA AGGATTAATA
Go_07-6   AAAACGAGGA GATTGTTGCC T--GCGCACA ---CTGTGCA AGG-GTGCAA TGTCTAGATA AGGATTAATA
Go_07-8   AAAACGAGGA GATTGTTGCC T--GCGCACA ---CTGTGCA AGG-GTGCAA TGTCTAGATA AGGATTAATA
Go_09-2   ATAGAGAGGA GATACTTACT T--ACGCACT ---ATGTGTT ACG-GTGGTA TTTCTAGATA AGGATTAATA
Go_09-3   ATACAGAGGA GATACTTACT T--ACGCACT ---ATGTGTT ACG-GTGGTA TTTCTAGATA AGGATTAATA
Go_09-4   ATAGAGAGGA GATACTTACT T--ACGCACT ---ATGTGTT ACG-GTGGTA TTTCTAGATA AGGATTAATA
Go_10-1   AAAACGAGGA GATTGTTGCC T--GCGCACA ---CTGTGCA AGG-GTGCAA TGTCTAGATA AGGATTAATA
Go_10-3   AAAACGAGGA GATTGTTGCC T--GCGCACA ---CTGTGCA AGG-GCGCAA TGTCTAGATA AGGATTAATA
Go_11-3   AAAACGAGGA GATTGTTGCC T--GCGCACA ---CTGTGCA AGG-GCGCAA TGTCTAGATA AGGATTAATA
Go_11-5   AAAACGAGGA GATTGTTGCC T--GCGCACA ---CTGTGCA AGG-GCGCAA TGTCTAGATA AGGATTAATA
Go_13-5   AAAACGAGGA GATTGTTGCC T--GCGCACA ---CTGTGCA AGG-GCGCAA TGTCTAGATA AGGATTAATA
Go_14-2   AAAACGAGGA GATTGTTGCC T--GCGCACA ---CTGTGCA AGG-GCGCAA TGTCTAGATA AGGATTAATA
Go_14-3   AAAACGAGGA GATTGTTGCC T--GCGCACA ---CTGTGCA AGG-GCGCAA TGTCTAGATA AGGATTAATA
Go_14-4   AAAACGAGGA GATTGTTGCC T--GCGCACA ---CTGTGCA AGG-GCGCAA TGTCTAGATA AGGATTAATA
Pa_06-2   ATCATGAGGA GATACATTTC A-TTTTCACA CTTGTGAAAC ATG-ATTGTA TTTCTAGATC AAGATTAAGA
Pa_14-4   ATAGAGAGGA GATGGACATC C--AAGCACG ---ATGTGTG AGG-ATACCA TTTCTAGATA AGGATTAATA
Pa_14-6   ATAGAGAGGA GATGGACATC C--AAGCACG ---ATGTGTG AGG-ATACCA TTTCTAGATA AGGATTAATA
Pa_16-1   ATAGAGAGGA GATGCCATTG C-TGCGCACA ---GAGTGCA AGG-CAGGCA TTTCTAGAGA AGGATTAATA
Pa_19-1   ATAGAGAGGA GATACCCATC C-CAACCACA ---TTGTGGG TGG-CGGGTA TTTCTAGATA AGGATTAATA
Pa_19-2   ATAGAGAGGA GATACCCATC C-CAACCACA ---TTGTGGG TGG-CGGGTA TTTCTAGATA AGGATTAATA
Pa_19-3   ATAGAGAGGA GATACCCATC C-CAACCACA ---TTGTGGG TGG-CGGGTA TTTCTAGATA AGGATTAATA
Pa_21-2   ATAGAGAGGA GATTTCAATC TGCGTACAGA ---TTGTACA AGG-AAGGAA TTTCTATATA AGGATTAATA
Pa_22-3   ATAGAGAGGA GATTTCAATC TGCGTACAGA ---TTGTACA AGG-AAGGAA TTTCTATATA AGGATTAATA
Pa_39-1   ATAGTGAGGA GATGATGATC C--GAGCACA ---ATGTGTG AGGNAAGTCA TTTCTAGATA AGGATTAATA
Pa_39-5   ATAGTGAGGA GATGATGATC C--GAGCACA ---ATGTGTG AGG-AAGTCA TTTCTAGATA AGGATTAATA
Pa_50-11  ATCCTGAGGA GATACATTTC A-TTTTCACA CTTGTGAAAC ATG-ATTGTA TTTCTAGATC AAGATTAAGA
Pa_50-12  ATCATGAGGA GATACATTTC A-TTTTCACA CTTGTGAAAC ATG-ATTGTA TTTCTAGATC AAGATTAAGA
Pa_50-19  ATCATGAGGA GATACATTTC A-TTTTCACA CTTGTGAAAC ATG-ATTGTA TTTCTAGATC AAGATTAAGA
Pa_50-2   ATCATGAGGA GATACATTTC A-TTTTCACA CTTGTGAAAC ATG-ATTGGA TTTCTAGATC AAGATTAAGA
Pa_57-2   ATCATGAGGA GATACATTTC A-TTTTCACA CTTGTGAATC ATG-ATTGTA TTTCTAGATC AAGATTAAGA
Pa_57-3   ATCATGAGGA GATACATTTC A-TTTTCACA CTTGTGAATC ATG-ATTGTA TTTCTAGATC AAGATTAAGA
Pa_57-5   ATCATGAGGA GATACATTTC A-TTTTCACA CTTGTGAATC ATG-ATTGGA TTTCTAGATC AAGATTAAGA
Pa_62-19  ATAGAGAGGA GATACCCACA C-CAACCACA ---TTGTGAT TGG-CGGGTA TTTCTAGATA AGGATTAATA
Pa_62-3   ATAGAGAGGA GATACCCACA C-CAACCACA ---TTGTGAT TGG-CGGGTA TTTCTAGATA AGGATTAATA
Pa_63-2   ATAGAGAGGA GATTTCAATC TGCGTACGAT ---TTGTACA AAG-ATGGAA TTTCTATATA AGGATTAATA
Pa_63-3   ATAGAGAGGA GATTTCAATC TGCGTACGAT ---TTGTACA AAG-ATGGAA TTTCTATATA AGGATTAATA
Pa_63-4   ATAGTGAGGA GATGACGCTC C--GAGCACT ---TTGTGTG AGG-AGGTCA TTTCTAGATA AGGATTAATA
Pa_64-1   ATAGAGAGGA GATGCCATTT C-CAAGCACA ---GTGTGTG AGG-AGGGCA TTTCTAGATA AGGATTAATA
Pa_64-2   ATAGAGAGGA GATGCCATTT C-CAAGCACA ---GTGTGTG AGG-AGGGCA TTTCTAGATA AGGATTAATA
Pa_64-3   ATAGAGAGGA GATGCCATTT C-CAAGCACA ---GTGTGTG AGG-AGGGCA TTTCTAGATA AGGATTAATA
Pa_64-4   ATAGAGAGGA GATGCCATTT C-CAAGCACA ---GTGTGTG AGG-AGGGCA TTTCTAGATA AGGATTAATA
Pa_79-4   ATAGAGAGGA GATGCAGCGA C--AAACAGT ---CGATGTG CGGAGTTGCA TTTCTAGATA AGGATTAATA
```

841

Bd_06-10	GAAAGAGATG	GGGTGATTTA	TACGGAAATG	GGAGAGGTGA	AAATCCATGA	TCATTTTAGG	ATAAACTGAA
Bd_09-1	GAAAGAGATG	GGGTGATTTA	TACGGAAATG	GGAGAGGTGA	AAATCCATGA	TCATTTTAGG	ATAAACTGAA
Bd_09-2	GAAAGAGATG	GGGTGATTTA	TACGGAAATG	GGAGAGGTGA	AAATCCATGA	TCATTTTAGG	ATAAACTGAA
Bd_09-3	GAAAGAGATG	GGGTGATTTA	TACGGAAATG	GGAGAGGTGA	AAATCCATGA	TCATTTTAGG	ATAAACTGAA
Bd_10-1	GAAAGAGATG	GGGTGATTTA	TACGGAAATG	GGAGAGGTGA	AAATCCATGA	TCATTTTAGG	ATAAACTGAA
Bd_10-2	GAAAGAGATG	GGGTGATTTA	TACGGAAATG	GGAGAGGTGA	AAATCCATGA	TCATTTTAGG	ATAAACTGAA
Bd_10-2b	GAAAGAGATG	GGGTGATTTA	TACGGAAATG	GGAGAGGTGA	AAATCCATGA	TCATTTTAGG	ATAAACTGAA
Bd_11-2	GAAAGAGATG	GGGTGATTTA	TACGGAAATG	GGAGAGGTGA	AAATCCATGA	TCATTTTAGG	ATAAACTGAA
Bd_11-6	GAAAGAGATG	GGGTGATTTA	TACGGAAATG	GGAGAGGTGA	AAATCCATGA	TCATTTTAGG	ATAAACTGAA
Bd_12-2	GAAAGAGATG	GGGTGATTTA	TACGGAAATG	GGAGAGGTGA	AAATCCATGA	TCATTTTAGG	ATAAACTGAA
Bd_13-1	GAAAGAGATG	GGGTGATTTA	TACGGAAATG	GGAGAGGTGA	AAATCCATGA	TCATTTTAGG	ATAAACTGAA
Bd_13-4	GAAAGAGATG	GGGTGATTTA	TACGGAAATG	GGAGAGGTGA	AAATCCATGA	TCATTTTAGG	ATAAACTGAA
Bd_13-5	GAAAGAGATG	GGGTGATTTA	TACGGAAATG	GGAGAGGTGA	AAATCCATGA	TCATTTTAGG	ATAAACTGAA
Bd_14-1	GAAAGAGATG	GGGTGATTTA	TACGGAAATG	GGAGAGGTGA	AAATCCATGA	TCATTTTAGG	ATAAACTGAA
Bd_14-2	GAAAGAGATG	GGGTGATTTA	TACGGAAATG	GGAGAGGTGA	AAATCCATGA	TCATTTTAGG	ATAAACTGAA
Bd_19-5	GAAAGAGATG	GGGTGATTTA	TACGGAAATG	GGAGAGGTGA	AAATCCATGA	TCATTTTAGG	ATAAACTGAA
Bd_19-6	GAAAGAGATG	GGGTGATTTA	TACGGAAATG	GGAGAGGTGA	AAATCCATGA	TCATTTTAGG	ATAAACTGAA
Bd_20-1	GAAAGAGATG	GGGTGATTTA	TACGGAAATG	GGAGAGGTGA	AAATCCATGA	TCATTTTAGG	ATAAACTGAA
Bd_20-2	GAAAGAGATG	GGGTGATTTA	TACGGAAATG	GGAGAGGTGA	AAATCCATGA	TCATTTTAGG	ATAAACTGAA
Bd_22-1	GAAAGAGATG	GGGTGATTTA	TACGGAAATG	GGAGAGGTGA	AAATCCATGA	TCATTTTAGG	ATAAACTGAA
Go_06-1	GAAAGAGATG	GGGTGATTTA	TACGGAAATG	GGAGAGGTGA	AAATCCATGA	TCATTTTAGG	ATAAACTGAA
Go_06-9	GAAAGAGATG	GGGTGATTTA	TACGGAAATG	GGAGAGGTGA	AAATCCATGA	TCATTTTAGG	ATAAACTGAA
Go_07-1	GAAAGAGATG	GGGTGATTTA	TACGGAAATG	GGAGAGGTGA	AAATCCATGA	TCATTTTAGG	ATAAACTGAA
Go_07-5	GAAAGAGATG	GGGTGATTTA	TACGGAAATG	GGAGAGGTGA	AAATCCATGA	TCATTTTAGG	ATAAACTGAA
Go_07-6	GAAAGAGATG	GGGTGATTTA	TACGGAAATG	GGAGAGGTGA	AAATCCATGA	TCATTTTAGG	ATAAACTGAA
Go_07-8	GAAAGAGATG	GGGTGATTTA	TACGGAAATG	GGAGAGGTGA	AAATCCATGA	TCATTTTAGG	ATAAACTGAA
Go_09-2	GAAAGAGATG	GGGTGATTTA	TACGGAAATG	GGAGAGGTGA	AAATCCATGA	TCATTTTAGG	ATAAACTGAA
Go_09-3	GAAAGAGATG	GGGTGATTTA	TACGGAAATG	GGAGAGGTGA	AAATCCATGA	TCATTTTAGG	ATAAACTGAA
Go_09-4	GAAAGAGATG	GGGTGATTTA	TACGGAAATG	GGAGAGGTGA	AAATCCATGA	TCATTTTAGG	ATAAACTGAA
Go_10-1	GAAAGAGATG	GGGTGATTTA	TACGGAAATG	GGAGAGGTGA	AAATCCATGA	TCATTTTAGG	ATAAACTGAA
Go_10-3	GAAAGAGATG	GGGTGATTTA	TACGGAAATG	GGAGAGGTGA	AAATCCATGA	TCATTTTAGG	ATAAACTGAA
Go_11-3	GAAAGAGATG	GGGTGATTTA	TACGGAAATG	GGAGAGGTGA	AAATCCATGA	TCATTTTAGG	ATAAACTGAA
Go_11-5	GAAAGAGATG	GGGTGATTTA	TACGGAAATG	GGAGAGGTGA	AAATCCATGA	TCATTTTAGG	ATAAACTGAA
Go_13-5	GAAAGAGATG	GGGTGATTTA	TACGGAAATG	GGAGAGGTGA	AAATCCATGA	TCATTTTAGG	ATAAACTGAA
Go_14-2	GAAAGAGATG	GGGTGATTTA	TACGGAAATG	GGAGAGGTGA	AAATCCATGA	TCATTTTAGG	ATAAACTGAA
Go_14-3	GAAAGAGATG	GGGTGATTTA	TACGGAAATG	GGAGAGGTGA	AAATCCATGA	TCATTTTAGG	ATAAACTGAA
Go_14-4	GAAAGAGATG	GGGTGATTTA	TACGGAAATG	GGAGAGGTGA	AAATCCATGA	TCATTTTAGG	ATAAACTGAA
Pa_06-2	GAAAGAGACG	GGGTGATTTA	TACGGAAATG	GGAGAGGTGA	AAATCCATGA	TCATTTTAGG	ATAAACTGAA
Pa_14-4	GAAAGAGATG	GGGTGATTTA	TACGGAAATG	GGAGAGGTGA	AAATCCATGA	TCATTTTAGG	ATAAACTGAA
Pa_14-6	GAAAGAGATG	GGGTGATTTA	TACGGAAATG	GGAGAGGTGA	AAATCCATGA	TCATTTTAGG	ATAAACTGAA
Pa_16-1	GAAAGAGATG	GGGTGATTTA	TACGGAAATG	GGAGAGGTGA	AAATCCATGA	TCATTTTAGG	ATAAACTGAA
Pa_19-1	GAAAGAGATG	GGGTGATTTA	TACGGAAATG	GGAGAGGTGA	AAATCCATGA	TCATTTTAGG	ATAAACTGAA
Pa_19-2	GAAAGAGATG	GGGTGATTTA	TACGGAAATG	GGAGAGGTGA	AAATCCATGA	TCATTTTAGG	ATAAACTGAA
Pa_19-3	GAAAGAGATG	GGGTGATTTA	TACGGAAATG	GGAGAGGTGA	AAATCCATGA	TCATTTTAGG	ATAAACTGAA
Pa_21-2	GAAAGAGATG	GGGTGATTTA	TACGGAAATG	GGAGAGGTGA	AAATCCATGA	TCATTTTAGG	ATAAACTGAA
Pa_22-3	GAAAGAGATG	GGGTGATTTA	TACGGAAATG	GGAGAGGTGA	AAATCCATGA	TCATTTTAGG	ATAAACTGAA
Pa_39-1	GAAAGAGATG	GGGTGATTTA	TACGGAAATG	GGAGAGGTGA	AAATCCATGA	TCATTTTAGG	ATAAACTGAA
Pa_39-5	GAAAGAGATG	GGGTGATTTA	TACGGAAATG	GGAGAGGTGA	AAATCCATGA	TCATTTTAGG	ATAAACTGAA
Pa_50-11	GAAAGAGACG	GGGTGATTTA	TACGGAAATG	GGAGAGGTGA	AAATCCATGA	TCATTTTAGG	ATAAACTGAA
Pa_50-12	GAAAGAGACG	GGGTGATTTA	TACGGAAATG	GGAGAGGTGA	AAATCCATGA	TCATTTTAGG	ATAAACTGAA
Pa_50-19	GAAAGAGACG	GGGTGATTTA	TACGGAAATG	GGAGAGGTGA	AAATCCATGA	TCATTTTAGG	ATAAACTGAA
Pa_50-2	GAAAGAGACG	GGGTGATTTA	TACGGAAATG	GGAGAGGTGA	AAATCCATGA	TCATTTTAGG	ATAAACTGAA
Pa_57-2	GAAAGAGACG	GGGTGATTTA	TACGGAAATG	GGAGAGGTGA	AAATCCATGA	TCATTTTAGG	ATAAACTGAA
Pa_57-3	GAAAGAGACG	GGGTGATTTA	TACGGAAATG	GGAGAGGTGA	AAATCCATGA	TCATTTTAGG	ATAAACTGAA
Pa_57-5	GAAAGAGACG	GGGTGATTTA	TACGGAAATG	GGAGAGGTGA	AAATCCATGA	TCATTTTAGG	ATAAACTGAA
Pa_62-19	GAAAGAGATG	GGGTGATTTA	TACGGAAATG	GGAGAGGTGA	AAATCCATGA	TCATTTTAGG	ATAAACTGAA
Pa_62-3	GAAAGAGATG	GGGTGATTTA	TACGGAAATG	GGAGAGGTGA	AAATCCATGA	TCATTTTAGG	ATAAACTGAA
Pa_63-2	GAAAGAGATG	GGGTGATTTA	TACGGAAATG	GGAGAGGTGA	AAATCCATGA	TCATTTTAGG	ATAAACTGAA
Pa_63-3	GAAAGAGATG	GGGTGATTTA	TACGGAAATG	GGAGAGGTGA	AAATCCATGA	TCATTTTAGG	ATAAACTGAA
Pa_63-4	GAAAGAGATG	GGGTGATTTA	TACGGAAATG	GGAGAGGTGA	AAATCCATGA	TCATTTTAGG	ATAAACTGAA
Pa_64-1	GAAAGAGATG	GGGTGATTTA	TACGGAAATG	GGAGAGGTGA	AAATCCATGA	TCATTTTAGG	ATAAACTGAA
Pa_64-2	GAAAGAGATG	GGGTGATTTA	TACGGAAATG	GGAGAGGTGA	AAATCCATGA	TCATTTTAGG	ATAAACTGAA
Pa_64-3	GAAAGAGATG	GGGTGATTTA	TACGGAAATG	GGAGAGGTGA	AAATCCATGA	TCATTTTAGG	ATAAACTGAA
Pa_64-4	GAAAGAGATG	GGGTGATTTA	TACGGAAATG	GGAGAGGTGA	AAATCCATGA	TCATTTTAGG	ATAAACTGAA
Pa_79-4	GAAAGAGATG	GGGTGATTTA	TACGGAAATG	GGAGAGGTGA	AAATCCATGA	TCATTTTAGG	ATAAACTGAA

```
            911
Bd_06-10    GCGAAAGCAT TTCACTTAAA TCTTTTCATT AATCAAGAAC GAAAGTTAGG GGATCGAAGA CGATTAGATA
Bd_09-1     GCGAAAGCAT TTCACTTAAA TCTTTTCATT AATCAAGAAC GAAAGTTAGG GGATCGAAGA CGATTAGATA
Bd_09-2     GCGAAAGCAT TTCACTTAAA TCTTTTCATT AATCAAGAAC GAAAGTTAGG GGATCGAAGA CGATTAGATA
Bd_09-3     GCGAAAGCAT TTCACTTAAA TCTTTTCATT AATCAAGAAC GAAAGTTAGG GGATCGAAGA CGATTAGATA
Bd_10-1     GCGAAAGCAT TTCACTTAAA TCTTTTCATT AATCAAGAAC GAAAGTTAGG GGATCGAAGA CGATTAGATA
Bd_10-2     GCGAAAGCAT TTCACTTAAA TCTTTTCATT AATCAAGAAC GAAAGTTAGG GGATCGAAGA CGATTAGATA
Bd_10-2b    GCGAAAGCAT TTCACTTAAA TCTTTTCATT AATCAAGAAC GAAAGTTAGG GGATCGAAGA CGATTAGATA
Bd_11-2     GCGAAAGCAT TTCACTTAAA TCTTTTCATT AATCAAGAAC GAAAGTTAGG GGATCGAAGA CGATTAGATA
Bd_11-6     GCGAAAGCAT TTCACTTAAA TCTTTTCATT AATCAAGAAC GAAAGTTAGG GGATCGAAGA CGATTAGATA
Bd_12-2     GCGAAAGCAT TTCACTTAAA TCTTTTCATT AATCAAGAAC GAAAGTTAGG GGATCGAAGA CGATTAGATA
Bd_13-1     GCGAAAGCAT TTCACTTAAA TCTTTTCATT AATCAAGAAC GAAAGTTAGG GGATCGAAGA CGATTAGATA
Bd_13-4     GCGAAAGCAT TTCACTTAAA TCTTTTCATT AATCAAGAAC GAAAGTTAGG GGATCGAAGA CGATTAGATA
Bd_13-5     GCGAAAGCAT TTCACTTAAA TCTTTTCATT AATCAAGAAC GAAAGTTAGG GGATCGAAGA CGATTAGATA
Bd_14-1     GCGAAAGCAT TTCACTTAAA TCTTTTCATT AATCAAGAAC GAAAGTTAGG GGATCGAAGA CGATTAGATA
Bd_14-2     GCGAAAGCAT TTCACTTAAA TCTTTTCATT AATCAAGAAC GAAAGTTAGG GGATCGAAGA CGATTAGATA
Bd_19-5     GCGAAAGCAT TTCACTTAAA TCTTTTCATT AATCAAGAAC GAAAGTTAGG GGATCGAAGA CGATTAGATA
Bd_19-6     GCGAAAGCAT TTCACTTAAA TCTTTTCATT AATCAAGAAC GAAAGTTAGG GGATCGAAGA CGATTAGATA
Bd_20-1     GCGAAAGCAT TTCACTTAAA TCTTTTCATT AATCAAGAAC GAAAGTTAGG GGATCGAAGA CGATTAGATA
Bd_20-2     GCGAAAGCAT TTCACTTAAA TCTTTTCATT AATCAAGAAC GAAAGTTAGG GGATCGAAGA CGATTAGATA
Bd_22-1     GCGAAAGCAT TTCACTTAAA TCTTTTCATT AATCAAGAAC GAAAGTTAGG GGATCGAAGA CGATTAGATA
Go_06-1     GCGAAAGCAT TTCACTTAAA TCTGTTCATT AATCAAGAAC GAAAGTTAGG GGATCGAAGA CGATTAGATA
Go_06-9     GCGAAAGCAT TTCACTTAAA TCTGTTCATT AATCAAGAAC GAAAGTTAGG GGATCGAAGA CGATTAGATA
Go_07-1     GCGAAAGCAT TTCACTTAAA TCTGTTCATT AATCAAGAAC GAAAGTTAGG GGATCGAAGA CGATTAGATA
Go_07-5     GCGAAAGCAT TTCACTTAAA TCTGTTCATT AATCAAGAAC GAAAGTTAGG GGATCGAAGA CGATTAGATA
Go_07-6     GCGAAAGCAT TTCACTTAAA TCTGTTCATT AATCAAGAAC GAAAGTTAGG GGATCGAAGA CGATTAGATA
Go_07-8     GCGAAAGCAT TTCACTTAAA TCTGTTCATT AATCAAGAAC GAAAGTTAGG GGATCGAAGA CGATTAGATA
Go_09-2     GCGAAAGCAT TTCACTTAAA TCTTTTCATT AATCAAGAAC GAAAGTTAGG GGATCGAAGA CGATTAGATA
Go_09-3     GCGAAAGCAT TTCACTTAAA TCTTTTCATT AATCAAGAAC GAAAGTTAGG GGATCGAAGA CGATTAGATA
Go_09-4     GCGAAAGCAT TTCACTTAAA TCTTTTCATT AATCAAGAAC GAAAGTTAGG GGATCGAAGA CGATTAGATA
Go_10-1     GCGAAAGCAT TTCACTTAAA TCTGTTCATT AATCAAGAAC GAAAGTTAGG GGATCGAAGA CGATTAGATA
Go_10-3     GCGAAAGCAT TTCACTTAAA TCTGTTCATT AATCAAGAAC GAAAGTTAGG GGATCGAAGA CGATTAGATA
Go_11-3     GCGAAAGCAT TTCACTTAAA TCTGTTCATT AATCAAGAAC GAAAGTTAGG GGATCGAAGA CGATTAGATA
Go_11-5     GCGAAAGCAT TTCACTTAAA TCTGTTCATT AATCAAGAAC GAAAGTTAGG GGATCGAAGA CGATTAGATA
Go_13-5     GCGAAAGCAT TTCACTTAAA TCTGTTCATT AATCAAGAAC GAAAGTTAGG GGATCGAAGA CGATTAGATA
Go_14-2     GCGAAAGCAT TTCACTTAAA TCTGTTCATT AATCAAGAAC GAAAGTTAGG GGATCGAAGA CGATTAGATA
Go_14-3     GCGAAAGCAT TTCACTTAAA TCTGTTCATT AATCAAGAAC GAAAGTTAGG GGATCGAAGA CGATTAGATA
Go_14-4     GCGAAAGCAT TTCACTTAAA TCTGTTCATT AATCAAGAAC GAAAGTTAGG GGATCGAAGA CGATTAGATA
Pa_06-2     GCGAAAGCAT TTCACTTGAA TCTTTTCATT AATCAAGAAC GAAAGTTAGG GGATCGAAGA CGATTAGATA
Pa_14-4     GCGAAAGCAT TTCACTTAAA TCTTTTCATT AATCAAGAAC GAAAGTTAGG GGATCGAAGA CGATTAGATA
Pa_14-6     GCGAAAGCAT TTCACTTAAA TCTTTTCATT AATCAAGAAC GAAAGTTAGG GGATCGAAGA CGATTAGATA
Pa_16-1     GCGAAAGCAT TTCACTTAAA TCTGTTCATT AATCAAGAAC GAAAGTTAGG GGATCGAAGA CGATTAGATA
Pa_19-1     GCGAAAGCAT TTCACTTAAA TCTGTTCATT AATCAAGAAC GAAAGTTAGG GGATCGAAGA CGATTAGATA
Pa_19-2     GCGAAAGCAT TTCACTTAAA TCTGTTCATT AATCAAGAAC GAAAGTTAGG GGATCGAAGA CGATTAGATA
Pa_19-3     GCGAAAGCAT TTCACTTAAA TCTGTTCATT AATCAAGAAC GAAAGTTAGG GGATCGAAGA CGATTAGATA
Pa_21-2     GCGAAAGCAT TTCACTTAAA TCTTTTCATT AATCAAGAAC GAAAGTTAGG GGATCGAAGA CGATTAGATA
Pa_22-3     GCGAAAGCAT TTCACTTAAA TCTTTTCATT AATCAAGAAC GAAAGTTAGG GGATCGAAGA CGATTAGATA
Pa_39-1     GCGAAAGCAT TTCACTTAAA TCTTTTCATT AATCAAGAAC GAAAGTTAGG GGATCGAAGA CGATTAGATA
Pa_39-5     GCGAAAGCAT TTCACTTAAA TCTTTTCATT AATCAAGAAC GAAAGTTAGG GGATCGAAGA CGATTAGATA
Pa_50-11    GCGAAAGCAT TTCACTTGAA TCTTTTCATT AATCAAGAAC GAAAGTTAGG GGATCGAAGA CGATTAGATA
Pa_50-12    GCGAAAGCAT TTCACTTGAA TCTTTTCATT AATCAAGAAC GAAAGTTAGG GGATCGAAGA CGATTAGATA
Pa_50-19    GCGAAAGCAT TTCACTTGAA TCTTTTCATT AATCAAGAAC GAAAGTTAGG GGATCGAAGA CGATTAGATA
Pa_50-2     GCGAAAGCAT TTCACTTGAA TCTTTTCATT AATCAAGAAC GAAAGTTAGG GGATCGAAGA CGATTAGATA
Pa_57-2     GCGAAAGCAT TTCACTTGAA TCTTTTCATT AATCAAGAAC GAAAGTTAGG GGATCGAAGA CGATTAGATA
Pa_57-3     GCGAAAGCAT TTCACTTGAA TCTTTTCATT AATCAAGAAC GAAAGTTAGG GGATCGAAGA CGATTAGATA
Pa_57-5     GCGAAAGCAT TTCACTTGAA TCTTTTCATT AATCAAGAAC GAAAGTTAGG GGATCGAAGA CGATTAGATA
Pa_62-19    GCGAAAGCAT TTCACTTAAA TCTGTTCATT AATCAAGAAC GAAAGTTAGG GGATCGAAGA CGATTAGATA
Pa_62-3     GCGAAAGCAT TTCACTTAAA TCTGTTCATT AATCAAGAAC GAAAGTTAGG GGATCGAAGA CGATTAGATA
Pa_63-2     GCGAAAGCAT TTCACTTAAA TCTTTTCATT AATCAAGAAC GAAAGTTAGG GGATCGAAGA CGATTAGATA
Pa_63-3     GCGAAAGCAT TTCACTTAAA TCTTTTCATT AATCAAGAAC GAAAGTTAGG GGATCGAAGA CGATTAGATA
Pa_63-4     GCGAAAGCAT TTCACTTAAA TCTTTTCATT AATCAAGAAC GAAAGTTAGG GGATCGAAGA CGATTAGATA
Pa_64-1     GCGAAAGCAT TTCACTTAAA TCTTTTCATT AATCAAGAAC GAAAGTTAGG GGATCGAAGA CGATTAGATA
Pa_64-2     GCGAAAGCAT TTCACTTAAA TCTTTTCATT AATCAAGAAC GAAAGTTAGG GGATCGAAGA CGATTAGATA
Pa_64-3     GCGAAAGCAT TTCACTTAAA TCTTTTCATT AATCAAGAAC GAAAGTTAGG GGATCGAAGA CGATTAGATA
Pa_64-4     GCGAAAGCAT TTCACTTAAA TCTTTTCATT AATCAAGAAC GAAAGTTAGG GGATCGAAGA CGATTAGATA
Pa_79-4     GCGAAAGCAT TTCACTTAAA TCTGTTCATT AATCAAGAAC GAAAGTTAGG GGATCGAAGA CGATTAGATA
```

```
            981
Bd_06-10    CCGTCGTAGT CCTAACTATA AACGATGTCA ACGAAGGATT GGATTGAACC AACGCCAGTG ---GTTGCAG
Bd_09-1     CCGTCGTAGT CCTAACTATA AACGATGTCA ACGAAGGATT GGATTGAACC AACGCCAGTG ---GTTGCAG
Bd_09-2     CCGTCGTAGT CCTAACTATA AACGATGTCA ACGAAGGATT GGATTGAACC AACGCCAGTG ---GTTGCAG
Bd_09-3     CCGTCGTAGT CCTAACTATA AACGATGTCA ACGAAGGATT GGATTGAACC AACGCCAGTG ---GTTGCAG
Bd_10-1     CCGTCGTAGT CCTAACTATA AACGATGTCA ACGAAGGATT GGATTGAACC AACGCCAGTG ---GTTGCAG
Bd_10-2     CCGTCGTAGT CCTAACTATA AACGATGTCA ACGAAGGATT GGATTGAACC AACGCCAGTG ---GTTGCAG
Bd_10-2b    CCGTCGTAGT CCTAACTATA AACGATGTCA ACGAAGGATT GGATTGGACC AACGCCAGTG ---GTTGCAG
Bd_11-2     CCGTCGTAGT CCTAACTATA AACGATGTCA ACGAAGGATT GGATTGAACC AACGCCAGTG ---GTTGCAG
Bd_11-6     CCGTCGTAGT CCTAACTATA AACGATGTCA ACGAAGGATT GGATTGAACC AACGCCAGTG ---GTTGCAG
Bd_12-2     CCGTCGTAGT CCTAACTATA AACGATGTCA ACGAAGGATT GGATTGAACC AACGCCAGTG ---GTTGCAG
Bd_13-1     CCGTCGTAGT CCTAACTATA AACGATGTCA ACGAAGGATT GGATTGAACC AACGCCAGTG ---GTTGCAG
Bd_13-4     CCGTCGTAGT CCTAACTATA AACGATGTCA ACGAAGGATT GGATTGAACC AACGCCAGTG ---GTTGCAG
Bd_13-5     CCGTCGTAGT CCTAACTATA AACGATGTCA ACGAAGGATT GGATTGAACC AACGCCAGTG ---GTTGCAG
Bd_14-1     CCGTCGTAGT CCTAACTATA AACGATGTCA ACGAAGGATT GGATTGAACC AACGCCAGTG ---GTTGCAG
Bd_14-2     CCGTCGTAGT CCTAACTATA AACGATGTCA ACGAAGGATT GGATTGAACC AACGCCAGTG ---GTTGCAG
Bd_19-5     CCGTCGTAGT CCTAACTATA AACGATGTCA ACGAAGGATT GGATTGAACC AACGCCAGTG ---GTTGCAG
Bd_19-6     CCGTCGTAGT CCTAACTATA AACGATGTCA ACGAAGGATT GGATTGAACC AACGCCAGTG ---GTTGCAG
Bd_20-1     CCGTCGTAGT CCTAACTATA AACGATGTCA ACGAAGGATT GGATTGAACC AACGCCAGTG ---GTTGCAG
Bd_20-2     CCGTCGTAGT CCTAACTATA AACGATGTCA ACGAAGGATT GGATTGAACC AACGCCAGTG ---GTTGCAG
Bd_22-1     CCGTCGTAGT CCTAACTATA AACGATGTCA ACGAAGGATT GGATTGAACC AACGCCAGTG ---GTTGCAG
Go_06-1     CCGTCGTAGT CCTAACTATA AACGATGTCA ACAAAGGATT GGATTGAACC ATAGCCGACT -----CGGCA
Go_06-9     CCGTCGTAGT CCTAACTATA AACGATGTCA ACAAAGGATT GGATTGAACC ATAGCCGACT -----CGGCA
Go_07-1     CCGTCGTAGT CCTAACTATA AACGATGTCA ACAAAGGATT GGATTGAACC ATAGCCGACC -----CGGCA
Go_07-5     CCGTCGTAGT CCTAACTATA AACGATGTCA ACAAAGGATT GGATTGAACC ATAGCCGACC -----CGGCA
Go_07-6     CCGTCGTAGT CCTAACTATA AACGATGTCA ACAAAGGATT GGATTGAACC ATAGCCAACC -----CGGCA
Go_07-8     CCGTCGTAGT CCTAACTATA AACGATGTCA ACAAAGGATT GGATTGAACC ATAGCCGACT -----CGGCA
Go_09-2     CCGTCGTAGT CCTAACTATA AACGATGTCA ACGAAGGATT GGATTGAACC AACGCCAGTG ---GTTGCAG
Go_09-3     CCGTCGTAGT CCTAACTATA AACGATGTCA ACGAAGGATT GGATTGAACC AACGCCAGTG ---GTTGCAG
Go_09-4     CCGTCGTAGT CCTAACTATA AACGATGTCA ACGAAGGATT GGATTGAACC AACGCCAGTG ---GTTGCAG
Go_10-1     CCGTCGTAGT CCTAACTATA AACGATGTCA ACAAAGGATT GGATTGAACC ATAGCCGACT -----CGGCA
Go_10-3     CCGTCGTAGT CCTAACTATA AACGATGTCA ACAAAGGATT GGATTGAACC ATAGCCGACC -----CGGCA
Go_11-3     CCGTCGTAGT CCTAACTATA AACGATGTCA ACAAAGGATT GGATTGAACC ATAGCCGACC -----CGGCA
Go_11-5     CCGTCGTAGT CCTAACTATA AACGATGTCA ACAAAGGATT GGATTGAACC ATAGCCGACC -----CGGCA
Go_13-5     CCGTCGTAGT CCTAACTATA AACGATGTCA ACAAAGGATT GGATTGAACC ATAGCCGACT -----CGGCA
Go_14-2     CCGTCGTAGT CCTAACTATA AACGATGTCA ACAAAGGATT GGATTGAACC ATAGCCGACT -----CGGCA
Go_14-3     CCGTCGTAGT CCTAACTATA AACGATGTCA ACAAAGGATT GGATTGAACC ATAGCCGACT -----CGGCA
Go_14-4     CCGTCGTAGT CCTAACTATA AACGATGTCA ACAAAGGATT GGATTGAACC ATAGCCGACT -----CGGCA
Pa_06-2     CCGTCGTAGT CCTAACTATA AACGATGTCA ACGAAGGATT GGATTGAATT AAAGCTAGCA CTTGCTTCCG
Pa_14-4     CCGTCGTAGT CCTAACTATA AACGATGTCA ACGAAGGATT GGATTGAACC TTTACCAAAG ---GCTTCTG
Pa_14-6     CCGTCGTAGT CCTAACTATA AACGATGTCA ACGAAGGATT GGATTGAACC TTTACCAAAG ---GCTTCTG
Pa_16-1     CCGTCGTAGT CCTAACTATA AACGATGTCA ACGAAGGATT GGATTGAACC TTTATCGCCA -----CTCCG
Pa_19-1     CCGTCGTAGT CCTAACTATA AACGATGTCA ACGAAGGATT GGATTGAACC TTTATCACAC ---TGTCTCG
Pa_19-2     CCGTCGTAGT CCTAACTATA AACGATGTCA ACGAAGGATT GGATTGAACC TTTATCACAC ---TGTCTCG
Pa_19-3     CCGTCGTAGT CCTAACTATA AACGATGTCA ACGAAGGATT GGATTGAACC TTTATCACAC ---TGTCTCG
Pa_21-2     CCGTCGTAGT CCTAACTATA AACGATGTCA ACGAAGGATT GGATTGAACC ATGGCCGGAG ---GTTGAAG
Pa_22-3     CCGTCGTAGT CCTAACTATA AACGATGTCA ACGAAGGATT GGATTGAACC ATGGCCGGAG ---GTTGAAG
Pa_39-1     CCGTCGTAGT CCTAACTATA AACGATGTCA ACGAAGGATT GGATTGAACA TTTACCGAGG -----CTATG
Pa_39-5     CCGTCGTAGT CCTAACTATA AACGATGTCA ACGAAGGATT GGATTGAACA TTTACCGAGG -----CTATG
Pa_50-11    CCGTCGTAGT CCTAACTATA AACGATGTCA ACGAAGGATT GGATTGAATT AAAGCTAGCA CTTGCTTCCG
Pa_50-12    CCGTCGTAGT CCTAACTATA AACGATGTCA ACGAAGGATT GGATTGAATT AAAGCTAGCA CTTGCTTCCG
Pa_50-19    CCGTCGTAGT CCTAACTATA AACGATGTCA ACGAAGGATT GGATTGAATT AAAGCTAGCA CTTGCTTCCG
Pa_50-2     CCGTCGTAGT CCTAACTATA AACGATGTCA ACGAAGGATT GGATTGAATT AAAGCTAGCA CTTGCTTCCG
Pa_57-2     CCGTCGTAGT CCTAACTATA AACGATGTCA ACGAAGGATT GGATTGAATT AAAGCTAGCA CTTGCTTCCG
Pa_57-3     CCGTCGTAGT CCTAACTATA AACGATGTCA ACGAAGGATT GGATTGAATT AAAGCTAGCA CTTGCTTCCG
Pa_57-5     CCGTCGTAGT CCTAACTATA AACGATGTCA ACGAAGGATT GGATTGAATT AAAGCTAGCA CTTGCTTCCG
Pa_62-19    CCGTCGTAGT CCTAACTATA AACGATGTCA ACGAAGGATT GGATTGAACC TTTACCACAC ---TGTCTCG
Pa_62-3     CCGTCGTAGT CCTAACTATA AACGATGTCA ACGAAGGATT GGATTGAACC TTTACCACAC ---TGTCTCG
Pa_63-2     CCGTCGTAGT CCTAACTATA AACGATGTCA ACGAAGGATT GGATTGAACC ATGGCCGGAG ---GTTGAAG
Pa_63-3     CCGTCGTAGT CCTAACTATA AACGATGTCA ACGAAGGATT GGATTGAACC ATGGCCGGAG ---GTTGAAG
Pa_63-4     CCGTCGTAGT CCTAACTATA AACGATGTCA ACGAAGGATT GGATTGAACC TTTACCCAGG -----CTTGA
Pa_64-1     CCGTCGTAGT CCTAACTATA AACGATGTCA ACGAAGGATT GGATTGAACC TTTACTGCTA ---GGCCTCG
Pa_64-2     CCGTCGTAGT CCTAACTATA AACGATGTCA ACGAAGGATT GGATTGAACC TTTACTGCTA ---GGCCTCG
Pa_64-3     CCGTCGTAGT CCTAACTATA AACGATGTCA ACGAAGGATT GGATTGAACC TTTACTGCTA ---GGCCTCG
Pa_64-4     CCGTCGTAGT CCTAACTATA AACGATGTCA ACGAAGGATT GGATTGAACC TTTACTGCTA ---GGCCTCG
Pa_79-4     CCGTCGTAGT CCTAACTATA AACGATGTCA ACGAAGGATT GGATTGAACC TTTACCACTC -----AACCT
```

```
           1051
Bd_06-10   AACCAACTGG CACTATACTT CAGTTCAGGA CTTG-TGGGG AAACCTATAG TTAATGGACT TCAGGGGGAG
Bd_09-1    AACCAACTGG CACTATACTT CAGTTCAGGA CTTG-TGGGG AAACCTATAG TTAATGGACT TCAGGGGGAG
Bd_09-2    AACCAACTGG CACTATACTT CAGTTCAGGA CTTG-TGGGG AAACCTATAG TTAATGGACT TCAGGGGGAG
Bd_09-3    AACCAACTGG CACTATACTT CAGTTCAGGA CTTG-TGGGG AAACCTATAG TTAATGGACT TCAGGGGGAG
Bd_10-1    AACCAACTGG CACTATACTT CAGTTCAGGA CTTG-TGGGG AAACCTATAG TTAATGGACT TCAGGGGGAG
Bd_10-2    AACCAACTGG CACTATACTT CAGTTCAGGA CTTG-TGGGG AAACCTATAG TTAATGGACT TCAGGGGGAG
Bd_10-2b   AACCAACTGG CACTATACTT CAGTTCAGGA CTTG-TGGGG AAACCTATAG TTAATGGACT TCAGGGGGAG
Bd_11-2    AACCAACTGG CACTATACTT CAGTTCAGGA CTTG-TGGGG AAACCTATAG TTAATGGACT TCAGGGGGAG
Bd_11-6    AACCAACTGG CACTATACTT CAGTTCAGGA CTTG-TGGGG AAACCTATAG TTAATGGACT TCAGGGGGAG
Bd_12-2    AACCAACTGG CACTATACTT CAGTTCAGGA CTTG-TGGGG AAACCTATAG TTAATGGACT TCAGGGGGAG
Bd_13-1    AACCAACTGG CACTATACTT CAGTTCAGGA CTTG-TGGGG AAACCTATAG TTAATGGACT TCAGGGGGAG
Bd_13-4    AACCAACTGG CACTATACTT CAGTTCAGGA CTTG-TGGGG AAACCTATAG TTAATGGACT TCAGGGGGAG
Bd_13-5    AACCAACTGG CACTATACTT CAGTTCAGGA CTTG-TGGGG AAACCTATAG TTAATGGACT TCAGGGGGAG
Bd_14-1    AACCAACTGG CACTATACTT CAGTTCAGGA CTTGTTGGGG AAACCTATAG TTAATGGACT TCAGGGGGAG
Bd_14-2    AACCAACTGG CACTATACTT CAGTTCAGGA CTTG-TGGGG AAACCTATAG TTAATGGACT TCAGGGGGAG
Bd_19-5    AACCAACTGG CACTATACTT CAGTTCAGGA CTTG-TGGGG AAACCTATAG TTAATGGACT TCAGGGGGAG
Bd_19-6    AACCAACTGG CACTATACTT CAGTTCAGGA CTTG-TGGGG AAACCTATAG TTAATGGACT TCAGGGGGAG
Bd_20-1    AACCAACTGG CACTATACTT CAGTTCAGGA CTTG-TGGGG AAACCTATAG TTAATGGACT TCAGGGGGAG
Bd_20-2    AACCAACTGG CACTATACTT CAGTTCAGGA CTTG-TGGGG AAACCTATAG TTAATGGACT TCAGGGGGAG
Bd_22-1    AACCAACTGG CACTATACTT CAGTTCAGGA CTTG-TGGGG AAACCTATAG TTAATGGACT TCAGGGGGAG
Go_06-1    -ACGAGACGG CGCGATACTT CAGTTCAGGA CTTA-TAGGG AAACCTTTAG TTAATGGACT TCAGGGGGAG
Go_06-9    -ACGAGACGG CGCGATACTT CAGTTCAGGA CTTA-TAGGG AAACCTTTAG TTAATGGACT TCAGGGGGAG
Go_07-1    -ACGGGACGG CGCGATACTT CAGTTCAGGA CTTA-TAGGG AAACCTTTAG TTAATGGACT TCAGGGGGAG
Go_07-5    -ACGGGACGG CGCGATACTT CAGTTCAGGA CTTA-TAGGG AAACCTTTAG TTAATGGACT TCAGGGGGAG
Go_07-6    -ACGGGATGG CGCGATACTT CAGTTCAGGA CTTA-TAGGG AAACCTTTAG TTAATGGACT TCAGGGGGAG
Go_07-8    -ACGAGACGG CGCGATACTT CAGTTCAGGA CTTA-TAGGG AAACCTTTAG TTAATGGACT TCAGGGGGAG
Go_09-2    AACCAACTGG CACTATACTT CAGTTCAGGA CTTG-TGGGG AAACCTATAG TTAATGGACT TCAGGGGGAG
Go_09-3    AACCAACTGG CACTATACTT CAGTTCAGGA CTTG-TGGGG AAACCTATAG TTAATGGACT TCAGGGGGAG
Go_09-4    AACCAACTGG CACTATACTT CAGTTCAGGA CTTG-TGGGG AAACCTATAG TTAATGGACT TCAGGGGGAG
Go_10-1    -ACGAGACGG CGCGATACTT CAGTTCAGGA CTTA-TAGGG AAACCTTTAG TTAATGGACT TCAGGGGGAG
Go_10-3    -ACGGGACGG CGCGATACTT CAGTTCAGGA CTTA-TAGGG AAACCTTTAG TTAATGGACT TCAGGGGGAG
Go_11-3    -ACGGGACGG CGCGATACTT CAGTTCAGGA CTTA-TAGGG AAACCTTTAG TTAATGGACT TCAGGGGGAG
Go_11-5    -ACGGGACGG CGCGATACTT CAGTTCAGGA CTTA-TAGGG AAACCTTTAG TTAATGGACT TCAGGGGGAG
Go_13-5    -ACGAGACGG CGCGATACTT CAGTTCAGGA CTTA-TAGGG AAACCTTTAG TTAATGGACT TCAGGGGGAG
Go_14-2    -ACGAGACGG CGCGATACTT CAGTTCAGGA CTTA-TAGGG AAACCTTTAG TTAAT-GACT TCAGGGGGAG
Go_14-3    -ACGAGACGG CGCGATACTT CAGTTCAGGA CTTA-TAGGG AAACCTTTAG TTAATGGACT TCAGGGGGAG
Go_14-4    -ACGAGACGG CGCGATACTT CAGTTCAGGA CTTA-TAGGG AAACCTTTAG TTAATGGACT TCAGGGGGAG
Pa_06-2    CTTGTACTAG CACTATACTT CAGTTCAGGA CTTG-AGGGG AAACCTCTAG TTAATGGACT TCAGGGGGAG
Pa_14-4    ---CCTATGG TAGTATACTT CAGTTCAGGA CTTT-AGGGG AAACCTTTAG TTAATGGACT TCAGGGGGAG
Pa_14-6    ---CCTATGG TAGTATACTT CAGTTCAGGA CTTT-AGGGG AAACCTTTAG TTAATGGACT TCAGGGGGAG
Pa_16-1    ---GTGGTGG TAGTATACTT CAGTTCAGGA CTTA-GAGGG AAACCTATAG TTAATGGACT TCAGGGGGAG
Pa_19-1    -ACAGAGTGG TAGTATACTT CAGTTCAGGA CTTT-AGGGG AAACCTATAG TTAATGGACT TCAGGGGGAG
Pa_19-2    -ACAGAGTGG TAGTATACTT CAGTTCAGGA CTTT-AGGGG AAACCTATAG TTAATGGACT TCAGGGGGAG
Pa_19-3    -ACAGAGTGG TAGTATACTT CAGTTCAGGA CTTT-AGGGG AAACCTATAG TTAATGGACT TCAGGGGGAG
Pa_21-2    AACCAAGCGG CAGTATACTT CAGTTCAGGA CTTG-AGGGG AAACCTTTAG TTAATGGACT TCAGGGGGAG
Pa_22-3    AACCAAGCGG CAGTATACTT CAGTTCAGGA CTTG-AGGGG AAACCTTTAG TTAATGGACT TCAGGGGGAG
Pa_39-1    ---CCTGAGG TAGTATACTT CAGTTCAGGA CTTT-AGGGG AAACCTTTAG TTAATGGACT TCAGGGGGAG
Pa_39-5    ---CCTGAGG TAGTATACTT CAGTTCAGGA CTTT-AGGGG AAACCTTTAG TTAATGGACT TCAGGGGGAG
Pa_50-11   CTTGTACTAG CACTATACTT CAGTTCAGGA CTTG-AGGGG AAACCTCTAG TTAATGGACT TCAGGGGGAG
Pa_50-12   CTTGTACTAG CATTATACTT CAGTTCAGGA CTTG-AGGGG AAACCTTTAG TTAATGGACT TCAGGGGGAG
Pa_50-19   CTTGTACTAG CACTATACTT CAGTTCAGGA CTTG-AGGGG AAACCTCTAG TTAATGGACT TCAGGGGGAG
Pa_50-2    CTTGTACTAG CACTATACTT CAGTTCAGGA CTTG-AGGGG AAACCTCTAG TTAATGGACT TCAGGGGGAG
Pa_57-2    CTTGTACTAG CACTATACTT CAGTTCAGGA CTTG-AGGGG AAACCTCTAG TTAATGGACT TCAGGGGGAG
Pa_57-3    CTTGTACTAG CACTATACTT CAGTTCAGGA CTTG-AGGGG AAACCTCTAG TTAATGGACT TCAGGGGGAG
Pa_57-5    CTTGTACTAG CACTATACTT CAGTTCAGGA CTTG-AGGGG AAACCTCTAG TTAATGGACT TCAGGGGGAG
Pa_62-19   -ACAGAGTGG TAGTATACTT CAGTTCAGGA CTTT-AGGGG AAACCTATAG TTAATGGACT TCAGGGGGAG
Pa_62-3    -ACAGAGTGG TAGTATACTT CAGTTCAGGA CTTT-AGGGG AAACCTATAG TTAATGGACT TCAGGGGGAG
Pa_63-2    AACCAAGCGG CAGTATACTT CAGTTCAGGA CTTG-AGGGG AAACCTTTAG TTAATGGACT TCAGGGGGAG
Pa_63-3    AACCAAGCGG CAGTATACTT CAGTTCAGGA CTTG-AGGGG AAACCTTTAG TTAATGGACT TCAGGGGGAG
Pa_63-4    ---CCTGTGG TAGTATACTT CAGTTCAGGA CTTT-AGGGG AAACCTTTAG TTAATGGACT TCAGGGGGAG
Pa_64-1    -GCCAAGCGG TAGTATACTT CAGTTCAGGA CTTT-AGGGG AAACCTTTAG TTAATGGACT TCAGGGGGAG
Pa_64-2    -GCCAAGCGG TAGTATATTT CAGTTCAGGA CTTT-AGGGG AAACCTTTAG TTAATGGACT TCAGGGGGAG
Pa_64-3    -GCCAAGCGG TAGTATACTT CAGTTCAGGA CTTT-AGGGG AAACCTTTAG TTAATGGACT TCAGGGGGAG
Pa_64-4    -GCCAAGCGG TAGTATACTT CAGTTCAGGA CTTT-AGGGG AAACCTTTAG TTAATGGACT TCAGGGGGAG
Pa_79-4    AGTTGAGAGG TAGTATACTT CAGTTCAGGA CTTG-AGGGG AAACCTTTAG TTAATGGACT TCAGGGGGAG
```

```
            1121
Bd_06-10    TATGCTCGCA AGGGTGAAAC TTAAAGGAAT -------TGAC GGAAGGGCAC ACCAGGAGTG GAGCCTGCGG
Bd_09-1     TATGCTCGCA AGGGTGAAAC TTAAAGGAAT -------TGAC GGAAGGGCAC ACCAGGAGTG GAGCCTGCGG
Bd_09-2     TATGCTCGCA AGGGTGAAAC TTAAAGGAAT -------TGAC GGAAGGGCAC ACCAGGAGTG GAGCCTGCGG
Bd_09-3     TATGCTCGCA AGGGTGAAAC TTAAAGGAAT -------TGAC GGAAGGGCAC ACCAGGAGTG GAGCCTGCGG
Bd_10-1     TATGCTCGCA AGGGTGAAAC TTAAAGGAAT -------TGAC GGAAGGGCAC ACCAGGAGTG GAGCCTGCGG
Bd_10-2     TATGCTCGCA AGGGTGAAAC TTAAAGGAAT -------TGAC GGAAGGGCAC ACCAGGAGTG GAGCCTGCGG
Bd_10-2b    TATGCTCGCA AGGGTGAAAC TTAAAGGAAT -------TGAC GGAAGGGCAC ACCAGGAGTG GAGCCTGCGG
Bd_11-2     TATGCTCGCA AGGGTGAAAC TTAAAGGAAT -------TGAC GGAAGGGCAC ACCAGGAGTG GAGCCTGCGG
Bd_11-6     TATGCTCGCA AGGGTGAAAC TTAAAGGAAT -------TGAC GGAAGGGCAC ACCAGGAGTG GAGCCTGCGG
Bd_12-2     TATGCTCGCA AGGGTGAAAC TTAAAGGAAT -------TGAC GGAAGGGCAC ACCAGGAGTG GAGCCTGCGG
Bd_13-1     TATGCTCGCA AGGGTGAAAC TTAAAGGAAT -------TGAC GGAAGGGCAC ACCAGGAGTG GAGCCTGCGG
Bd_13-4     TATGCTCGCA AGGGTGAAAC TTAAAGGAAT -------TGAC GGAAGGGCAC ACCAGGAGTG GAGCCTGCGG
Bd_13-5     TATGCTCGCA AGGGTGAAAC TTAAAGGAAT -------TGAC GGAAGGGCAC ACCAGGAGTG GAGCCTGCGG
Bd_14-1     TATGCTCGCA AGGGTGAAAC TTAAAGGAAT -------TGAC GGAAGGGCAC ACCAGGAGTG GAGCCTGCGG
Bd_14-2     TATGCTCGCA AGGGTGAAAC TTAAAGGAAT -------TGAC GGAAGGGCAC ACCAGGAGTG GAGCCTGCGG
Bd_19-5     TATGCTCGCA AGGGTGAAAC TTAAAGGAAT -------TGAC GGAAGGGCAC ACCAGGAGTG GAGCCTGCGG
Bd_19-6     TATGCTCGCA AGGGTGAAAC TTAAAGGAAT -------TGAC GGAAGGGCAC ACCAGGAGTG GAGCCTGCGG
Bd_20-1     TATGCTCGCA AGGGTGAAAC TTAAAGGAAT -------TGAC GGAAGGGCAC ACCAGGAGTG GAGCCTGCGG
Bd_20-2     TATGCTCGCA AGGGTGAAAC TTAAAGGAAT -------TGAC GGAAGGGCAC ACCAGGAGTG GAGCCTGCGG
Bd_22-1     TATGCTCGCA AGGGTGAAAC TTAAAGGAAT -------TGAC GGAAGGGCAC ACCAGGAGTG GAGCCTGCGG
Go_06-1     TATAGTCGCA AGGCCGAAAC TTAAAGGAAT -------TGAC GGAAGGGCAC ACCAGGAGTG GAGCCTGCGG
Go_06-9     TATAGTCGCA AGGCCGAAAC TTAAAGGAAT -------TGAC GGAAGGGCAC ACCAGGAGTG GAGCCTGCGG
Go_07-1     TATAGTCGCA AGGCCGAAAC TTAAAGGAAT -------TGAC GGAAGGGCAC ACCAGGAGTG GAGCCTGCGG
Go_07-5     TATAGTCGCA AGGCCGAAAC TTAAAGGAAT -------TGAC GGAAGGGCAC ACCAGGAGTG GAGCCTGCGG
Go_07-6     TATAGTCGCA AGGCCGAAAC TTAAAGGAAT -------TGAC GGAAGGGCAC ACCAGGAGTG GAGCCTGCGG
Go_07-8     TATAGTCGCA AGGCCGAAAC TTAAAGGAAT -------TGAC GGAAGGGCAC ACCAGGAGTG GAGCCTGCGG
Go_09-2     TATGCTCGCA AGGGTGAAAC TTAAAGGAAT -------TGAC GGAAGGGCAC ACCAGGAGTG GAGCCTGCGG
Go_09-3     TATGCTCGCA AGGGTGAAAC TTAAAGGAAT -------TGAC GGAAGGGCAC ACCAGGAGTG GAGCCTGCGG
Go_09-4     TATGCTCGCA AGGGTGAAAC TTAAAGGAAT -------TGAC GGAAGGGCAC ACCAGGAGTG GAGCCTGCGG
Go_10-1     TATAGTCGCA AGGCCGAAAC TTAAAGGAAT -------TGAC GGAAGGGCAC ACCAGGAGTG GAGCCTGCGG
Go_10-3     TATAGTCGCA AGGCCGAAAC TTAAAGGAAT -------TGAC GGAAGGGCAC ACCAGGAGTG GAGCCTGCGG
Go_11-3     TATAGTCGCA AGGCCGAAAC TTAAAGGAAT -------TGAC GGAAGGGCAC ACCAGGAGTG GAGCCTGCGG
Go_11-5     TATAGTCGCA AGGCCGAAAC TTAAAGGAAT -------TGAC GGAAGGGCAC ACCAGGAGTG GAGCCTGCGG
Go_13-5     TATAGTCGCA AGGCCGAAAC TTAAAGGAAT -------TGAC GGAAGGGCAC ACCAGGAGTG GAGCCTGCGG
Go_14-2     TATAGTCGCA AGGCCGAAAC TTAAAGGAAT -------TGAC GGAAGGGCAC ACCAGGAGTG GAGCCTGCGG
Go_14-3     TATAGTCGCA AGGCCGAAAC TTAAAGGAAT -------TGAC GGAAGGGCAC ACCAGGAGTG GAGCCTGCGG
Go_14-4     TATAGTCGCA AGGCCGAAAC TTAAAGGAAT -------TGAC GGAAGGGCAC ACCAGGAGTG GAGCCTGCGG
Pa_06-2     TATGCTCGCA AGGGTGAAAC TTAAAGGAAT -------TGAC GGAAGGGCAC ACCAGGAGTG GAGCCTGCGG
Pa_14-4     TATGCTCGCA AGTGTGAAAC TTAAAGGAAT -------TGAC GGAAGGGCAC ACCAGGAGTG GAGCCTGCGG
Pa_14-6     TATGCTCGCA AGTGTGAAAC TTAAAGGAAT -------TGAC GGAAGGGCAC ACCAGGAGTG GAGCCTGCGG
Pa_16-1     TATGCTCGCA AGGGTGAAAC TTAAAGGAAT -------TGAC GGAAGGGCAC ACCAGGAGTG GAGCCTGCGG
Pa_19-1     TATGCTCGCA AGGGTGAAAC TTAAAGGAAT -------TGAC GGAAGGGCAC ACCAGGAGTG GAGCCTGCGG
Pa_19-2     TATGCTCGCA AGGGTGAAAC TTAAAGGAAT -------TGAC GGAAGGGCAC ACCAGGAGTG GAGCCTGCGG
Pa_19-3     TATGCTCGCA AGGGTGAAAC TTAAAGGAAT -------TGAC GGAAGGGCAC ACCAGGAGTG GAGCCTGCGG
Pa_21-2     TATGCTCGCA AGGGTGAAAC TTAAAGGAAT -------TGAC GGAAGGGCAC ACCAGGAGTG GAGCCTGCGG
Pa_22-3     TATGCTCGCA AGGGTGAAAC TTAAAGGAAT -------TGAC GGAAGGGCAC ACCAGGAGTG GAGCCTGCGG
Pa_39-1     TATGCTCGCA AGTGTGAAAC TTAAAGGAAT -------TGAC GGAAGGGCAC ACCAGGAGTG GAGCCTGCGG
Pa_39-5     TATGCTCGCA AGTGTGAAAC TTAAAGGAAT -------TGAC GGAAGGGCAC ACCAGGAGTG GAGCCTGCGG
Pa_50-11    TATGCTCGCA AGGGTGAAAC TTAAAGGAAT -------TGAC GGAAGGGCAC ACCAGGAGTG GAGCCTGCGG
Pa_50-12    TATGCTCGCA AGGGTGAAAC TTAAAGGAAT -------TGAC GGAAGGGCAC ACCAGGAGTG GAGCCTGCGG
Pa_50-19    TATGCTCGCA AGGGTGAAAC TTAAAGGAAT -------TGAC GGAAGGGCAC ACCAGGAGTG GAGCCTGCGG
Pa_50-2     TATGCTCGCA AGGGTGAAAC TTAAAGGAAT -------TGAC GGAAGGGCAC ACCAGGAGTG GAGCCTGCGG
Pa_57-2     TATGCTCGCA AGGGTGAAAC TTAAAGGAAT -------TGAC GGAAGGGCAC ACCAGGAGTG GAGCCTGCGG
Pa_57-3     TATGCTCGCA AGGGTGAAAC TTAAAGGAAT GACGGATGAC GGAAGGGCAC ACCAGGAGTG GAGCCTGCGG
Pa_57-5     TATGCTCGCA AGGGTGAAAC TTAAAGGAAT -------TGAC GGAAGGGCAC ACCAGGAGTG GAGCCTGCGG
Pa_62-19    TATGCTCGCA AGGGTGAAAC TTAAAGGAAT -------TGAC GGAAGGGCAC ACCAGGAGTG GAGCCTGCGG
Pa_62-3     TATGCTCGCA AGGGTGAAAC TTAAAGGAAT -------TGAC GGAAGGGCAC ACCAGGAGTG GAGCCTGCGG
Pa_63-2     TATGCTCGCA AGGGTGAAAC TTAAAGGAAT -------TGAC GGAAGGGCAC ACCAGGAGTG GAGCCTGCGG
Pa_63-3     TATGCTCGCA AGGGTGAAAC TTAAAGGAAT -------TGAC GGAAGGGCAC ACCAGGAGTG GAGCCTGCGG
Pa_63-4     TATGCTCGCA AGTGTGAAAC TTAAAGGAAT -------TGAC GGAAGGGCAC ACCAGGAGTG GAGCCTGCGG
Pa_64-1     TATGCTCGCA AGGGTGAAAC TTAAAGGAAT -------TGAC GGAAGGGCAC ACCAGGAGTG GAGCCTGCGG
Pa_64-2     TATGCTCGCA AGGGTGAAAC TTAAAGGAAT -------TGAC GGAAGGGCAC ACCAGGAGTG GAGCCTGCGG
Pa_64-3     TATGCTCGCA AGGGTGAAAC TTAAAGGAAT -------TGAC GGAAGGGCAC ACCAGGAGTG GAGCCTGCGG
Pa_64-4     TATGCTCGCA AGGGTGAAAC TTAAAGGAAT -------TGAC GGAAGGGCAC ACCAGGAGTG GAGCCTGCGG
Pa_79-4     TATGCTCGCA AGGGTGAAAC TTAAAGGAAT -------TGAC GGAAGGGCAC ACCAGGAGTG GAGCCTGCGG
```

```
            1191
Bd_06-10    CTTAATTTGA CTCAACACGG GAAAACTTAC CAAGACCAGA CATTAAGAGG AGTGACAGAG TAAGGGAACT
Bd_09-1     CTTAATTTGA CTCAACACGG GAAAACTTAC CAAGACCAGA CATTAAGAGG AGTGACAGAG TAAGGGAACT
Bd_09-2     CTTAATTTGA CTCAACACGG GAAAACTTAC CAAGACCAGA CATTAAGAGG AGTGACAGAG TAAGGGAACT
Bd_09-3     CTTAATTTGA CTCAACACGG GAAAACTTAC CAAGACCAGA CATTAAGAGG AGTGACAGAG TAAGGGAACT
Bd_10-1     CTTAATTTGA CTCAACACGG GAAAACTTAC CAAGACCAGA CATTAAGAGG AGTGACAGAG TAAGGGAACT
Bd_10-2     CTTAATTTGA CTCAACACGG GAAAACTTAC CAAGACCAGA CATTAAGAGG AGTGACAGAG TAAGGGAACT
Bd_10-2b    CTTAATTTGA CTCAACACGG GAAAACTTAC CAAGACCAGA CATTAAGAGG AGTGACAGAG TAAGGGAACT
Bd_11-2     CTTAATTTGA CTCAACACGG GAAAACTTAC CAAGACCAGA CATTAAGAGG AGTGACAGAG TAAGGGAACT
Bd_11-6     CTTAATTTGA CTCAACACGG GAAAACTTAC CAAGACCAGA CATTAAGAGG AGTGACAGAG TAAGGGAACT
Bd_12-2     CTTAATTTGA CTCAACACGG GAAAACTTAC CAAGACCAGA CATTAAGAGG AGTGACAGAG TAAGGGAACT
Bd_13-1     CTTAATTTGA CTCAACACGG GAAAACTTAC CAAGACCAGA CATTAAGAGG AGTGACAGAG TAAGGGAACT
Bd_13-4     CTTAATTTGA CTCAACACGG GAAAACTTAC CAAGACCAGA CATTAAGAGG AGTGACAGAG TAAGGGAACT
Bd_13-5     CTTAATTTGA CTCAACACGG GAAAACTTAC CAAGACCAGA CATTAAGAGG AGTGACAGAG TAAGGGAACT
Bd_14-1     CTTAATTTGA CTCAACACGG GAAAACTTAC CAAGACCAGA CATTAAGAGG AGTGACAGAG TAAGGGAACT
Bd_14-2     CTTAATTTGA CTCAACACGG GAAAACTTAC CAAGACCAGA CATTAAGAGG AGTGACAGAG TAAGGGAACT
Bd_19-5     CTTAATTTGA CTCAACACGG GAAAACTTAC CAAGACCAGA CATTAAGAGG AGTGACAGAG TAAGGGAACT
Bd_19-6     CTTAATTTGA CTCAACACGG GAAAACTTAC CAAGACCAGA CATTAAGAGG AGTGACAGAG TAAGGGAACT
Bd_20-1     CTTAATTTGA CTCAACACGG GAAAACTTAC CAAGACCAGA CATTAAGAGG AGTGACAGAG TAAGGGAACT
Bd_20-2     CTTAA-TTGA CTCAACACGG GAAAACTTAC CAAGACCAGA CATTAAGAGG AGTGACAGAG TAAGGGAACT
Bd_22-1     CTTAATTTGA CTCAACACGG GAAAACTTAC CAAGACCAGA CATTAAGAGG AGTGACAGAG TAAGGGAACT
Go_06-1     CTTAATTTGA CTCAACACGG GAAAACTTAC CAAGACCAGA CATTAAGAGG AGTGACAGAG TAAGGGAACT
Go_06-9     CTTAATTTGA CTCAACACGG GAAAACTTAC CAAGACCAGA CATTAAGAGG AGTGACAGAG TAAGGGAACT
Go_07-1     CTTAATTTGA CTCAACACGG GAAAACTTAC CAAGACCAGA CATTAAGAGG AGTGACAGAG TAAGGGAACT
Go_07-5     CTTAATTTGA CTCAACACGG GAAAACTTAC CAAGACCAGA CATTAAGAGG AGTGACAGAG TAAGGGAACT
Go_07-6     CTTAATTTGA CTCAACACGG GAAAACTTAC CAAGACCAGA CATTAAGAGG AGTGACAGAG TAAGGGAACT
Go_07-8     CTTAATTTGA CTCAACACGG GAAAACTTAC CAAGACCAGA CATTAAGAGG AGTGACAGAG TAAGGGAACT
Go_09-2     CTTAATTTGA CTCAACACGG GAAAACTTAC CAAGACCAGA CATTAAGAGG AGTGACAGAG TAAGGGAACT
Go_09-3     CTTAATTTGA CTCAACACGG GAAAACTTAC CAAGACCAGA CATTAAGAGG AGTGACAGAG TAAGGGAACT
Go_09-4     CTTAATTTGA CTCAACACGG GAAAACTTAC CAAGACCAGA CATTAAGAGG AGTGACAGAG TAAGGGAACT
Go_10-1     CTTAATTTGA CTCAACACGG GAAAACTTAC CAAGACCAGA CATTAAGAGG AGTGACAGAG TAAGGGAACT
Go_10-3     CTTAATTTGA CTCAACACGG GAAAACTTAC CAAGACCAGA CATTAAGAGG AGTGACAGAG TAAGGGAACT
Go_11-3     CTTAATTTGA CTCAACACGG GAAAACTTAC CAAGACCAGA CATTAAGAGG AGTGACAGAG TAAGGGAACT
Go_11-5     CTTAATTTGA CTCAACACGG GAAAACTTAC CAAGACCAGA CATTAAGAGG AGTGACAGAG TAAGGGAACT
Go_13-5     CTTAATTTGA CTCAACACGG GAAAACTTAC CAAGACCAGA CATTAAGAGG AGTGACAGAG TAAGGGAACT
Go_14-2     CTTAATTTGA CTCAACACGG GAAAACTTAC CAAGACCAGA CATTAAGAGG AGTGACAGAG TAAGGGAACT
Go_14-3     CTTAATTTGA CTCAACACGG GAAAACTTAC CAAGACCAGA CATTAAGAGG AGTGACAGAG TAAGGGAACT
Go_14-4     CTTAATTTGA CTCAACACGG GAAAACTTAC CAAGACCAGA CATTAAGAGG AGTGACAGAG TAAGGGAACT
Pa_06-2     CTTAATTTGA CTCAACACGG GAAAACTTAC CAAGACCAGA CATTAAGAGG AGTGACAGAG TAAGGGAACT
Pa_14-4     CTTAATTTGA CTCAACACGG GAAAACTTAC CAAGACCATA CATTAAGAGG AGTGACAGAG TAAGGGAACT
Pa_14-6     CTTAATTTGA CTCAACACGG GAAAACTTAC CAAGACCATA CATTAAGAGG AGTGACAGAG TAAGGGAACT
Pa_16-1     CTTAATTTGA CTCAACACGG GAAAACTTAC CAAGACCAGA CATTAAGAGG AGTGACAGAG TAAGGGAACT
Pa_19-1     CTTAATTTGA CTCAACACGG GAAAACTTAC CAAGACCAGA CATTAAGAGG AGTGACAGAG TAAGGGAACT
Pa_19-2     CTTAATTTGA CTCAACACGG GAAAACTTAC CAAGACCAGA CATTAAGAGG AGTGACAGAG TAAGGGAACT
Pa_19-3     CTTAATTTGA CTCAACACGG GAAAACTTAC CAAGACCAGA CATTAAGAGG AGTGACAGAG TAAGGGAACT
Pa_21-2     CTTAATTTGA CTCAACACGG GAAAACTTAC CAAGACCAGA CATTAAGAGG AGTGACAGAG TAAGGGAACT
Pa_22-3     CTTAATTTGA CTCAACACGG GAAAACTTAC CAAGACCAGA CATTAAGAGG AGTGACAGAG TAAGGGAACT
Pa_39-1     CTTAATTTGA CTCAACACGG GAAAACTTAC CAAGACCAGA CATTAAGAGG AGTGACAGAG TAAGGGAACT
Pa_39-5     CTTAATTTGA CTCAACACGG GAAAACTTAC CAAGACCAGA CATTAAGAGG AGTGACAGAG TAAGGGAACT
Pa_50-11    CTTAATTTGA CTCAACACGG GAAAACTTAC CAAGACCAGA CATTAAGAGG AGTGACAGAG TAAGGGAACT
Pa_50-12    CTTAATTTGA CTCAACACGG GAAAACTTAC CAAGACCAGA CATTAAGAGG AGTGACAGAG TAAGGGAACT
Pa_50-19    CTTAATTTGA CTCAACACGG GAAAACTTAC CAAGACCAGA CATTAAGAGG AGTGACAGAG TAAGGGAACT
Pa_50-2     CTTAATTTGA CTCAACACGG GAAAACTTAC CAAGACCAGA CATTAAGAGG AGTGACAGAG TAAGGGAACT
Pa_57-2     CTTAATTTGA CTCAACACGG GAAAACTTAC CAAGACCAGA CATTAAGAGG AGTGACAGAG TAAGGGAACT
Pa_57-3     CTTAATTTGA CTCAACACGG GAAAACTTAC CAAGACCAGA CATTAAGAGG AGTGACAGAG TAAGGGAACT
Pa_57-5     CTTAATTTGA CTCAACACGG GAAAACTTAC CAAGACCAGA CATTAAGAGG AGTGACAGAG TAAGGGAACT
Pa_62-19    CTTAATTTGA CTCAACACGG GAAAACTTAC CAAGACCAGA CATTAAGAGG AGTGACAGAG TAAGGGAACT
Pa_62-3     CTTAATTTGA CTCAACACGG GAAAACTTAC CAAGACCAGA CATTAAGAGG AGTGACAGAG TAAGGGAACT
Pa_63-2     CTTAATTTGA CTCAACACGG GAAAACTTAC CAAGACCAGA CATTAAGAGG AGTGACAGAG TAAGGGAACT
Pa_63-3     CTTAATTTGA CTCAACACGG GAAAACTTAC CAAGACCAGA CATTAAGAGG AGTGACAGAG TAAGGGAACT
Pa_63-4     CTTAATTTGA CTCAACACGG GAAAACTTAC CAAGACCAGA CATTAAGAGG AGTGACAGAG TAAGGGAACT
Pa_64-1     CTTAATTTGA CTCAACACGG GAAAACTTAC CAAGACCAGA CATTAAGAGG AGTGACAGAG TAAGGGAACT
Pa_64-2     CTTAATTTGA CTCAACACGG GAAAACTTAC CAAGACCAGA CATTAAGAGG AGTGACAGAG TAAGGGAACT
Pa_64-3     CTTAATTTGA CTCAACACGG GAAAACTTAC CAAGACCAGA CATTAAGAGG AGTGACAGAG TAAGGGAACT
Pa_64-4     CTTAATTTGA CTCAACACGG GAAAACTTAC CAAGACCAGA CATTAAGAGG AGTGACAGAG TAAGGGAACT
Pa_79-4     CTTAATTTGA CTCAACACGG GAAAACTTAC CAAGACCAGA CATTAAGAGG AGTGACAGAG TAAGGGAACT
```

```
          1261
Bd_06-10  TTCATGATTT GATGGAGAGT GGTGCATGGC CGTTCTTAGT TGGTGGAGTG ATTTGTCAGG TTTATTCCGA
Bd_09-1   TTCATGATTT GATGGAGAGT GGTGCATGGC CGTTCTTAGT TGGTGGAGTG ATTTGTCAGG TTTATTCCGA
Bd_09-2   TTCATGATTT GATGGAGAGT GGTGCATGGC CGTTCTTAGT TGGTGGAGTG ATTTGTCAGG TTTATTCCGA
Bd_09-3   TTCATGATTT GATGGAGAGT GGTGCATGGC CGTTCTTAGT TGGTGGAGTG ATTTGTCAGG TTTATTCCGA
Bd_10-1   TTCATGATTT GATGGAGAGT GGTGCATGGC CGTTCTTAGT TGGTGGAGTG ATTTGTCAGG TTTATTCCGA
Bd_10-2   TTCATGATTT GATGGAGAGT GGTGCATGGC CGTTCTTAGT TGGTGGAGTG ATTTGTCAGG TTTATTCCGA
Bd_10-2b  TTCATGATTT GATGGAGAGT GGTGCATGGC CGTTCTTAGT TGGTGGAGTG ATTTGTCAGG TTTATTCCGA
Bd_11-2   TTCATGATTT GATGGAGAGT GGTGCATGGC CGTTCTTAGT TGGTGGAGTG ATTTGTCAGG TTTATTCCGA
Bd_11-6   TTCATGATTT GATGGAGAGT GGTGCATGGC CGTTCTTAGT TGGTGGAGTG ATTTGTCAGG TTTATTCCGA
Bd_12-2   TTCATGATTT GATGGAGAGT GGTGCATGGC CGTTCTTAGT TGGTGGAGTG ATTTGTCAGG TTTATTCCGA
Bd_13-1   TTCATGATTT GATGGAGAGT GGTGCATGGC CGTTCTTAGT TGGTGGAGTG ATTTGTCAGG TTTATTCCGA
Bd_13-4   TTCATGATTT GATGGAGAGT GGTGCATGGC CGTTCTTAGT TGGTGGAGTG ATTTGTCAGG TTTATTCCGA
Bd_13-5   TTCATGATTT GATGGAGAGT GGTGCATGGC CGTTCTTAGT TGGTGGAGTG ATTTGTCAGG TTTATTCCGA
Bd_14-1   TTCATGATTT GATGGAGAGT GGTGCATGGC CGTTCTTAGT TGGTGGAGTG ATTTGTCAGG TTTATTCCGA
Bd_14-2   TTCATGATTT GATGGAGAGT GGTGCATGGC CGTTCTTAGT TGGTGGAGTG ATTTGTCAGG TTTATTCCGA
Bd_19-5   TTCATGATTT GATGGAGAGT GGTGCATGGC CGTTCTTAGT TGGTGGAGTG ATTTGTCAGG TTTATTCCGA
Bd_19-6   TTCATGATTT GATGGAGAGT GGTGCATGGC CGTTCTTAGT TGGTGGAGTG ATTTGTCAGG TTTATTCCGA
Bd_20-1   TTCATGATTT GATGGAGAGT GGTGCATGGC CGTTCTTAGT TGGTGGAGTG ATTTGTCAGG TTTATTCCGA
Bd_20-2   TTCATGATTT GATGGAGAGT GGTGCATGGC CGTTCTTAGT TGGTGGAGTG ATTTGTCAGG TTTATTCCGA
Bd_22-1   TTCATGATTT GATGGAGAGT GGTGCATGGC CGTTCTTAGT TGGTGGAGTG ATTTGTCAGG TTTATTCCGA
Go_06-1   TTCATGATTT GATGGAGAGT GGTGCATGGC CGTTCTTAGT TGGTGGAGTG ATTTGTCAGG TTTATTCCGA
Go_06-9   TTCATGATTT GATGGAGAGT GGTGCATGGC CGTTCTTAGT TGGTGGAGTG ATTTGTCAGG TTTATTCCGA
Go_07-1   TTCATGATTT GATGGAGAGT GGTGCATGGC CGTTCTTAGT TGGTGGAGTG ATTTGTCAGG TTTATTCCGA
Go_07-5   TTCATGATTT GATGGAGAGT GGTGCATGGC CGTTCTTAGT TGGTGGAGTG ATTTGTCAGG TTTATTCCGA
Go_07-6   TTCATGATTT GATGGAGAGT GGTGCATGGC CGTTCTTAGT TGGTGGAGTG ATTTGTCAGG TTTATTCCGA
Go_07-8   TTCATGATTT GATGGAGAGT GGTGCATGGC CGTTCTTAGT TGGTGGAGTG ATTTGTCAGG TTTATTCCGA
Go_09-2   TTCATGATTT GATGGAGAGT GGTGCATGGC CGTTCTTAGT TGGTGGAGTG ATTTGTCAGG TTTATTCCGA
Go_09-3   TTCATGATTT GATGGAGAGT GGTGCATGGC CGTTCTTAGT TGGTGGAGTG ATTTGTCAGG TTTATTCCGA
Go_09-4   TTCATGATTT GATGGAGAGT GGTGCATGGC CGTTCTTAGT TGGTGGAGTG ATTTGTCAGG TTTATTCCGA
Go_10-1   TTCATGATTT GATGGAGAGT GGTGCATGGC CGTTCTTAGT TGGTGGAGTG ATTTGTCAGG TTTATTCCGA
Go_10-3   TTCATGATTT GATGGAGAGT GGTGCATGGC CGTTCTTAGT TGGTGGAGTG ATTTGTCAGG TTTATTCCGA
Go_11-3   TTCATGATTT GATGGAGAGT GGTGCATGGC CGTTCTTAGT TGGTGGAGTG ATTTGTCAGG TTTATTCCGA
Go_11-5   TTCATGATTT GATGGAGAGT GGTGCATGGC CGTTCTTAGT TGGTGGAGTG ATTTGTCAGG TTTATTCCGA
Go_13-5   TTCATGATTT GATGGAGAGT GGTGCATGGC CGTTCTTAGT TGGTGGAGTG ATTTGTCAGG TTTATTCCGA
Go_14-2   TTCATGATTT GATGGAGAGT GGTGCATGGC CGTTCTTAGT TGGTGGAGTG ATTTGTCAGG TTTATTCCGA
Go_14-3   TTCATGATTT GATGGAGAGT GGTGCATGGC CGTTCTTAGT TGGTGGAGTG ATTTGTCAGG TTTATTCCGA
Go_14-4   TTCATGATTT GATGGAGAGT GGTGCATGGC CGTTCTTAGT TGGTGGAGTG ATTTGTCAGG TTTATTCCGA
Pa_06-2   TTCATGATTT GATGGAGAGT GGTGCATGGC CGTTCTTAGT TGGTGGAGTG ATTTGTCAGG TTTATTCCGA
Pa_14-4   TTCATGATTT GATGGAGAGT GGTGCATGGC CGTTCTTAGT TGGTGGAGTG ATTTGTCAGG TTTATTCCGA
Pa_14-6   TTCATGATTT GATGGAGAGT GGTGCATGGC CGTTCTTAGT TGGTGGAGTG ATTTGTCAGG TTTATTCCGA
Pa_16-1   TTCATGATTT GATGGAGAGT GGTGCATGGC CGTTCTTAGT TGGTGGAGTG ATTTGTCAGG TTAATTCCGA
Pa_19-1   TTCATGATTT GATGGAGAGT GGTGCATGGC CGTTCTTAGT TGGTGGAGTG ATTTGTCAGG TTTATTCCGA
Pa_19-2   TTCATGATTT GATGGAGAGT GGTGCATGGC CGTTCTTAGT TGGTGGAGTG ATTTGTCAGG TTTATTCCGA
Pa_19-3   TTCATGATTT GATGGAGAGT GGTGCATGGC CGTTCTTAGT TGGTGGAGTG ATTTGTCAGG TTTATTCCGA
Pa_21-2   TTCATGATTT GATGGAGAGT GGTGCATGGC CGTTCTTAGT TGGTGGAGTG ATTTGTCAGG TTTATTCCGA
Pa_22-3   TTCATGATTT GATGGAGAGT GGTGCATGGC CGTTCTTAGT TGGTGGAGTG ATTTGTCAGG TTTATTCCGA
Pa_39-1   TTCATGATTT GATGGAGAGT GGTGCATGGC CGTTCTTAGT TGGTGGAGTG ATTTGTCAGG TTTATTCCGA
Pa_39-5   TTCATGATTT GATGGAGAGT GGTGCATGGC CGTTCTTAGT TGGTGGAGTG ATTTGTCAGG TTTATTCCGA
Pa_50-11  TTCATGATTT GATGGAGAGT GGTGCATGGC CGTTCTTAGT TGGTGGAGTG ATTTGTCAGG TTTATTCCGA
Pa_50-12  TTCATGATTT GATGGAGAGT GGTGCATGGC CGTTCTTAGT TGGTGGAGTG ATTTGTCAGG TTTATTCCGA
Pa_50-19  TTCATGATTT GATGGAGAGT GGTGCATGGC CGTTCTTAGT TGGTGGAGTG ATTTGTCAGG TTTATTCCGA
Pa_50-2   TTCATGATTT GATGGAGAGT GGTGCATGGC CGTTCTTAGT TGGTGGAGTG ATTTGTCAGG TTTATTCCGA
Pa_57-2   TTCATGATTT GATGGAGAGT GGTGCATGGC CGTTCTTAGT TGGTGGAGTG ATTTGTCAGG TTTATTCCGA
Pa_57-3   TTCATGATTT GATGGAGAGT GGTGCATGGC CGTTCTTAGT TGGTGGAGTG ATTTGTCAGG TTTATTCCGA
Pa_57-5   TTCATGATTT GATGGAGAGT GGTGCATGGC CGTTCTTAGT TGGTGGAGTG ATTTGTCAGG TTTATTCCGA
Pa_62-19  TTCATGATTT GATGGAGAGT GGTGCATGGC CGTTCTTAGT TGGTGGAGTG ATTTGTCAGG TTTATTCCGA
Pa_62-3   TTCATGATTT GATGGAGAGT GGTGCATGGC CGTTCTTAGT TGGTGGAGTG ATTTGTCAGG TTTATTCCGA
Pa_63-2   TTCATGATTT GATGGAGAGT GGTGCATGGC CGTTCTTAGT TGGTGGAGTG ATTTGTCAGG TTTATTCCGA
Pa_63-3   TTCATGATTT GATGGAGAGT GGTGCATGGC CGTTCTTAGT TGGTGGAGTG ATTTGTCAGG TTTATTCCGA
Pa_63-4   TTCATGATTT GATGGAGAGT GGTGCATGGC CGTTCTTAGT TGGTGGAGTG ATTTGTCAGG TTTATTCCGA
Pa_64-1   TTCATGATTT GATGGAGAGT GGTGCATGGC CGTTCTTAGT TGGTGGAGTG ATTTGTCAGG TTTATTCCGA
Pa_64-2   TTCATGATTT GATGGAGAGT GGTGCATGGC CGTTCTTAGT TGGTGGAGTG ATTTGTCAGG TTTATTCCGA
Pa_64-3   TTCATGATTT GATGGAGAGT GGTGCATGGC CGTTCTTAGT TGGTGGAGTG ATTTGTCAGG TTTATTCCGA
Pa_64-4   TTCATGATTT GATGGAGAGT GGTGCATGGC CGTTCTTAGT TGGTGGAGTG ATTTGTCAGG TTTATTCCGA
Pa_79-4   TTCATGATTT GATGGAGAGT GGTGCATGGC CGTTCTTAGT TGGTGGAGTG ATTTGTCAGG TTTATTCCGA
```

```
             1331
Bd_06-10     TAACGAACGA GACTTCAATC TTTAATTAGA TCACCGTGCT --CCAGTGCG GCGAGCCAGT GATGGCCCTC
Bd_09-1      TAACGAACGA GACTTCAATC TTTAATTAGA TCACCGTGCT --CCAGTGCG GCGAGCCAGT GATGGCCCTC
Bd_09-2      TAACGAACGA GACTTCAATC TTTAATTAGA TCACCGTGCT --CCAGTGCG GCGAGCCAGT GATGGCCCTC
Bd_09-3      TAACGAACGA GACTTCAATC TTTAATTAGA TCACCGTGCT --CCAGTGCG GCGAGCCAGC GATGGCCCTC
Bd_10-1      TAACGAACGA GACTTCAATC TTTAATTAGA TCACCGTGCT --CCAGTGCG GCGAGCCAGT GATGGCCCTC
Bd_10-2      TAACGAACGA GACTTCAATC TTTAATTAGA TCACCGTGCT --CCAGTGCG GCGAGCCAGT GATGGCCCTC
Bd_10-2b     TAACGAACGA GACTTCAATC TTTAATTAGA TCACCGTGCT --CCAGTGCG GCGAGCCAGT GATGGCCCTC
Bd_11-2      TAACGAACGA GACTTCAATC TTTAATTAGA TCACCGTGCT --CCAGTGCG GCGAGCCAGT GATGGCCCTC
Bd_11-6      TAACGAACGA GACTTCAATC TTTAATTAGA TCACCGTGCT --CCAGTGCG GCGAGCCAGT GATGGCCCTC
Bd_12-2      TAACGAACGA GACTTCAATC TTTAATTAGA TCACCGTGCT --CCAGTGCG GCGAGCCAGT GATGGCCCTC
Bd_13-1      TAACGAACGA GACTTCAATC TTTAATTAGA TCACCGTGCT --CCAGTGCG GCGAGCCAGT GATGGCCCTC
Bd_13-4      TAACGAACGA GACTTCAATC TTTAATTAGA TCACCGTGCT --CCAGTGCG GCGAGCCAGT GATGGCCCTC
Bd_13-5      TAACGAACGA GACTTCAATC TTTAATTAGA TCACCGTGCT --CCAGTGCG GCGAGCCAGT GATGGCCCTC
Bd_14-1      TAACGAACGA GACTTCAATC TTTAATTAGA TCACCGTGCT --CCAGTGCG GCGAGCCAGT GATGGCCCTC
Bd_14-2      TAACGAACGA GACTTCAATC TTTAATTAGA TCACCGTGCT --CCAGTGCG GCGAGCCAGT GATGGCCCTC
Bd_19-5      TAACGAACGA GACTTCAATC TTTAATTAGA TCACCGTGCT --CCAGTGCG GCGAGCCAGT GATGGCCCTC
Bd_19-6      TAACGAACGA GACTTCAATC TTTAATTAGA TCACCGTGCT --CCAGTGCG GCGAGCCAGT GATGGCCCTC
Bd_20-1      TAACGAACGA GACTTCAATC TTTAATTAGA TCACCGTGCT --CCAGTGCG GCGAGCCAGT GATGGCCCTC
Bd_20-2      TAACGAACGA GACTTCAATC TTTAATTAGA TCACCGTGCT --CCAGTGCG GCGAGCCAGC GATGGCCCTC
Bd_22-1      TAACGAACGA GACTTCAATC TTTAATTAGA TCACCGTGCT --CCAGTGCG GCGAGCCAGC GATGGCCCTC
Go_06-1      TAACGAACGA GACTTCAATC TTTAATTAGA TGGCGCAGCC --CTGGTTGC GCTAGCCAGC AATGGCCCTC
Go_06-9      TAACGAACGA GACTTCAATC TTTAATTAGA TGGCGCAGCC --CTGGTTGC GCTAGCCAGC AATGGCCCTC
Go_07-1      TAACGAACGA GACTTCAATC TTTAATTAGA TGGCGCAGCC --CTGGTTGC GCTAGCCAGC AATGGCCCTC
Go_07-5      TAACGAACGA GACTTCAATC TTTAATTAGA TGGCGCAGCC --CTGGTTGC GCTAGCCAGC AATGGCCCTC
Go_07-6      TAACGAACGA GACTTCAATC TTTAATTAGA TGGCGCAGCT --TCTGTTGC GCTAGCCAGC AATGGCCCTC
Go_07-8      TAACGAACGA GACTTCAATC TTTAATTAGA TGGCGCAGCC --TCGGTTGC GCTAGCCAGC AATGGCCCTC
Go_09-2      TAACGAACGA GACTTCAATC TTTAATTAGA TCACCGTGCT --CTAGTGCG GCGAGCCAGT GATGGCCCTC
Go_09-3      TAACGAACGA GACTTCAATC TTTAATTAGA TCACCGTGCT --CCAGTGCG GCGAGCCAGT GATGGCCCTC
Go_09-4      TAACGAACGA GACTTCAATC TTTAATTAGA TCACCGTGCT --CCAGTGCG GCGAGCCAGT GATGGCCCTC
Go_10-1      TAACGAACGA GACTTCAATC TTTAATTAGA TGGCGCAGCC --CTGGTTGC GCTAGCCAGC AATGGCCCTC
Go_10-3      TAACGAACGA GACTTCAATC TTTAATTAGA TGGCGCAGCC --CTGGTTGC GCTAGCCAGC AATGGCCCTC
Go_11-3      TAACGAACGA GACTTCAATC TTTAATTAGA TGGCGCAGCT --TCTGTTGC GCTAGCCAGC AATGGCCCTC
Go_11-5      TAACGAACGA GACTTCAATC TTTAATTAGA TGGCGCAGCC --CTGGTTGC GCTAGCCAGC AATGGCCCTC
Go_13-5      TAACGAACGA GACTTCAATC TTTAATTAGA TGGCGCAGCC --TCGGTTGC GCTAGCCAGC AATGGCCCTC
Go_14-2      TAACGAACGA GACTTCAATC TTTAATTAGA TGGCGCAGCC --TCGGTTGC GCTAGCCAGC AATGGCCCTC
Go_14-3      TAACGAACGA GACTTCAATC TTTAATTAGA TGGCGCAGCC --CTGGTTGC GCTAGCCAGC AATGGCCCTC
Go_14-4      TAACGAACGA GACTTCAATC TTTAATTAGA TGGCGCAGCC --TCGGTTGC GCTAGCCAGC AATGGCCCTC
Pa_06-2      TAACGAACGA GACTTTAATC TTTAATTAGA TCACATAGCT C-CATGTTAT GTGAGCCAGC AATGGCCCTC
Pa_14-4      TAACGAACGA GACTTCAATC TTTAATTAGA TCACGCAGCA --CTTGTTGC GGGAGCCAGC AATGGCCCTC
Pa_14-6      TAACGAACGA GACTTCAATC TTTAATTAGA TCACGCAGCA --CTTGTTGC GGGAGCCAGC AATGGCCCTC
Pa_16-1      TAACGAACGA GACTTCAATC TTTAATTAGA TTGCGCAGCA C-TTTGTTGC GGAAGCCAGC AATGGCCCTC
Pa_19-1      TAACGAACGA GACTTCAATC TTTAATTAGA TTACGCAGCA C-CTGGTTGC GCAAGTCAGC AATGACCCTC
Pa_19-2      TAACGAACGA GACTTCAATC TTTAATTAGA TTACGCAGCA C-CTGGTTGC GCAAGTCAGC AATGACCCTC
Pa_19-3      TAACGAACGA GACTTCAATC TTTAATTAGA TTACGCAGCA C-CTGGTTGC GCAAGTCAGC AATGACCCTC
Pa_21-2      TAACGAACGA GACTTCAATC TTTAATTAGA TTGCACAGCA TTTATGTTGT GTGAGCCAGC AATGGCCCTC
Pa_22-3      TAACGAACGA GACTTCAATC TTTAATTAGA TTGCACAGCA TTTATGTTGT GTGAGCCAGC AATGGCCCTC
Pa_39-1      TAACGAACGA GACTTCAATC TTTAATTAGA TTGCACAGCA CTTCTGTTGT GCGAGCCAGC AATGGCCCTC
Pa_39-5      TAACGAACGA GACTTCAATC TTTAATTAGA TTGCACAGCA CTTCTGTTGT GCGAGCCAGC AATGGCCCTC
Pa_50-11     TAACGAACGA GACTTTAATC TTTAATTAGA TCACATAGCT C-TATGTTAT GTGAGCCAGC AATGGCCCTC
Pa_50-12     TAACGAACGA GACTTTAATC TTTAATTAGA TCACATAGCT C-TATGTTAT GTGAGCCAGC AATGGCCCTC
Pa_50-19     TAACGAACGA GACTTTAATC TTTAATTAGA TCACATAGCT C-TATGTTAT GTGAGCCAGC AATGGCCCTC
Pa_50-2      TAACGAACGA GACTTTAATC TTTAATTAGA TCACATAGCT C-TATGTTAT GTGAGCCAGC AATGGCCCTC
Pa_57-2      TAACGAACGA GACTTTAATC TTTAATTAGA TCACATAGCT C-CATGTTAT GTGAGCCAGC AATGGCCCTC
Pa_57-3      TAACGAACGA GACTTTAATC TTTAATTAGA TCACATAGCT C-CATGTTAT GTGAGCCAGC AATGGCCCTC
Pa_57-5      TAACGAACGA GACTTTAATC TTTAATTAGA TCACATAGCT C-CATGTTAT GTGAGCCAGC AATGGCCCTC
Pa_62-19     TAACGAACGA GACTTCAATC TTTAATTAGA TTACGCAGCG --CTGGTTGC GCAAGTCAGC AATGACCCTC
Pa_62-3      TAACGAACGA GACTTCAATC TTTAATTAGA TTACGCAGCG --CTGGTTGC GCAAGTCAGC AATGACCCTC
Pa_63-2      TAACGAACGA GACTTCAATC TTTAATTAGA TCACCGTGCT --CCAGTACG GTGAGCCAGC AATGGCCCTC
Pa_63-3      TAACGAACGA GACTTCAATC TTTAATTAGA TCACCGTGCT --CCAGTACG GTGAGCCAGC AATGGCCCTC
Pa_63-4      TAACGAACGA GACTTCAATC TTTAATTAGA TGACACAGCA --ACTGTTGT GTGAGCCAGC AATGGCCCTC
Pa_64-1      TAACGAACGA GACTTCAATC TTTAATTAGA TGGCGCAGCT --CTAGTTGC GTGAGCCAGC AATGGCCCTC
Pa_64-2      TAACGAACGA GACTTCAATC TTTAATTAGA TGGCGCAGCT --CTAGTTGC GTGAGCCAGC AATGGCCCTC
Pa_64-3      TAACGAACGA GACTTCAATC TTTAATTAGA TGGCGCAGCT --CTAGTTGC GTGAGCCAGC AATGGCCCTC
Pa_64-4      TAACGAACGA GACTTCAATC TTTAATTAGA TGGCGCAGCT --CTAGTTGC GTGAGCCAGC AATGGCCCTC
Pa_79-4      TAACGAACGA GACTTCAATC TTTAATTAGA TCACGCCGCA --CCTGTGGC GCGAGTCAGA AATGACCCTC
```

```
         1401
Bd_06-10  -TCACGAGGT CTTCTTAAAG AGACTCTTGG CAATTTTCCG AG-TGCGCAC TGACCAA-CA CGGCGGTGAG
Bd_09-1   -TCACGAGGT CTTCTTAAAG AGACTCTTGG CAATTTTCCG AG-TGCGCAT CGACCAA-CA CGGCGGTGAG
Bd_09-2   -TCACGAGGT CTTCTTAAAG AGACTCTTGG CAATTTTCCG AG-TGCGCAT CGACCAA-CA CGGCGGTGAG
Bd_09-3   -TCACGAGGT CTTCTTAAAG AGACTCTTGG CAATTTTCCG AG-TGCGCAC TGACCAA-CA CGGCGGTGAG
Bd_10-1   -TCACGAGGT CTTCTTAAAG AGACTCTTGG CAATTTTCCG AG-TGCGCAT CGACCAA-CA CGGCGGTGAG
Bd_10-2   -TCACGAGGT CTTCTTAAAG AGACTCTTGG CAATTTTCCG AG-TGCGCAC TGACCAA-CA CGGCGGTGAG
Bd_10-2b  -TCACGAGGT CTTCTTAAAG AGACTCTTGG CAATTTTCCG AG-TGCGCAC TGACCAA-CA CGGCGGTGAG
Bd_11-2   -TCACGAGGT CTTCTTAAAG AGACTCTTGG CAATTTTCCG AG-TGCGCAC TGACCAA-CA CGGCGGTGAG
Bd_11-6   -TCACGAGGT CTTCTTAAAG AGACTCTTGG CAATTTTCCG AG-TGCGCAT CGACCAA-CA CGGCGGTGAG
Bd_12-2   -TCACGAGGT CTTCTTAAAG AGACTCTTGG CAATTTTCCG AG-TGCGCAC TGACCAA-CA CGGCGGTGAG
Bd_13-1   -TCACGAGGT CTTCTTAAAG AGACTCTTGG CAATTTTCCG AG-TGCGCAC TGACCAA-CA CGGCGGTGAG
Bd_13-4   -TCACGAGGT CTTCTTAAAG AGACTCTTGG CAATTTTCCG AG-TGCGCAT TGACCAA-CA CGGCGGTGAG
Bd_13-5   -TCACGAGGT CTTCTTAAAG AGACTCTTGG CAATTTTCCG AG-TGCGCAC TGACCAA-CA CGGCGGTGAG
Bd_14-1   -TCACGAGGT CTTCTTAAAG AGACTCTTGG CAATTTTCCG AG-TGCGCAC TGACCAA-CA CGGCGGTGAG
Bd_14-2   -TCACGAGGT CTTCTTAAAG AGACTCTTGG CAATTTTCCG AG-TGCGCAC TGACCAA-CA CGGCGGTGAG
Bd_19-5   -TCACGAGGT CTTCTTAAAG AGACTCTTGG CAATTTTCCG AG-TGCGCAT CGACCAA-CA CGGCGGTGAG
Bd_19-6   -TCACGAGGT CTTCTTAAAG AGACTCTTGG CAATTTTCCG AG-TGCGCAT CGACCAA-CA CGGCGGTGAG
Bd_20-1   -TCACGAGGT CTTCTTAAAG AGACTCTTGG CAATTTTCCG AG-TGCGCAC TGACCAA-CA CGGCGGTGAG
Bd_20-2   -TCACGAGGT CTTCTTAAAG AGACTCTTGG CAATTTTCCG AG-TGCGCAC TGACCAA-CA CGGCGGTGAG
Bd_22-1   -TCACGAGGT CTTCTTAAAG AGACTCTTGG CAATTTTCCG AG-TGCGCAC TGACCAA-CA CGGCGGTGAG
Go_06-1   -AAACGAGGT CATCTTAAAG AGACTCTTGC CAATTTTCCG GACTGCACAC CTACCTA-CG GGAGGCTGCG
Go_06-9   -AATCGAGGT CATCTTAAAG AGACTCTTGC CAATTTTCCG GACTGCACAC CTACCTA-CG GGAGGCTGCG
Go_07-1   -AAACGAGGT CATCTTAAAG AGACTCTTGC CAATTTTCCG GACTGCACAC CTACCTA-CG GGAGGCTGCG
Go_07-5   -AATCGAGGT CATCTTAAAG AGACTCTTGC CAATTTTCCG GACTGCACAC CTACCTA-CG GGAGGCTGCG
Go_07-6   -AAACGAGGT CATCTTAAAG AGACTCTTGC CAATTTTCCG GACTGCACAC CTACCTA-CG GGAGGCTGCG
Go_07-8   -ATTCGAGGT CATCTTAAAG AGACTCTTGC CAATTTTCCG GACTGCACAC CTACCTA-CG GGAGGCTGCG
Go_09-2   -TCACGAGGT CTTCTTAAAG AGACTCTTGG CAATTTTCCG AG-TGCGCAC TGACCAA-CA CGGCGGTGAG
Go_09-3   -TCACGAGGT CTTCTTAAAG AGACTCTTGG CAATTTTCCG AG-TGCGCAC TGACCAA-CA CGGCGGTGAG
Go_09-4   -TCACGAGGT CTTCTTAAAG AGACTCTTGG CAATTTTCCG AG-TGCGCAC TGACCAA-CA CGGCGGTGAG
Go_10-1   -AATCGAGGT CATCTTAAAG AGACTCTTGC CAATTTTCCG GACTGCACAC CTACCTA-CG GGAGGCTGCG
Go_10-3   -AATCGAGGT CATCTTAAAG AGACTCTTGC CAATTTTCCG GACTGCACAC CTACCTA-CG GGAGGCTGCG
Go_11-3   -AATCGAGGT CATCTTAAAG AGACTCTTGC CAATTTTCCG GACTGCACAC CTACCTA-CG GGAGGCTGCG
Go_11-5   -AATCGAGGT CATCTTAAAG AGACTCTTGC CAATTTTCCG GACTGCACAC CTACCTA-CG GGAGGCTGCG
Go_13-5   -ATTCGAGGT CATCTTAAAG AGACTCTTGC CAATTTTCCG GACTGCACAC CTACCTA-CG GGAGGCTGCG
Go_14-2   -ATTCGAGGT CATCTTAAAG AGACTCTTGC CAATTTTCCG GACTGCACAC CTACCTA-CG GGAGGCTGCG
Go_14-3   -AATCGAGGT CATCTTAAAG AGACTCTTGC CAATTTTCCG GACTGCACAC CTACCTA-CG GGAGGCTGCG
Go_14-4   -ATTCGAGGT CATCTTAAAG AGACTCTTGC CAATTTTCCG GACTGCACAC CTACCTA-CG GGAGGCTGCG
Pa_06-2   TCATCGAGGT CTTCTTAAAG AGACTCTTGA CAATTTTCTC ACATGCTCAT TGACCAA-AC AGGTAGTGAG
Pa_14-4   --AATGAGGT CTTCTTAAAG AGACTCTTGG CAATTTTCCG GACGGGAGGC CTACCAATCG GGAGGACCTT
Pa_14-6   --AATGAGGT CTTCTTAAAG AGACTCTTGG CAATTTTCCG GACGGGAGGC CTACCAATCG GGAGGACCTT
Pa_16-1   -AAACGAGGT CTTCTTAAAG AGACTCTTGG CAATTTTC-A GTCAGCGGGA CTACCCCTCG GGGAGGCTCG
Pa_19-1   -AAACGAGGT CTTCTTAAAG AGACTCTTGG CAATTTTCTG GACTGCGGAC CTACCCACCG GGAGGATTCG
Pa_19-2   -AAACGAGGT CTTCTTAAAG AGACTCTTGG CAATTTTCTG GACTGCGGAC CTACCCACCG GGAGGATTCG
Pa_19-3   -AAACGAGGT CTTCTTAAAG AGACTCTTGG CAATTTTCTG GACTGCGGAC CTACCCACCG GGAGGATTCG
Pa_21-2   --AATGAGGT CTTCTTAAAG AGACTCTTGG CAATTTTCTG GACTTCAGAC CTACCCAATG GGAGGCTCTG
Pa_22-3   --AATGAGGT CTTCTTAAAG AGACTCTTGG CAATTTTCTG GACTTCAGAC CTACCCAATG GGAGGCTCTG
Pa_39-1   --AATGAGGT CTTCTTAAAG AGACTCTTGG CAATTTTCTG GTCTTCAGAC CTACCCAATG GGAGGCTCTG
Pa_39-5   --AATGAGGT CTTCTTAAAG AGACTCTTGG CAATTTTCTG GTCTTCAGAC CTACCCAATG GGAGGCTCTG
Pa_50-11  TCATCGAGGT CTTCTTAAAG AGACTCTTGA CAATTTTCTC ACATGCTCAT TGACCAA-AC AGGTAGTGAG
Pa_50-12  TCATCGAGGT CTTCTTAAAG AGACTCTTGA CAATTTTCTC ACATGCTCAT TGACCAA-AC AGGTAGTGAG
Pa_50-19  TCATCGAGGT CTTCTTAAAG AGACTCTTGA CAATTTTCTC ACATGCTCAT TGACCAA-AC AGGTAGTGAG
Pa_50-2   TCATCGAGGT CTTCTTAAAG AGACTCTTGA CAATTTTCTC ACATGCTCAT TGACCAA-AC AGGTAGTGAG
Pa_57-2   TCATCGAGGT CTTCTTAAAG AGACTCTTGA CAATTTTCTC ACATGCTCAT TGACCAA-AC AGGTAGTGAG
Pa_57-3   TCATCGAGGT CTTCTTAAAG AGACTCTTGA CAATTTTCTC ACATGCTCAT TGACCAA-AC AGGTAGTGAG
Pa_57-5   TCATCGAGGT CTTCTTAAAG AGACTCTTGA CAATTTTCTC ACATGCTCAT TGACCAA-AC AGGTAGTGAG
Pa_62-19  -AAACGAGGT CTTCTTAAAG AGACTCTTGG CAATTTTCTG GACTGCGGAC CTACCCACCG GGAGGATTCG
Pa_62-3   -AAACGAGGT CTTCTTAAAG AGACTCTTGG CAATTTTCTG GACTGCGGAC CTACCCACCG GGAGGATTCG
Pa_63-2   -ACGCGAGGT CATCTTAAAG AGACTCTTGG CAATTTTCTG ATCTGCGCAC TGACCAA-CT TGGCGGTGAG
Pa_63-3   -ACGCGAGGT CATCTTAAAG AGACTCTTGG CAATTTTCTG ATCTGCGCAC TGACCAA-CT TGGCGGTGAG
Pa_63-4   --AATGAGGT CTTCTTAAAG AGACTCTTGG CAATTTTCCG GATTTCAGAC CTACCTTTCG GGAGGATCTG
Pa_64-1   -AAACGAGGT CTTCTTAAAG AGACTCTTGG CAATTTTCCG GCCAGCGGAC CTACCCAACG GGAGGATCCG
Pa_64-2   -AAACGAGGT CTTCTTAAAG AGACTCTTGG CAATTTTCCG GCCAGCGGAC CTACCCAACG GGAGGATCCG
Pa_64-3   -AAACGAGGT CTTCTTAAAG AGACTCTTGG CAATTTTCCG GCCAGCGGAC CTACCCAACG GGAGGATCCG
Pa_64-4   -AAACGAGGT CTTCTTAAAG AGACTCTTGG CAATTTTCCG GCCAGCGGAC CTACCCAACG GGAGGATCCG
Pa_79-4   --AGTGAGGT CTTCTTAAAG GGACTCTTGG CAATTTTCCG GACTGCGGAC CTACCCA-CG GGAGGATCGG
```

```
             1471
Bd_06-10     TAGTCGGAAC AAGCTAAGGG AAGAGAAGCA ATAACAGGTC TGTGATGCCC TTAGACATCT TGGACTGCAC
Bd_09-1      TAGTCGGAAC AAGCTAAGGG AAGAGAAGCA ATAACAGGTC TGTGATGCCC TTAGACATCT TGGGCTGCAC
Bd_09-2      TAGTCGGAAC AAGCTAAGGG AAGAGAAGCA ATAACAGGTC TGTGATGCCC TTAGACATCT TGGGCTGCAC
Bd_09-3      TAGTCGGAAC AAGCTAAGGG AAGAGAAGCA ATAACAGGTC TGTGATGCCC TTAGACATCT TGGGCTGCAC
Bd_10-1      TAGTCGGAAC AAGCTAAGGG AAGAGAAGCA ATAACAGGTC TGTGATGCCC TTAGACATCT TGGGCTGCAC
Bd_10-2      TAGTCGGAAC AAGCTAAGGG AAGAGAAGCA ATAACAGGTC TGTGATGCCC TTAGACATCT TGGGCTGCAC
Bd_10-2b     TAGTCGGAAC AAGCTAAGGG AAGAGAAGCA ATAACAGGTC TGTGATGCCC TTAGACATCT TGGGCTGCAC
Bd_11-2      TAGTCGGAAC AAGCTAAGGG AAGAGAAGCA ATAACAGGTC TGTGATGCCC TTAGACATCT TGGGCTGCAC
Bd_11-6      TAGTCGGAAC AAGCTAAGGG AAGAGAAGCA ATAACAGGTC TGTGATGCCC TTAGACATCT TGGGCTGCAC
Bd_12-2      TAGTCGGAAC AAGCTAAGGG AAGAGAAGCA ATAACAGGTC TGTGATGCCC TTAGACATCT TGGGCTGCAC
Bd_13-1      TAGTCGGAAC AAGCTAAGGG AAGAGAAGCA ATAACAGGTC TGTGATGCCC TTAGACATCT TGGGCTGCAC
Bd_13-4      TAGTCGGAAC AAGCTAAGGG AAGAGAAGCA ATAACAGGTC TGTGATGCCC TTAGACATCT TGGGCTGCAC
Bd_13-5      TAGTCGGAAC AAGCTAAGGG AAGAGAAGCA ATAACAGGTC TGTGATGCCC TTAGACATCT TGGGCTGCAC
Bd_14-1      TAGTCGGAAC AAGCTAAGGG AAGAGAAGCA ATAACAGGTC TGTGATGCCC TTAGACATCT TGGGCTGCAC
Bd_14-2      TAGTCGGAAC AAGCTAAGGG AAGAGAAGCA ATAACAGGTC TGTGATGCCC TTAGACATCT TGGGCTGCAC
Bd_19-5      TAGTCGGAAC AAGCTAAGGG AAGAGAAGCA ATAACAGGTC TGTGATGCCC TTAGACATCT TGGGCTGCAC
Bd_19-6      TAGTCGGAAC AAGCTAAGGG AAGAGAAGCA ATAACAGGTC TGTGATGCCC TTAGACATCT TGGGCTGCAC
Bd_20-1      TAGTCGGAAC AAGCTAAGGG AAGAGAAGCA ATAACAGGTC TGTGATGCCC TTAGACATCT TGGGCTGCAC
Bd_20-2      TAGTCGGAAC AAGCTAAGGG AAGAGAAGCA ATAACAGGTC TGTGATGCCC TTAGACATCT TGGGCTGCAC
Bd_22-1      TAGTCGGAAC AAGCTAAGGG AAGAGAAGCA ATAACAGGTC TGTGATGCCC TTAGACATCT TGGGCTGCAC
Go_06-1      CAGGCGGAAG AAGGTAAGGG AAGAGAAGCA ATAACAGGTC TGTGATGCCC TTAGACATCT TGGGCTGCAC
Go_06-9      CAGGCGGAAG AAGGTAAGGG AAGAGAAGCA ATAACAGGTC TGTGATGCCC TTAGACATCT TGGGCTGCAC
Go_07-1      CAGGCGGAAG AAGGTAAGGG AAGAGAAGCA ATAACAGGTC TGTGATGCCC TTAGACATCT TGGGCTGCAC
Go_07-5      CAGGCGGAAG AAGGTAAGGG AAGAGAAGCA ATAACAGGTC TGTGATGCCC TTAGACATCT TGGGCTGCAC
Go_07-6      CAGGCGGAAG AAGGTAAGGG AAGAGAAGCA ATAACAGGTC TGTGATGCCC TTAGACATCT TGGGCTGCAC
Go_07-8      CAGGCGGAAG AAGGTAAGGG AAGAGAAGCA ATAACAGGTC TGTGATGCCC TTAGACATCT TGGGCTGCAC
Go_09-2      TAGTCGGAAC AAGCTAAGGG AAGAGAAGCA ATAACAGGTC TGTGATGCCC TTAGACATCT TGGGCTGCAC
Go_09-3      TAGTCGGAAC AAGCTAAGGG AAGAGAAGCA ATAACAGGTC TGTGATGCCC TTAGACATCT TGGGCTGCAC
Go_09-4      TAGTCGGAAC AAGCTAAGGG AAGAGAAGCA ATAACAGGTC AGTGATGCCC TTAGACATCT TGGGCTGCAC
Go_10-1      CAGGCGGAAG AAGGTAAGGG AAGAGAAGCA ATAACAGGTC TGTGATGCCC TTAGACATCT TGGGCTGCAC
Go_10-3      CAGGCGGAAG AAGGTAAGGG AAGAGAAGCA ATAACAGGTC TGTGATGCCC TTAGACATCT TGGGCTGCAC
Go_11-3      CAGGCGGAAG AAGGTAAGGG AAGAGAAGCA ATAACAGGTC TGTGATGCCC TTAGACATCT TGGGCTGCAC
Go_11-5      CAGGCGGAAG AAGGTAAGGG AAGAGAAGCA ATAACAGGTC TGTGATGCCC TTAGACATCT TGGGCTGCAC
Go_13-5      CAGGCGGAAG AAGGTAAGGG AAGAGAAGCA ATAACAGGTC TGTGATGCCC TTAGACATCT TGGGCTGCAC
Go_14-2      CAGGCGGAAG AAGGTAAGGG AAGAGAAGCA ATAACAGGTC TGTGATGCCC TTAGACATCT TGGGCTGCAC
Go_14-3      CAGGCGGAAG AAGGTAAGGG AAGAGAAGCA ATAACAGGTC TGTGATGCCC TTAGACATCT TGGGCTGCAC
Go_14-4      CAGGCGGAAG AAGGTAAGGG AAGAGAAGCA ATAACAGGTC TGTGATGCCC TTAGACATCT TGGGCTGCAC
Pa_06-2      TATTGAGAAC AAGTTAAGGG AAGAGAAGCA ATAACAGGTC TGTGATGCCC TTAGACATCT TGGGCTGCAC
Pa_14-4      CTGCTGGAAG AAGCTAAGGG AAGAGAAGCA ATAACAGGTC TGTGATGCCC TTAGACATCT TGGGCTGCAC
Pa_14-6      CTGCTGGAAG AAGCTAAGGG AAGAGAAGCA ATAACAGGTC TGTGATGCCC TTAGACATCT TGGGCTGCAC
Pa_16-1      TAGGCCGAAG AAGCTAAGGG AAGAGAAGCA ATAACAGGTC TGTGATGCCC TTAGACATCT TGGGCTGCAC
Pa_19-1      CAGGCGGAAG AAGCTAAGGG AAGAGAAGCA ATAACAGGTC TGTGATGCCC TTAGACATCT TGGGCTGCAC
Pa_19-2      CAGGCGGAAG AAGCTAAGGG AAGAGAAGCA ATAACAGGTC TGTGATGCCC TTAGACATCT TGGGCTGCAC
Pa_19-3      CAGGCGGAAG AAGCTAAGGG AAGAGAAGCA ATAACAGGTC TGTGATGCCC TTAGACATCT TGGGCTGCAC
Pa_21-2      GAGCTAGAAG AAGCTAAGGG AAGAGAAGCA ATAACAGGTC TGTGATGCCC TTAGACATCT TGGGCTGCAC
Pa_22-3      GAGCTAGAAG AAGCTAAGGG AAGAGAAGCA ATAACAGGTC TGTGATGCCC TTAGACATCT TGGGCTGCAC
Pa_39-1      GAGCTAGAAG AAGCTAAGGG AAGAGAAGCA ATAACAGGTC TGTGATGCCC TTAGACATCT TGGGCTGCAC
Pa_39-5      GAGCTAGAAG AAGCTAAGGG AAGAGAAGCA ATAACAGGTC TGTGATGCCC TTAGACATCT TGGGCTGCAC
Pa_50-11     TATGGAGAAC AAGTTAAGGG AAGAGAAGCA ATAACAGGTC TGTGATGCCC TTAGACATCT TGGGCTGCAC
Pa_50-12     TATTGGGAAC AAGTTAAGGG AAGAGAAGCA ATAACAGGTC TGTGATGCCC TTAGACATCT TGGGCTGCAC
Pa_50-19     TATGGAGAAC AAGTTAAGGG AAGAGAAGCA ATAACAGGTC TGTGATGCCC TTAGACATCT TGGGCTGCAC
Pa_50-2      TATGGAGAAC AAGTTAAGGG AAGAGAAGCA ATAACAGGTC TGTGATGCCC TTAGACATCT TGGGCTGCAC
Pa_57-2      TATGGAGAAC AAGTTAAGGG AAGAGAAGCA ATAACAGGTC TGTGATGCCC TTAGACATCT TGGGCTGCAC
Pa_57-3      TATGGAGAAC AAGTTAAGGG AAGAGAAGCA ATAACAGGTC TGTGATGCCC TTAGACATCT TGGGCTGCAC
Pa_57-5      TATGGAGAAC AAGTTAAGGG AAGAGAAGCA ATAACAGGTC TGTGATGCCC TTAGACATCT TGGGCTGCAC
Pa_62-19     CAGGCGGAAG AAGCTAAGGG AAGAGAAGCA ATAACAGGTC TGTGATGCCC TTAGACATCT TGGGCTGCAC
Pa_62-3      CAGGCGGAAG AAGCTAAGGG AAGAGAAGCA ATAACAGGTC TGTGATGCCC TTAGACATCT TGGGCTGCAC
Pa_63-2      TAGTCAGAAG AAGCTAAGGG AAGAGAAGCA ATAACAGGTC TGTGATGCCC TTAGACATCT TGGGCTGCAC
Pa_63-3      TAGTCAGAAG AAGCTAAGGG AAGAGAAGCA ATAACAGGTC TGTGATGCCC TTAGACATCT TGGGCTGCAC
Pa_63-4      GAGCTGGAAG AAGCTAAGGG AAGAGAAGCA ATAACAGGTC TGTGATGCCC TTAGACATCT TGGGCTGCAC
Pa_64-1      CCGACGGAAG AAGCTAAGGG AAGAGAAGCA ATAACAGGTC TGTGATGCCC TTAGACATCT TGGGCTGCAC
Pa_64-2      CCGACGGAAG AAGCTAAGGG AAGAGAAGCA ATAACAGGTC TGTGATGCCC TTAGACATCT TGGGCTGCAC
Pa_64-3      CCGACGGAAG AAGCTAAGGG AAGAGAAGCA ATAACAGGTC TGTGATGCCC TTAGACATCT TGGGCTGCAC
Pa_64-4      CCGACGGAAG AAGCTAAGGG AAGAGAAGCA ATAACAGGTC TGTGATGCCC TTAGACATCT TGGGCTGCAC
Pa_79-4      CAGCCAGAAG AAGCTAAGGG AAGAGAAGCA ATAACAGGTC TGTGATGCCC TTAGACATCT TGGGCTGCAC
```

```
                 1541
Bd_06-10    GCGTGCTACA ATATCTATTT TCAAGAGCAC TTTAAACTGC TCATAGGAGA CCATTGCCTC CCCGGAGGCG
Bd_09-1     GCGTGCTACA ATATCTATTT TCAAGAGCAC TTTAAACTGC TCATAGGAGA CCATTGCCTC CCCGGAGGCG
Bd_09-2     GCGTGCTACA ATATCTATTT TCAAGAGCAC TTTAAACTGC TCATAGGAGA CCATTGCCTC CCCGGAGGCG
Bd_09-3     GCGTGCTACA ATATCTATTT TCAAGAGCAC TTTAAACTGC TCATAGGAGA CCATTGCCTC CCCGGAGGCG
Bd_10-1     GCGTGCTACA ATATCTATTT TCAAGAGCAC TTTAAACTGC TCATAGGAGA CCATTGCCTC CCCGGAGGCG
Bd_10-2     GCGTGCTACA ATATCTATTT TCAAGAGCAC TTTAAACTGC TCATAGGAGA CCATTGCCTC CCCGGAGGCG
Bd_10-2b    GCGTGCTACA ATATCTATTT TCAAGAGCAC TTTAAACTGC TCATAGGAGA CCATTGCCTC CCCGGAGGCG
Bd_11-2     GCGTGCTACA ATATCTATTT TCAAGAGCAC TTTAAACTGC TCATAGGAGA CCATTGCCTC CCCGGAGGCG
Bd_11-6     GCGTGCTACA ATATCTATTT TCAAGAGCAC TTTAAACTGC TCATAGGAGA CCATTGCCTC CCCGGAGGCG
Bd_12-2     GCGTGCTACA ATATCTATTT TCAAGAGCAC TTTAAACTGC TCATAGGAGA CCATTGCCTC CCCGGAGGCG
Bd_13-1     GCGTGCTACA ATATCTATTT TCAAGAGCAC TTTAAACTGC TCATAGGAGA CCATTGCCTC CCCGGAGGCG
Bd_13-4     GCGTGCTACA ATATCTATTT TCAAGAGCAC TTTAAACTGC TCATAGGAGA CCATTGCCTC CCCGGAGGCG
Bd_13-5     GCGTGCTACA ATATCTATTT TCAAGAGCAC TTTAAACTGC TCATAGGAGA CCATTGCCTC CCCGGAGGCG
Bd_14-1     GCGTGCTACA ATATCTATTT TCAAGAGCAC TTTAAACTGC TCATAGGAGA CCATTGCCTC CCCGGAGGCG
Bd_14-2     GCGTGCTACA ATATCTATTT TCAAGAGCAC TTTAAACTGC TCATAGGAGA CCATTGCCTC CCCGGAGGCG
Bd_19-5     GCGTGCTACA ATATCTATTT TCAAGAGCAC TTTAAACTGC TCATAGGAGA CCATTGCCTC CCCGGAGGCG
Bd_19-6     GCGTGCTACA ATATCTATTT TCAAGAGCAC TTTAAACTGC TCATAGGAGA CCATTGCCTC CCCGGAGGCG
Bd_20-1     GCGTGCTACA ATATCTATTT TCAAGAGCAC TTTAAACTGC TCATAGGAGA CCATTGCCTC CCCGGAGGCG
Bd_20-2     GCGTGCTACA ATATCTATTT TCAAGAGCAC TTTAAACTGC TCATAGGAGA CCATTGCCTC CCCGGAGGCG
Bd_22-1     GCGTGCTACA ATATCTATTT TCAAGAGCAC TTTAAACTGC TCATAGGAGA CCATTGCCTC CCCGGAGGCG
Go_06-1     GCGTGCTACA ATAGCTATCA TCCAGAGTAC -ACACACGGC CGATAGGGAA CG---AACTC TCTAACGAGG
Go_06-9     GCGTGCTACA ATAGCTATCA TCCAGAGTAC -ACACACGGC CGATAGGGAA CG---AACTC TCTAACGAGG
Go_07-1     GCGTGCTACA ATAGCTATCA TCCAGAGTAC -ACACACGGC CGATAGGGAA CG---AACTC TCTAACGAGG
Go_07-5     GCGTGCTACA ATAGCTATCA TCCAGAGTAC -ACACACAGT CGATAGGGAA CG---AACTC TCTAACGAGG
Go_07-6     GCGTGCTACA ATAGCTATCA TCCAGAGTAC -ACACACGGC CGATAGGGAA CG---AACTC TCTAACGAGG
Go_07-8     GCGTGCTACA ATAGCTATCA TCCAGAGTAC -ACACACGGC CGATAGGGAA CG---AACTC TCTAACGAGG
Go_09-2     GCGTGCTACA ATATCTATTT TCAAGAGCAC TTTAAACTGC TCATAGGAGA CCATTGCCTC CCCGGAGGCG
Go_09-3     GCGTGCTACA ATATCTATTT TCAAGAGCAC TTTAAACTGC TCATAGGAGA CCATTGCCTC CCCGGAGGCG
Go_09-4     GCGTGCTACA ATATCTATTT TCAAGAGCAC TTTAAACTGC TCATAGGAGA CCATTGCCTC CCCGGAGGCG
Go_10-1     GCGTGCTACA ATAGCTATCA TCCAGAGTAC -ACACACGGC CGATAGGGAA CG---AACTC TCTAACGAGG
Go_10-3     GCGTGCTACA ATAGCTATCA TCCAGAGTAC -ACACACAGT CGATAGGGAA CG---AACTC TCTAACGAGG
Go_11-3     GCGTGCTACA ATAGCTATCA TCCAGAGTAC -ACACACGGC CGATAGGGAA CG---AACTC TCTAACGAGG
Go_11-5     GCGTGCTACA ATAGCTATCA TCCAGAGTAC -ACACACAGT CGATAGGGAA CG---AACTC TCTAACGAGG
Go_13-5     GCGTGCTACA ATAGCTATCA TCCAGAGTAC -ACACACGGC CGATAGGGAA CG---AACTC TCTAACGAGG
Go_14-2     GCGTGCTACA ATAGCTATCA TCCAGAGTAC -ACACACGGC CGATAGGGAA CG---AACTC TCTAACGAGG
Go_14-3     GCGTGCTACA ATAGCTATCA TCCAGAGTAC -ACACACGGC CGATAGGGAA CG---AACTC TCTAACGAGG
Go_14-4     GCGTGCTACA ATAGCTATCA TCCAGAGTAC -ACACACGGC CGATAGGGAA CG---AACTC TCTAACGAGG
Pa_06-2     GCGTGCTACA ATATCTATTT TCAAGAGTAC TTTTAACATT CCATAGGTGG ATGTTGGCTT TCACGAGCTG
Pa_14-4     GCGTGCTACA ATATCTATTT TCCAGAGTAC TTTTAGCAGT TTATAGGGCA CGAGTGGCTC TCACGAGCCA
Pa_14-6     GCGTGCTACA ATATCTATTT TCCAGAGTAC TTTTAGCAGT TTATAGGGCA CGAGTGGCTC TCACGAGCCA
Pa_16-1     GCGTGCTACA ATATCTATTT TCAAGAGTAC ACAGAGCAGT CAATAGGACC GGTTTGGCTC TCACGAGCTG
Pa_19-1     GCGTGCTACA ATATCTATTT TCAAGAGTAC -ATCAACAGT CAATAGGAAG CAGTAGGCTC TCGCGAGCTT
Pa_19-2     GCGTGCTACA ATATCTATTT TCAAGAGTAC -ATCAACAGT CAATAGGAAG CAGTAGGCTC TCGCGAGCTT
Pa_19-3     GCGTGCTACA ATATCTATTT TCAAGAGTAC -ATCAACAGT CAATAGGAAG CAGTAGGCTC TCGCGAGCTT
Pa_21-2     GCGTGCTACA ATATCTATTT TCAAGAGTAC TTTTAGCAGT GTATAGGGCA TGTTTGGCTC TCACGAGCCG
Pa_22-3     GCGTGCTACA ATATCTATTT TCAAGAGTAC TTTTAGCAGT GTATAGGGCA TGTTTGGCTC TCACGAGCCG
Pa_39-1     GCGTGCTACA ATATCTATTT TCAAGAGTAC TCTTAGCAGT GGATAGGGCA TGGTTGGCTC TCACGAGCCG
Pa_39-5     GCGTGCTACA ATATCTATTT TCAAGAGTAC TCTTAGCAGT GGATAGGGCA TGGTTGGCTC TCACGAGCCG
Pa_50-11    GCGTGCTACA ATATCTATTT TCAAGAGTAC TTTTAACATT CCATAGGTGG ATGTTGGCTT TCACGAGCTG
Pa_50-12    GCGTGCTACA ATATCTATTT TCAAGAGTAC TTTTAACATT CCATAGGTGG ATGTTGGCTT TCACGAGCTG
Pa_50-19    GCGTGCTACA ATATCTATTT TCAAGAGTAC TTTTAACATT CCATAGGTGG ATGTTGGCTT TCACGAGCTG
Pa_50-2     GCGTGCTACA ATATCTATTT TCAAGAGTAC TTTTAACATT CCATAGGTGG ATGTTGGCTT TCACGAGCTG
Pa_57-2     GCGTGCTACA ATATCTATTT TCAAGAGTAC TTTTAACATT CCATAGGTGG ATGTTGGCTT TCACGAGCTG
Pa_57-3     GCGTGCTACA ATATCTATTT TCAAGAGTAC TTTTAACATT CCATAGGTGG ATGTTGGCTT TCACGAGCTG
Pa_57-5     GCGTGCTACA ATATCTATTT TCAAGAGTAC TTTTAACATT CCATAGGTGG ATGTTGGCTT TCACGAGCTG
Pa_62-19    GCGTGCTACA ATATCTATTT TCAAGAGTAC -ATCAACAGT CAATAGGAGA CAGTAGGCTC TCGCGAGCTT
Pa_62-3     GCGTGCTACA ATATCTATTT TCAAGAGTAC -ATCAACAGT CAATAGGAGA CAGTAGGCTC TCGCGAGCTT
Pa_63-2     GCGTGCTACA ATATCTATTT TCAAGAGCAC TTTAAACTGC TGATAGGAGG TTGTTGACTC CACGGAGGCG
Pa_63-3     GCGTGCTACA ATATCTATTT TCAAGAGCAC TTTAAACTGC TGATAGGAGG TTGTTGACTC CACGGAGGCG
Pa_63-4     GCGTGCTACA ATATCTATTT TCCAGAGTAC TTTTAACAGC GGATAGGGCA CTGTTGGCTC TCACGAGCTG
Pa_64-1     GCGTGCTACA ATATCTATTT TCAAGAGTAC AATGAACAGC CCATAGGTGT TGCGTGGCTC TCACGAGCTG
Pa_64-2     GCGTGCTACA ATATCTATTT TCAAGAGTAC AATGAACAGC CCATAGGTGT TGCGTGGCTC TCACGAGCTG
Pa_64-3     GCGTGCTACA ATATCTATTT TCAAGAGTAC AATGAACAGC CCATAGGTGT TGCGTGGCTC TCACGAGCTG
Pa_64-4     GCGTGCTACA ATATCTATTT TCAAGAGTAC AATGAACAGC CCATAGGTGT TGCGTGGCTC TCACGAGCTG
Pa_79-4     GCGTGCTACA ATATCTATTT TCAAGAGTAC -ATGAACAGT CGATAGGAGG CAATTGGCTC TCGCGAGCTG
```

1611

Bd_06-10	ACTGGTCTCA	ACTAGGGCTG	TTGACCTTGT	CCGAAAGGAT	TGGGGAAACT	CGTAAGATAG	ATGTAACAGG
Bd_09-1	ACTGGTCTCA	ACTAGGGCTG	TTGACCTTGT	CCGAAAGGAT	TGGGGAAACT	CGTAAGATAG	ATGTAACAGG
Bd_09-2	ACTGGTCTCA	ACTAGGGCTG	TTGACCTTGT	CCGAAAGGAT	TGGGGAAACT	CGTAAGATAG	ATGTAACAGG
Bd_09-3	ACTGGTCTCA	ACTAGGGCTG	TTGACCTTGT	CCGAAAGGAT	TGGGGAAACT	CGTAAGATAG	ATGTAACAGG
Bd_10-1	ACTGGTCTCA	ACTAGGGCTG	TTGACCTTGC	CCGAAAGGAT	TGGGGAAACT	CGTAAGATAG	ATGTAACAGG
Bd_10-2	ACTGGTCTCA	ACTAGGGCTG	TTGACCTTGT	CCGAAAGGAT	TGGGGAAACT	CGTAAGATAG	ATGTAACAGG
Bd_10-2b	ACTGGTCTCA	ACTAGGGCTG	TTGACCTTGT	CCGAAAGGAT	TGGGGAAACT	CGTAAGATAG	ATGTAACAGG
Bd_11-2	ACTGGTCTCA	ACTAGGGCTG	TTGACCTTGT	CCGAAAGGAT	TGGGGAAACT	CGTAAGATAG	ATGTAACAGG
Bd_11-6	ACTGGTCTCA	ACTAGGGCTG	TTGACCTTGT	CCGAAAGGAT	TGGGGAAACT	CGTAAGATAG	ATGTAACAGG
Bd_12-2	ACTGGTCTCA	ACTAGGGCTG	TTGACCTTGT	CCGAAAGGAT	TGGGGAAACT	CGTAAGATAG	ATGTAACAGG
Bd_13-1	ACTGGTCTCA	ACTAGGGCTG	TTGACCTTGT	CCGAAAGGAT	TGGGGAAACT	CGTAAGATAG	ATGTAACAGG
Bd_13-4	ACTGGTCTCA	ACTAGGGCTG	TTGACCTTGT	CCGAAAGGAT	TGGGGAAACT	CGTAAGATAG	ATGTAACAGG
Bd_13-5	ACTGGTCTCA	ACTAGGGCTG	TTGACCTTGT	CCGAAAGGAT	TGGGGAAACT	CGTAAGATAG	ATGTAACAGG
Bd_14-1	ACTGGTCTCA	ACTAGGGCTG	TTGACCTTGT	CCGAAAGGAT	TGGGGAAACT	CGTAAGATAG	ATGTAACAGG
Bd_14-2	ACTGGTCTCA	ACTAGGGCTG	TTGACCTTGT	CCGAAAGGAT	TGGGGAAACT	CGTAAGATAG	ATGTAACAGG
Bd_19-5	ACTGGTCTCA	ACTAGGGCTG	TTGACCTTGT	CCGAAAGGAT	TGGGGAAACT	CGTAAGATAG	ATGTAACAGG
Bd_19-6	ACTGGTCTCA	ACTAGGGCTG	TTGACCTTGT	CCGAAAGGAT	TGGGGAAACT	CGTAAGATAG	ATGTAACAGG
Bd_20-1	ACTGGTCTCA	ACTAGGGCTG	TTGACCTTGT	CCGAAAGGAT	TGGGGAAACT	CGTAAGATAG	ATGTAACAGG
Bd_20-2	ACTGGTCTCA	ACTAGGGCTG	TTGACCTTGT	CCGAAAGGAT	TGGGGAAACT	CGTAAGATAG	ATGTAACAGG
Bd_22-1	ACTGGTCTCA	ACTAGGGCTG	TTGACCTTGT	CCGAAAGGAT	TGGGGAAACT	CGTAAGATAG	ATGTAACAGG
Go_06-1	GGC-GGTCCA	ACTAAGGCCG	AAAACCCAAT	CCGAAAGGAG	GCGGAAAACT	CGTAAGGTAG	CTGTAACAGG
Go_06-9	GGC-GGTCCA	ACTAAGGCCG	AAAACCCAAT	CCGAAAGGAG	GCGGAAAACT	CGTAAGGTAG	CTGTAACAGG
Go_07-1	GGC-GGTCCA	ACTAAGGCCG	AAAACCCAAT	CCGAAAGGAG	GCGGAAAACT	CGTAAGGTAG	CTGTAACAGG
Go_07-5	GGC-GGTCCA	ACTAAGACTG	AAAACCCAAT	CCGAAAGGAG	GCGGAAAACT	CGTAAGGTAG	CTGTAACAGG
Go_07-6	GGC-GGTCCA	ACTAAGGCCG	AAAACCCAAT	CCGAAAGGAG	GCGGAAAACT	CGTAAGGTAG	CTGTAACAGG
Go_07-8	GGC-GGTCCA	ACTAAGGCCG	AAAACCCAAT	CCGAAAGGAG	GCGGAAAACT	CGTAAGGTAG	CTGTAACAGG
Go_09-2	ACTGGTCTCA	ACTAGGGCTG	TTGACCTTGT	CCGAAAGGAT	TGGGGAAACT	CGTAAGATAG	ATGTAACAGG
Go_09-3	ACTGGTCTCA	ACTAGGGCTG	TTGACCTTGT	CCGAAAGGAT	TGGGGAAACT	CGTAAGATAG	ATGTAACAGG
Go_09-4	ACTGGTCTCA	ACTAGGGCTG	TTGACCTTGT	CCGAAAGGAT	TGGGGAAACT	CGTAAGATAG	ATGTAACAGG
Go_10-1	GGC-GGTCCA	ACTAAGGCCG	AAAACCCAAT	CCGAAAGGAG	GCGGAAAACT	CGTAAGGTAG	CTGTAACAGG
Go_10-3	GGC-GGTCCA	ACTAAGACTG	AAAACCCAAT	CCGAAAGGAG	GCGGAAAACT	CGTAAGGTAG	CTGTAACAGG
Go_11-3	GGC-GGTCCA	ACTAAGGCCG	AAAACCCAAT	CCGAAAGGAG	GCGGAAAACT	CGTAAGGTAG	CTGTAACAGG
Go_11-5	GGC-GGTCCA	ACTAAGACTG	AAAACCCAAT	CCGAAAGGAG	GCGGAAAACT	CGTAAGGTAG	CTGTAACAGG
Go_13-5	GGC-GGTCCA	ACTAAGGCCG	AAAACCCAAT	CCGAAAGGAG	GCGGAAAACT	CGTAAGGTAG	CTGTAACAGG
Go_14-2	GGC-GGTCCA	ACTAAGGCCG	AAAACCCAAT	CCGAAAGGAG	GCGGAAAACT	CGTAAGGTAG	CTGTAACAGG
Go_14-3	GGC-GGTCCA	ACTAAGGCCG	AAAACCCAAT	CCGAAAGGAG	GCGGAAAACT	CGTAAGGTAG	CTGTAACAGG
Go_14-4	GGC-GGTCCA	ACTAAGGCCG	AAAACCCAAT	CCGAAAGGAG	GCGGAAAACT	CGTAAGGTAG	CTGTAACAGG
Pa_06-2	ACA-GACGCA	ACTAGGTTTG	TCTACCTTTT	CCGAAAGGAT	TGGGAAAACT	CGTAAGATAG	ATGTAACAGG
Pa_14-4	GTC-GAGTCA	ACTAGAACTG	TGTACCTTCT	CCGAAAGGAG	CGGGAAAACT	CGTAAGATAG	ATGTAACAGA
Pa_14-6	GTC-GAGTCA	ACTAGAACTG	TGTACCTTCT	CCGAAAGGAG	CGGGAAAACT	CGTAAGATAG	ATGTAACAGG
Pa_16-1	AGT-CAGTCG	ACTAGGGCTG	CGTACCTTCA	CCGAAAGGAG	TGGGAAAACT	CGTAAGATAG	ATGTAACAGG
Pa_19-1	AGT-GTTTTG	ACTAGGGCTG	AACACCTTTT	CCGAAAGGAG	TGGGAAAACT	CGTAAGATAG	ATGTAACAGG
Pa_19-2	AGT-GTTTTG	ACTAGGGCTG	AACACCTTTT	CCGAAAGGAG	TGGGAAAACT	CGTAAGATAG	ATGTAACAGG
Pa_19-3	AGT-GTTTTG	ACTAGGGCTG	AACACCTTTT	CCGAAAGGAG	TGGGAAAACT	CGTAAGATAG	ATGTAACAGG
Pa_21-2	AGT-GCGCCA	ACTAGCACTG	TGTACCTTCT	CCGAAAGGAG	CGGGAAAACT	CGTAAGATAG	ATGTAACAGG
Pa_22-3	AGT-GCGCCA	ACTAGCACTG	TGTACCTTCT	CCGAAAGGAG	CGGGAAAACT	CGTAAGATAG	ATGTAACAGG
Pa_39-1	AGT-GCGCCA	ACTAGCACTG	TGTACCTTCT	CCGAAAGGAG	CGGGAAAACT	CGTAAGATAG	ATGTAACAGG
Pa_39-5	AGT-GCGCCA	ACTAGCACTG	TGTACCTTCT	CCGAAAGGAG	CGGGAAAACT	CGTAAGATAG	ATGTAACAGG
Pa_50-11	ACA-GACGCA	ACTAGGTTTG	TCTACCTTTT	CCGAAAGGAT	TGGGAAAACT	CGTAAGATAG	ATGTAACAGG
Pa_50-12	ACA-GACGCA	ACTAGGTTTG	TCTACCTTTT	CCGAAAGGAT	TGGGAAAACT	CGTAAGATAG	ATGTAACAGG
Pa_50-19	ACA-GACGCA	ACTAGGTTTG	TCTACCTTTT	CCGAAAGGAT	TGGGAAAACT	CGTAAGATAG	ATGTAACAGG
Pa_50-2	ACA-GACGCA	ACTAGGTTTG	TCTACCTTTT	CCGAAAGGAT	TGGGAAAACT	CGTAAGATAG	ATGTAACAGG
Pa_57-2	ACA-GACGCA	ACTAGGTTTG	TCTACCTTTT	CCGAAAGGAT	TGGGAAAACT	CGTAAGATAG	ATGTAACAGG
Pa_57-3	ACA-GACGCA	ACTAGGTTTG	TCTACCTTTT	CCGAAAGGAT	TGGGAAAACT	CGTAAGATAG	ATGTAACAGG
Pa_57-5	ACA-GACGCA	ACTAGGTTTG	TCTACCTTTT	CCGAAAGGAT	TGGGAAAACT	CGTAAGATAG	ATGTAACAGG
Pa_62-19	AGT-GTTTCG	ACTAGGGCTG	AACACCTTTT	CCGAAAGGAG	TGGGAAAACT	CGTAAGATAG	ATGTAACAGG
Pa_62-3	AGT-GTTTCG	ACTAGGGCTG	AACACCTTTT	CCGAAAGGAG	TGGGAAAACT	CGTAAGATAG	ATGTAACAGG
Pa_63-2	ATTGATCTCA	ACTAGAGCTG	TTGACCTTTT	CCGAAAGGAC	TGGGGAAACT	CGTAAGATAG	ATGTAACAGG
Pa_63-3	ATTGATCTCA	ACTAGAGCTG	TTGACCTTTT	CCGAAAGGAC	TGGGGAAACT	CGTAAGATAG	ATGTAACAGG
Pa_63-4	ACC-GAGCCG	ACTAGCGCTG	TGTACCTTCT	CCGAAAGGAG	CGGGAAAACT	CGTAAGATAG	ATGTAACAGG
Pa_64-1	CGG-GACGCA	ACTAGGACTG	TGTACCTTTT	CCGAAAGGAG	TGGGAAAACT	CGTAAGATAG	ATGTAACAGG
Pa_64-2	CGG-GACGCA	ACTAGGACTG	TGTACCTTTT	CCGAAAGGAG	TGGGAAAACT	CGTAAGATAG	ATGTAACAGG
Pa_64-3	CGG-GACGCA	ACTAGGACTG	TGTACCTTTT	CCGAAAGGAG	TGGGAAAACT	CGTAAGATAG	ATGTAACAGG
Pa_64-4	CGG-GACGCA	ACTAGGACTG	TGTACCTTTT	CCGAAAGGAG	TGGGAAAACT	CGTAAGATAG	ATGTAACAGG
Pa_79-4	ATT-GCCTCA	ACTAAGGCTG	AGAACCTTTT	CCGAAAGGAG	TGGGAAAACT	CGTAAGATAG	ATGTAACAGG

	1681						
Bd_06-10	GATTAATGAT	TGGAATTATT	TGTTATGGAA	CGAGGAATTC	CTTGTAAAAT	TTTGTCACTA	ACGAAAGTTG
Bd_09-1	GATTAATGAT	TGGAATTATT	TGTTATGGAA	CGAGGAATTC	CTTGTAAAAT	TTTGTCACTA	ACGAAAGTTG
Bd_09-2	GATTAATGAT	TGGAATTATT	TGTTATGGAA	CGAGGAATTC	CTTGTAAAAT	TTTGTCACTA	ACGAAAGTTG
Bd_09-3	GATTAATGAT	TGGAATTATT	TGTTATGGAA	CGAGGAATTC	CTTGTAAAAT	TTTGTCACTA	ACGAAAGTTG
Bd_10-1	GATTAATGAT	TGGAATTATT	TGTTATGGAA	CGAGGAATTC	CTTGTAAAAT	TTTGTCACTA	ACGAAAGTTG
Bd_10-2	GATTAATGAT	TGGAATTATT	TGTTATGGAA	CGAGGAATTC	CTTGTAAAAT	TTTGTCACTA	ACGAAAGTTG
Bd_10-2b	GATTAATGAT	TGGAATTATT	TGTTATGGAA	CGAGGAATTC	CTTGTAAAAT	TTTGTCACTA	ACGAAAGTTG
Bd_11-2	GATTAATGAT	TGGAATTATT	TGTTATGGAA	CGAGGAATTC	CTTGTAAAAT	TTTGTCACTA	ACGAAAGTTG
Bd_11-6	GATTAATGAT	TGGAATTATT	TGTTATGGAA	CGAGGAATTC	CTTGTAAAAT	TTTGTCACTA	ACGAAAGTTG
Bd_12-2	GATTAATGAT	TGGAATTATT	TGTTATGGAA	CGAGGAATTC	CTTGTAAAAT	TTTGTCACTA	ACGAAAGTTG
Bd_13-1	GATTAATGAT	TGGAATTATT	TGTTATGGAA	CGAGGAATTC	CTTGTAAAAT	TTTGTCACTA	ACGAAAGTTG
Bd_13-4	GATTAATGAT	TGGAATTATT	TGTTATGGAA	CGAGGAATTC	CTTGTAAAAT	TTTGTCACTA	ACGAAAGTTG
Bd_13-5	GATTAATGAT	TGGAATTATT	TGTTATGGAA	CGAGGAATTC	CTTGTAAAAT	TTTGTCACTA	ACGAAAGTTG
Bd_14-1	GATTAATGAT	TGGAATTATT	TGTTATGGAA	CGAGGAATTC	CTTGTAAAAT	TTTGTCACTA	ACGAAAGTTG
Bd_14-2	GATTAATGAT	TGGAATTATT	TGTTATGGAA	CGAGGAATTC	CTTGTAAAAT	TTTGTCACTA	ACGAAAGTTG
Bd_19-5	GATTAATGAT	TGGAATTATT	TGTTATGGAA	CGAGGAATTC	CTTGTAAAAT	TTTGTCACTA	ACGAAAGTTG
Bd_19-6	GATTAATGAT	TGGAATTATT	TGTTATGGAA	CGAGGAATTC	CTTGTAAAAT	TTTGTCACTA	ACGAAAGTTG
Bd_20-1	GATTAATGAT	TGGAATTATT	TGTTATGGAA	CGAGGAATTC	CTTGTAAAAT	TTTGTCACTA	ACGAAAGTTG
Bd_20-2	GATTAATGAT	TGGAATTATT	TGTTATGGAA	CGAGGAATTC	CTTGTAAAAT	TTTGTCACTA	ACGAAAGTTG
Bd_22-1	GATTAATGAT	TGGAATTATT	TGTTATGGAA	CGAGGAATTC	CTTGTAAAAT	TTTGTCACTA	ACGAAAGTTG
Go_06-1	GATTAATGAT	TGGAATTATT	TGTTATGGAA	CGAGGAATTC	CTTGTAAAAT	TTTGTCACTA	ACGAAAGTTG
Go_06-9	GATTAATGAT	TGGAATTATT	TGTTATGGAA	CGAGGAATTC	CTTGTAAAAT	TTTGTCACTA	ACGAAAGTTG
Go_07-1	GATTAATGAT	TGGAATTATT	TGTTATGGAA	CGAGGAATTC	CTTGTAAAAT	TTTGTCACTA	ACGAAAGTTG
Go_07-5	GATTAATGAT	TGGAATTATT	TGTTATGGAA	CGAGGAATTC	CTTGTAAAAT	TTTGTCACTA	ACGAAAGTTG
Go_07-6	GATTAATGAT	TGGAATTATT	TGTTATGGAA	CGAGGAATTC	CTTGTAAAAT	TTTGTCACTA	ACGAAAGTTG
Go_07-8	GATTAATGAT	TGGAATTATT	TGTTATGGAA	CGAGGAATTC	CTTGTAAAAT	TTTGTCACTA	ACGAAAGTTG
Go_09-2	GATTAATGAT	TGGAATTATT	TGTTATGGAA	CGAGGAATTC	CTTGTAAAAT	TTTGTCACTA	ACGAAAGTTG
Go_09-3	GATTAATGAT	TGGAATTATT	TGTTATGGAA	CGAGGAATTC	CTTGTAAAAT	TTTGTCACTA	ACGAAAGTTG
Go_09-4	GATTAATGAT	TGGAATTATT	TGTTATGGAA	CGAGGAATTC	CTTGTAAAAT	TTTGTCACTA	ACGAAAGTTG
Go_10-1	GATTAATGAT	TGGAATTATT	TGTTATGGAA	CGAGGAATTC	CTTGTAAAAT	TTTGTCACTA	ACGAAAGTTG
Go_10-3	GATTAATGAT	TGGAATTATT	TGTTATGGAA	CGAGGAATTC	CTTGTAAAAT	TTTGTCACTA	ACGAAAGTTG
Go_11-3	GATTAATGAT	TGGAATTATT	TGTTATGGAA	CGAGGAATTC	CTTGTAAAAT	TTTGTCACTA	ACGAAAGTTG
Go_11-5	GATTAATGAT	TGGAATTATT	TGTTATGGAA	CGAGGAATTC	CTTGTAAAAT	TTTGTCACTA	ACGAAAGTTG
Go_13-5	GATTAATGAT	TGGAATTATT	TGTTATGGAA	CGAGGAATTC	CTTGTAAAAT	TTTGTCACTA	ACGAAAGTTG
Go_14-2	GATTAATGAT	TGGAATTATT	TGTTATGGAA	CGAGGAATTC	CTTGTAAAAT	TTTGTCACTA	ACGAAAGTTG
Go_14-3	GATTAATGAT	TGGAATTATT	TGTTATGGAA	CGAGGAATTC	CNTGTAAAAT	TTTGTCACTA	ACGAAAGTTG
Go_14-4	GATTAATGAT	TGGAATTATT	TGTTATGGAA	CGAGGAATTC	CTTGTAAAAT	TTTGTCACTA	ACGAAAGTTG
Pa_06-2	GATTAATGAT	TGGAATTATT	TGTTATGGAA	CGAGGAATTC	CTTGTAAAAT	TTTGTCACTA	ACGAAAGTTG
Pa_14-4	GATTAATGAT	TGGAATTATT	TGTTATGTAA	CGAGGAATTC	CTTGTAAAAT	TTTGTCACTA	ACGAAAGTTG
Pa_14-6	GATTAATGAT	TGGAATTATT	TGTTATGTAA	CGAGGAATTC	CTTGTAAAAT	TTTGTCACTA	ACGAAAGTTG
Pa_16-1	GATTAATGAT	TGGAATTATT	TGTTATGGAA	CGAGGAATTC	CTTGTAAAAT	TTTGTCACTA	ACGAAAGTTG
Pa_19-1	GATTAATGAT	TGGAATTATT	TGTTATGGAA	CGAGGAATTC	CTTGTAAAAT	TTTGTCACTA	ACGAAAGTTG
Pa_19-2	GATTAATGAT	TGGAATTATT	TGTTATGGAA	CGAGGAATTC	CTTGTAAAAT	TTTGTCACTA	ACGAAAGTTG
Pa_19-3	GATTAATGAT	TGGAATTATT	TGTTATGGAA	CGAGGAATTC	CTTGTAAAAT	TTTGTCACTA	ACGAAAGTTG
Pa_21-2	GATTAATGAT	TGGAATTATT	TGTTATGTAA	CGAGGAATTC	CTTGTAAAAT	TTTGTCACTA	GCGAAAGTTG
Pa_22-3	GATTAATGAT	TGGAATTATT	TGTTATGTAA	CGAGGAATTC	CTTGTAAAAT	TTTGTCACTA	GCGAAAGTTG
Pa_39-1	GATTAATGAT	TGGAATTATT	TGTTATGTAA	CGAGGAATTC	CTTGTAAAAT	TTTGTCACTA	ACGAAAGTTG
Pa_39-5	GATTAATGAT	TGGAATTATT	TGTTATGTAA	CGAGGAATTC	CTTGTAAAAT	TTTGTCACTA	ACGAAAGTTG
Pa_50-11	GATTAATGAT	TGGAATTATT	TGTTATGGAA	CGAGGAATTC	CTTGTAAAAT	TTTGTCACTA	ACGAAAGTTG
Pa_50-12	GATTAATGAT	TGGAATTATT	TGTTATGGAA	CGAGGAATTC	CTTGTAAAAT	TTTGTCACTA	ACGAAAGTTG
Pa_50-19	GATTAATGAT	TGGAATTATT	TGTTATGGAA	CGAGGAATTC	CTTGTAAAAT	TTTGTCACTA	ACGAAAGTTG
Pa_50-2	GATTAATGAT	TGGAATTATT	TGTTATGGAA	CGAGGAATTC	CTTGTAAAAT	TTTGTCACTA	ACGAAAGTTG
Pa_57-2	GATTAATGAT	TGGAATTATT	TGTTATGGAA	CGAGGAATTC	CTTGTAAAAT	TTTGTCACTA	ACGAAAGTTG
Pa_57-3	GATTAATGAT	TGGAATTATT	TGTTATGGAA	CGAGGAATTC	CTTGTAAAAT	TTTGTCACTA	ACGAAAGTTG
Pa_57-5	GATTAATGAT	TGGAATTATT	TGTTATGGAA	CGAGGAATTC	CTTGTAAAAT	TTTGTCACTA	ACGAAAGTTG
Pa_62-19	GATTAATGAT	TGGAATTATT	TGTTATGGAA	CGAGGAATTC	CTTGTAAAAT	TTTGTCACTA	ACGAAAGTTG
Pa_62-3	GATTAATGAT	TGGAATTATT	TGTTATGGAA	CGAGGAATTC	CTTGTAAAAT	TTTGTCACTA	ACGAAAGTTG
Pa_63-2	GATTAATGAT	TGGAATTATT	TGTTATGGAA	CGAGGAATTC	CTTGTAAAAT	TTTGTCACTA	ACGAAAGTTG
Pa_63-3	GATTAATGAT	TGGAATTATT	TGTTATGGAA	CGAGGAATTC	CTTGTAAAAT	TTTGTCACTA	ACGAAAGTTG
Pa_63-4	GATTAATGAT	TGGAATTATT	TGTTATGTAA	CGAGGAATTC	CTTGTAAAAT	TTTGTCACTA	ACGAAAGTTG
Pa_64-1	GATTAATGAT	TGGAATTATT	TGTTATGTAA	CGAGGAATTC	CTTGTAAAAT	TTTGTCACTA	ACGAAAGTTG
Pa_64-2	GATTAATGAT	TGGAATTATT	TGTTATGTAA	CGAGGAATTC	CTTGTAAAAT	TTTGTCACTA	ACGAAAGTTG
Pa_64-3	GATTAATGAT	TGGAATTATT	TGTTATGTAA	CGAGGAATTC	CTTGTAAAAT	TTTGTCACTA	ACGAAAGTTG
Pa_64-4	GATTAATGAT	TGGAATTATT	TGTTATGTAA	CGAGGAATTC	CTTGTAAAAT	TTTGTCACTA	ACGAAAGTTG
Pa_79-4	GATTAATGAT	TGGAATTATT	TGTTATGGAA	CGAGGAATTC	CTTGTAAAAT	TTTGTCACTA	ACGAAAGTTG

1751

Bd_06-10	AATACGTCCC	TGCCCTTTGT	ACACACCGCC	CGTCGCTCCT	ACCGATTGAA	TAAAAAGATG	AAGGTTTAGG
Bd_09-1	AATACGTCCC	TGCCCTTTGT	ACACACCGCC	CGTCGCTCCT	ACCGATTGAA	TAAAAAGATG	AAGGTTTAGG
Bd_09-2	AATACGTCCC	TGCCCTTTGT	ACACACCGCC	CGTCGCTCCT	ACCGATTGAA	TAAAAAGATG	AAGGTTTAGG
Bd_09-3	AATACGTCCC	TGCCCTTTGT	ACACACCGCC	CGTCGCTCCT	ACCGATTGAA	TAAAAAGATG	AAGGTTTAGG
Bd_10-1	AATACGTCCC	TGCCCTTTGT	ACACACCGCC	CGTCGCTCCT	ACCGATTGAA	TAAAAAGATG	AAGGTTTAGG
Bd_10-2	AATACGTCCC	TGCCCTTTGT	ACACACCGCC	CGTCGCTCCT	ACCGATTGAA	TAAAAAGATG	AAGGTTTAGG
Bd_10-2b	AATACGTCCC	TGCCCTTTGT	ACACACCGCC	CGTCGCTCCT	ACCGATTGAA	TAAAAAGATG	AAGGTTTAGG
Bd_11-2	AATACGTCCC	TGCCCTTTGT	ACACACCGCC	CGTCGCTCCT	ACCGATTGAA	TAAAAAGATG	AAGGTTTAGG
Bd_11-6	AATACGTCCC	TGCCCTTTGT	ACACACCGCC	CGTCGCTCCT	ACCGATTGAA	TAAAAAGATG	AAGGTTTAGG
Bd_12-2	AATACGTCCC	TGCCCTTTGT	ACACACCGCC	CGTCGCTCCT	ACCGATTGAA	TAAAAAGATG	AAGGTTTAGG
Bd_13-1	AATACGTCCC	TGCCCTTTGT	ACACACCGCC	CGTCGCTCCT	ACCGATTGAA	TAAAAAGATG	AAGGTTTAGG
Bd_13-4	AATACGTCCC	TGCCCTTTGT	ACACACCGCC	CGTCGCTCCT	ACCGATTGAA	TAAAAAGATG	AAGGTTTAGG
Bd_13-5	AATACGTCCC	TGCCCTTTGT	ACACACCGCC	CGTCGCTCCT	ACCGATTGAA	TAAAAAGATG	AAGGTTTAGG
Bd_14-1	AATACGTCCC	TGCCCTTTGT	ACACACCGCC	CGTCGCTCCT	ACCGATTGAA	TAAAAAGATG	AAGGTTTAGG
Bd_14-2	AATACGTCCC	TGCCCTTTGT	ACACACCGCC	CGTCGCTCCT	ACCGATTGAA	TAAAAAGATG	AAGGTTTAGG
Bd_19-5	AATACGTCCC	TGCCCTTTGT	ACACACCGCC	CGTCGCTCCT	ACCGATTGAA	TAAAAAGATG	AAGGTTTAGG
Bd_19-6	AATACGTCCC	TGCCCTTTGT	ACACACCGCC	CGTCGCTCCT	ACCGATTGAA	TAAAAAGATG	AAGGTTTAGG
Bd_20-1	AATACGTCCC	TGCCCTTTGT	ACACACCGCC	CGTCGCTCCT	ACCGATTGAA	TAAAAAGATG	AAGGTTTAGG
Bd_20-2	AATACGTCCC	TGCCCTTTGT	ACACACCGCC	CGTCGCTCCT	ACCGATTGAA	TAAAAAGATG	AAGGTTTAGG
Bd_22-1	AATACGTCCC	TGCCCTTTGT	ACACACCGCC	CGTCGCTCCT	ACCGATTGAA	TAAAAAGATG	AAGGTTTAGG
Go_06-1	AATACGTCCC	TGCCCTTTGT	ACACACCGCC	CGTCGCTCCT	ACCGATTGAA	TAAAAAGATG	AAGGTTTAGG
Go_06-9	AATACGTCCC	TGCCCTTTGT	ACACACCGCC	CGTCGCTCCT	ACCGATTGAA	TAAAAAGATG	AAGGTTTAGG
Go_07-1	AATACGTCCC	TGCCCTTTGT	ACACACCGCC	CGTCGCTCCT	ACCGATTGAA	TAAAAAGATG	AAGGTTTAGG
Go_07-5	AATACGTCCC	TGCCCTTTGT	ACACACCGCC	CGTCGCTCCT	ACCGATTGAA	TAAAAAGATG	AAGGTTTAGG
Go_07-6	AATACGTCCC	TGCCCTTTGT	ACACACCGCC	CGTCGCTCCT	ACCGATTGAA	TAAAAAGATG	AAGGTTTAGG
Go_07-8	AATACGTCCC	TGCCCTTTGT	ACACACCGCC	CGTCGCTCCT	ACCGATTGAA	TAAAAAGATG	AAGGTTTAGG
Go_09-2	AATACGTCCC	TGCCCTTTGT	ACACACCGCC	CGTCGCTCCT	ACCGATTGAA	TAAAAAGATG	AAGGTTTAGG
Go_09-3	AATACGTCCC	TGCCCTTTGT	ACACACCGCC	CGTCGCTCCT	ACCGATTGAA	TAAAAAGATG	AAGGTTTAGG
Go_09-4	AATACGTCCC	TGCCCTTTGT	ACACACCGCC	CGTCGCTCCT	ACCGATTGAA	TAAAAAGATG	AAGGTTTAGG
Go_10-1	AATACGTCCC	TGCCCTTTGT	ACACACCGCC	CGTCGCTCCT	ACCGATTGAA	TAAAAAGATG	AAGGTTTAGG
Go_10-3	AATACGTCCC	TGCCCTTTGT	ACACACCGCC	CGTCGCTCCT	ACCGATTGAA	TAAAAAGATG	AAGGTTTAGG
Go_11-3	AATACGTCCC	TGCCCTTTGT	ACACACCGCC	CGTCGCTCCT	ACCGATTGAA	TAAAAAGATG	AAGGTTTAGG
Go_11-5	AATACGTCCC	TGCCCTTTGT	ACACACCGCC	CGTCGCTCCT	ACCGATTGAA	TAAAAAGATG	AAGGTTTAGG
Go_13-5	AATACGTCCC	TGCCCTTTGT	ACACACCGCC	CGTCGCTCCT	ACCGATTGAA	TAAAAAGATG	AAGGTTTAGG
Go_14-2	AATACGTCCC	TGCCCTTTGT	ACACACCGCC	CGTCGCTCCT	ACCGATTGAA	TAAAAAGATG	AAGGTTTAGG
Go_14-3	AATACGTCCC	TGCCCTTTGT	ACACACCGCC	CGTCGCTCCT	ACCGATTGAA	TAAAAAGATG	AAGGTTTAGG
Go_14-4	AATACGTCCC	TGCCCTTTGT	ACACACCGCC	CGTCGCTCCT	ACCGATTGAA	TAAAAAGATG	AAGGTTTAGG
Pa_06-2	AATACGTCCC	TGCCCTTTGT	ACACACCGCC	CGTCGCTCCT	ACCGATTGAA	TAAAAAGATG	AAGGTTTAGG
Pa_14-4	AATACGTCCC	TGCCCTTTGT	ACACACCGCC	CGTCGCTCCT	ACCGATTGAA	TAAAAAGATG	AAGGTTTAGG
Pa_14-6	AATACGTCCC	TGCCCTTTGT	ACACACCGCC	CGTCGCTCCT	ACCGATTGAA	TAAAAAGATG	AAGGTTTAGG
Pa_16-1	AATACGTCCC	TGCCCTTTGT	ACACACCGCC	CGTCGCTCCT	ACCGATTGAA	TAAAAAGATG	AAGGTTTAGG
Pa_19-1	AATACGTCCC	TGCCCTTTGT	ACACACCGCC	CGTCGCTCCT	ACCGATTGAA	TAAAAAGATG	AAGGTTTAGG
Pa_19-2	AATACGTCCC	TGCCCTTTGT	ACACACCGCC	CGTCGCTCCT	ACCGATTGAA	TAAAAAGATG	AAGGTTTAGG
Pa_19-3	AATACGTCCC	TGCCCTTTGT	ACACACCGCC	CGTCGCTCCT	ACCGATTGAA	TAAAAAGATG	AAGGTTTAGG
Pa_21-2	AATACGTCCC	TGCCCTTTGT	ACACACCGCC	CGTCGCTCCT	ACCGATTGAA	TAAAAAGATG	AAGGTTTAGG
Pa_22-3	AATACGTCCC	TGCCCTTTGT	ACACACCGCC	CGTCGCTCCT	ACCGATTGAA	TAAAAAGATG	AAGGTTTAGG
Pa_39-1	AATACGTCCC	TGCCCTTTGT	ACACACCGCC	CGTCGCTCCT	ACCGATTGAA	TAAAAAGATG	AAGGTTTAGG
Pa_39-5	AATACGTCCC	TGCCCTTTGT	ACACACCGCC	CGTCGCTCCT	ACCGATTGAA	TAAAAAGATG	AAGGTTTAGG
Pa_50-11	AATACGTCCC	TGCCCTTTGT	ACACACCGCC	CGTCGCTCCT	ACCGATTGAA	TAAAAAGATG	AAGGTTTAGG
Pa_50-12	AATACGTCCC	TGCCCTTTGT	ACACACCGCC	CGTCGCTCCT	ACCGATTGAA	TAAAAAGATG	AAGGTTTAGG
Pa_50-19	AATACGTCCC	TGCCCTTTGT	ACACACCGCC	CGTCGCTCCT	ACCGATTGAA	TAAAAAGATG	AAGGTTTAGG
Pa_50-2	AATACGTCCC	TGCCCTTTGT	ACACACCGCC	CGTCGCTCCT	ACCGATTGAA	TAAAAAGATG	AAGGTTTAGG
Pa_57-2	AATACGTCCC	TGCCCTTTGT	ACACACCGCC	CGTCGCTCCT	ACCGATTGAA	TAAAAAGATG	AAGGTTTAGG
Pa_57-3	AATACGTCCC	TGCCCTTTGT	ACACACCGCC	CGTCGCTCCT	ACCGATTGAA	TAAAAAGATG	AAGGTTTAGG
Pa_57-5	AATACGTCCC	TGCCCTTTGT	ACACACCGCC	CGTCGCTCCT	ACCGATTGAA	TAAAAAGATG	AAGGTTTAGG
Pa_62-19	AATACGTCCC	TGCCCTTTGT	ACACACCGCC	CGTCGCTCCT	ACCGATTGAA	TAAAAAGATG	AAGGTTTAGG
Pa_62-3	AATACGTCCC	TGCCCTTTGT	ACACACCGCC	CGTCGCTCCT	ACCGATTGAA	TAAAAAGATG	AAGGTTTAGG
Pa_63-2	AATACGTCCC	TGCCCTTTGT	ACACACCGCC	CGTCGCTCCT	ACCGATTGAA	TAAAAAGATG	AAGGTTTAGG
Pa_63-3	AATACGTCCC	TGCCCTTTGT	ACACACCGCC	CGTCGCTCCT	ACCGATTGAA	TAAAAAGATG	AAGGTTTAGG
Pa_63-4	AATACGTCCC	TGCCCTTTGT	ACACACCGCC	CGTCGCTCCT	ACCGATTGAA	TAAAAAGATG	AAGGTTTAGG
Pa_64-1	AATACGTCCC	TGCCCTTTGT	ACACACCGCC	CGTCGCTCCT	ACCGATTGAA	TGAAGAGATG	AAGGTTTAGG
Pa_64-2	AATACGTCCC	TGCCCTTTGT	ACACACCGCC	CGTCGCTCCT	ACCGATTGAA	TGAAGAGATG	AAGGTTTAGG
Pa_64-3	AATACGTCCC	TGCCCTTTGT	ACACACCGCC	CGTCGCTCCT	ACCGATTGAA	TGAAGAGATG	AAGGTTTAGG
Pa_64-4	AATACGTCCC	TGCCCTTTGT	ACACACCGCC	CGTCGCTCCT	ACCGATTGAA	TGAAGAGATG	AAGGTTTAGG
Pa_79-4	AATACGTCCC	TGCCCTTTGT	ACACACCGCC	CGTCGCTCCT	ACCGATTGAA	TAAAAAGATG	AAGGTTTAGG

```
           1821
Bd_06-10   AGGAATCTGC TACGC-AGAG CCGAATGGAT CTGAATCTCT TTATTTAGAG G
Bd_09-1    AGGAATCTGC TACGC-AGAG CCAAATGGAT CTGAATCTCT TTATTTAGAG G
Bd_09-2    AGGAATCTGC TATGC-AGAG CCAAATGGAT CTGAATCTCT TTATTTAGAG G
Bd_09-3    AGGAATCTGC TACGC-AGAG CCGAATGGAT CTGAATCTCT TTATTTAGAG G
Bd_10-1    AGGAATCTGC TATGC-AGAG CCAAATGGAT CTGAATCTCT TTATTTAGAG G
Bd_10-2    AGGAATCTGC TATGC-AGAG CCAAATGGAT CTGAATCTCT TTATTTAGAG G
Bd_10-2b   AGGAATCTGC TATGC-AGAG CCAAATGGAT CTGAATCTCT TTATTTAGAG G
Bd_11-2    AGGAATCTGC TACGC-AGAG CCGAATGGAT CTGAATCTCT TTATTTAGAG G
Bd_11-6    AGGAATCTGC TACGC-AGAG CCAAATGGAT CTGAATCTCT TTATTTAGAG G
Bd_12-2    AGGAATCTGC TACGC-AGAG CCGAATGGAT CTGAATCTCT TTATTTAGAG G
Bd_13-1    AGGAATCTGC TATGC-AGAG CCAAATGGAT CTGAATCTCT TTATTTAGAG G
Bd_13-4    AGGAATCTGC TATGC-AGAG CCAAATGGAT CTGAATCTCT TTATTTAGAG G
Bd_13-5    AGGAATCTGC TACGC-AGAG CCGAATGGAT CTGAATCTCT TTATTTAGAG G
Bd_14-1    AGGAATCTGC TATGC-AGAG CCAAATGGAT CTGAATCTCT TTATTTAGAG G
Bd_14-2    AGGAATCTGC TATGC-AGAG CCAAATGGAT CTGAATCTCT TTATTTAGAG G
Bd_19-5    AGGAATCTGC TACGC-AGAG CCAAATGGAT CTGAATCTCT TTATTTAGAG G
Bd_19-6    AGGAATCTGC TACGC-AGAG CCAAATGGAT CTGAATCTCT TTATTTAGAG G
Bd_20-1    AGGAATCTGC TATGC-AGAG CCAAATGGAT CTGAATCTCT TTATTTAGAG G
Bd_20-2    AGGAATCTGC TATGC-AGAG CCAAATGGAT CTGAATCTCT TTATTTAGAG G
Bd_22-1    AGGAATCTGC TACGC-AGAG CCGAATGGAT CTGAATCTCT TTATTTAGAG G
Go_06-1    A-GAGTCGAC TATGT-CGAA GCAAATGGAT CTGAATCTCT TTATTTAGAG G
Go_06-9    A-GAGTCGAC TATGT-CGAA GCAAATGGAT CTGAATCTCT TTATTTAGAG G
Go_07-1    A-GAGTCGAC TACGT-CGAA GCAAATGGAT CTGAATCTCT TTATTTAGAG G
Go_07-5    A-GAGTCGAC TATGT-CGAA GCAAATGGAT CTGAATCTCT TTATTTAGAG G
Go_07-6    A-GAGTCGAC TATGT-CGAA GCAAATGGAT CTGAATCTCT TTATTTAGAG G
Go_07-8    A-GAGTCGAC TATGT-CGAA GCAAATGGAT CTGAATCTCT TTATTTAGAG G
Go_09-2    AGGAATCTGC TACGC-AGAG CCAAATGGAT CTGAATCTCT TTATTTAGAG G
Go_09-3    AGGAATCTGC TACGC-AGAG CCGAATGGAT CTGAATCTCT TTATTTAGAG G
Go_09-4    AGGAATCTGC TATGC-AGAG CCAAATGGAT CTGAATCTCT TTATTTAGAG G
Go_10-1    A-GAGTCGAC TATGT-CGAA GCAAATGGAT CTGAATCTCT TTATTTAGAG G
Go_10-3    A-GAGTCGAC TATGT-CGAA GCAAATGGAT CTGAATCTCT TTATTTAGAG G
Go_11-3    A-GAGTCGAC TACGT-CGAA GCAAATGGAT CTGAATCTCT TTATTTAGAG G
Go_11-5    A-GAGTCGAC TATGT-CGAA GCAAATGGAT CTGAATCTCT TTATTTAGAG G
Go_13-5    A-GAGTCGAC TATGT-CGAA GCAAATGGAT CTGAATCTCT TTATTTAGAG G
Go_14-2    A-GAGTCGAC TACGT-CGAA GCAAATGGAT CTGAATCTCT TTATTTAGAG G
Go_14-3    A-GAGTCGAC TATGT-CGAA GCAAATGGAT CTGAATCTCT TTATTTAGAG G
Go_14-4    A-GAGTCGAC TACGT-CGAA GCAAATGGAT CTGAATCTCT TTATTTAGAG G
Pa_06-2    AGGAATCCAT TCATT-GGAA CCAAATGGAT CTGAATCTCT TTATTTAGAG G
Pa_14-4    AGGAATCAGT TCTGC-TGAG CCGAATGGAT CTGAATCTCT TTATTTAGAG G
Pa_14-6    AGGAATCAGT TCTGC-TGAG CCGAATGGAT CTGAATCTCT TTATTTAGAG G
Pa_16-1    AGGAATCACT TCCAG-TGAG CCGAATGGAT CTGAATCTCT TTATTTAGAG G
Pa_19-1    AGGAATCATC TTTGGATGAG CCGAATGGAT CTGAATCTCT TTATTTAGAG G
Pa_19-2    AGGAATCATC TTTGGATGAG CCGAATGGAT CTGAATCTCT TTATTTAGAG G
Pa_19-3    AGGAATCATC TTTGGATGAG CCGAATGGAT CTGAATCTCT TTATTTAGAG G
Pa_21-2    AGGAATCTAC AACGT-AGAA CCAAATGGAT CTGAATCTCT TTATTTAGAG G
Pa_22-3    AGGAATCTAC AACGT-AGAA CCAAATGGAT CTGAATCTCT TTATTTAGAG G
Pa_39-1    AGGAATCACA ATTAG-TGAG CCGAATGGAT CTGAATCTCT TTATTTAGAG G
Pa_39-5    AGGAATCACA ATTAG-TGAG CCGAATGGAT CTGAATCTCT TTATTTAGAG G
Pa_50-11   AGGAATCCAT TCATT-GGAA CCAAATGGAT CTGAATCTCT TTATTTAGAG G
Pa_50-12   AGGAATCCAT TCATT-GGAA CCAAATGGAT CTGAATCTCT TTATTTAGAG G
Pa_50-19   AGGAATCCAT TCATT-GGAA CCAAATGGAT CTGAATCTCT TTATTTAGAG G
Pa_50-2    AGGAATCCAT TCATT-GGAA CCAAATGGAT CTGAATCTCT TTATTTAGAG G
Pa_57-2    AGGAATCCAT TCATT-GGAA CCAAATGGAT CTGAATCTCT TTATTTAGAG G
Pa_57-3    AGGAATCCAT TCATT-GGAA CCAAATGGAT CTGAATCTCT TTATTTAGAG G
Pa_57-5    AGGAATCCAT TCATT-GGAA CCAAATGGAT CTGAATCTCT TTATTTAGAG G
Pa_62-19   AGGAATCATC ACACGATGAG CCGAATGGAT CTGAATCTCT TTATTTAGAG G
Pa_62-3    AGGAATCATC ACACGATGAG CCGAATGGAT CTGAATCTCT TTATTTAGAG G
Pa_63-2    AGGAATCTGC TATGC-AGAA CCAAATGGAT CTGAATCTCT TTATTTAGAG G
Pa_63-3    AGGAATCTGC TATGC-AGAA CCAAATGGAT CTGAATCTCT TTATTTAGAG G
Pa_63-4    AGGAATCAGC ACTAGTTGAA CCGAATGGAT CTGAATCTCT TTATTTAGAG G
Pa_64-1    AGGAATCACC TCTGG-TGAG CCGAATGGAT CTGAATCTCT TTATTTAGAG G
Pa_64-2    AGGAATCACC TCTGG-TGAG CCGAATGGAT CTGAATCTCT TTATTTAGAG G
Pa_64-3    AGGAATCACC TCTGG-TGAG CCGAATGGAT CTGAATCTCT TTATTTAGAG G
Pa_64-4    AGGAATCACC TCTGG-TGAG CCGAATGGAT CTGAATCTCT TTATTTAGAG G
Pa_79-4    AGGAATCATC GTTGA-TGAG CCGAATGGAT CTGAATCTCT TTATTTAGAG G
```
